# Substituted Dibenzodiazocines: Rapid Synthesis and Photochemical Properties

**DOI:** 10.1021/acsomega.1c02524

**Published:** 2021-07-08

**Authors:** Felix Klockmann, Camilla Fangmann, Elena Zender, Tobias Schanz, Claudia Catapano, Andreas Terfort

**Affiliations:** Institute of Inorganic and Analytical Chemistry, University of Frankfurt, Max-von-Laue-Straße 7, 60438 Frankfurt, Germany

## Abstract

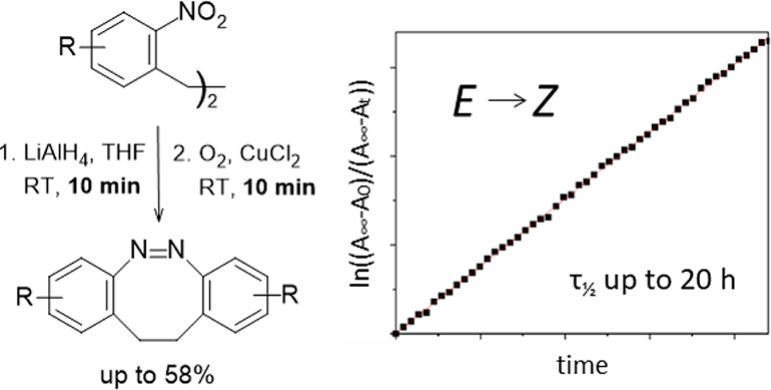

11,12-Dihydrodibenzo[*c*,*g*]-1,2-diazocines have been established as a viable alternative to azobenzene for photoswitching, in particular, as they show an inverted switching behavior: the ground state is the *Z* isomer. In this paper, we present an improved method to obtain dibenzodiazocine and its derivatives from the respective 2-nitrotoluenes in two reaction steps, each proceeding in minutes. This fast access to a variety of derivatives permitted the study of substitution effects on the synthesis and on the photochemical properties. With biochemical applications in mind, methanol was chosen as a protic solvent system for the photochemical investigations. In contrast to the azobenzene system, none of the tested substitution patterns resulted in more efficient switching or in significantly prolonged half-lives, showing that the system is dominated by the ring strain.

## 1 Introduction

Photoswitches have become very useful tools for a wide range of applications, e.g., in pharmacology,^1^ biochemistry,^2^ nanotechnology,^3^ and catalytic chemistry,^4^ because they permit a reagent-free change of conformation with high temporal and spatial control. The probably most popular photochromic molecules are azobenzenes, as they typically offer good switching reversibility together with high photostabilities.^2,5,6^ In this class of molecules, the thermodynamically stable configuration is the *E* state, in which the backbone is almost rod-like, while in the *Z* configuration. it is adopting a V shape, which is typically used to relocate covalently attached moieties.^3,4,7−9^ The time frame, in which experiments can be performed with this photoactivated state, is limited by spontaneous relaxation, the half-life of which is typically in the range of seconds to hours depending on the exact structure of the system, the solvent, and certainly the temperature.^5^ Due to the considerable overlap of the spectra of the *E* and *Z* states, typically no complete transformation to the *Z* state can be attained but rather photostationary states (PSS), in which 5% or more material remains in the *E* configuration.^6^ While this is not much of a problem, if the *Z* state is the biologically active one, it significantly blurs the results of the switching event if the *E* state is the one to be observed.

**Scheme 1:**
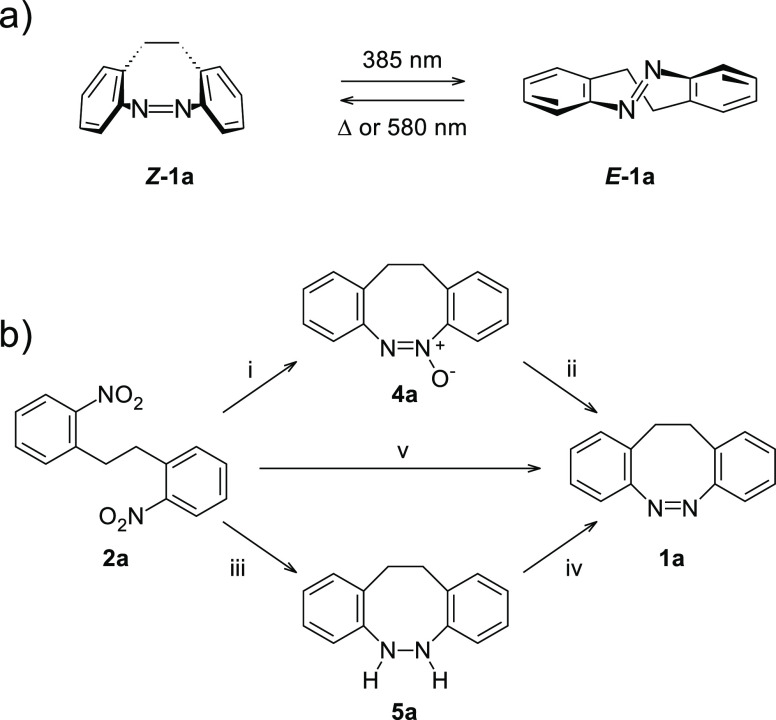
(a) Switching Behavior of 11,12-Dihydrodibenzo[*c*,*g*]-1,2-diazocine **1a**, for which the *Z* Configuration is the Ground State; (b) Strategies Reported in the Literature to Convert 2,2′-Dinitrobibenzyl (**2a**) to **1a** i = Pb, base, MeOH; ii = Ph_3_P, MoCl_2_O_2_, THF or PCl_3_, C_6_H_6_; iii = Zn, Ba(OH)_2_, EtOH; iv = HgO, EtOH or O_2_, CuCl_2_, NaOH, MeOH; v = glucose, NaOH, EtOH, H_2_O or Pb in a ball mill.

An interesting alternative is 11,12-dihydrodibenzo[*c*,*g*]-1,2-diazocine, **1a** (Scheme 1), which is derived from azobenzene by formally inserting an ethylene bridge between the 2-positions at the phenyl rings. This ethylene bridge provides so much strain on the system that the *Z* state becomes the ground state but not enough to inhibit the formation of the *E* state by excitation. Although first described in 1910,^10^ its photochemistry was only recently explored by Herges and Temps.^11,12^ Due to its distortion and the resulting shifts in the absorption spectra, **1a** can be isomerized almost quantitatively (92%) by visible light with high quantum yield and a half-life of about 4.5 h (in hexane at 28.5 °C).^11^ These features gained the interest of the community, resulting in a series of publications.^13−22^ Nevertheless, the number of reported derivatives remains limited and typically only small yields were reported. Basically, two synthetic routes are commonly followed: the reduction of 2,2′-dinitrobibenzyls (**2**)^10,17,23−25^ and the oxidation of 2,2′-diaminobibenzyls,^14,26^ both of which result in the formation of the N=N bond. An interesting new and efficient alternative approach has recently been reported by Staubitz *et al*., who coupled hydrazine derivatives, which already bear the N–N bond, with 2,2′-diiodobibenzyls to obtain a series of new diazocine derivatives.^27^ A very extensive publication by Trauner *et al*. describes a series of mostly nonsymmetrical diazocine derivatives and their photochemical properties.^28^ As with the nonsymmetrical azobenzene derivatives,^5,29,30^ most of the nonsymmetrical diazocines show significantly lowered thermal stability as determined by the half-life times.

In this project, we wanted to study the effect of different substituents in the series of symmetrical dibenzodiazocines to understand the impact of substitution on the photochemical properties and on the respective syntheses. In the parent azobenzene series, strategies have been reported that stabilize the photoexcited state, e.g., by introduction of four halogen atoms or methoxy groups at the *ortho* positions.^30−32^ This kind of substitution also shifts the absorption of the respective isomers into the visible range, which is very advantageous for the application in biological systems.^33^ It would be very useful to also attain similar effects in the diazocine series.

## 2 Results and Discussion

As the symmetric 2,2′-dinitrobibenzyls (**2**) can be very easily obtained by oxidative coupling of the respective 2-nitrotoluenes (**3**),^24,34^ many derivatives of which are readily accessible, we decided to use the reductive ring closure as an entrance to the respective class of diazocines. We consider this advantageous over the oxidative coupling of 2,2′-diaminobibenzyls, as the latter are often obtained from the nitro derivatives by hydrogenation or comparable reduction strategies. For the lead compound, **1a**, three different routes for the reductive ring closure have been established (Scheme 1): the direct reduction,^17,22,24,25,35^ the path *via* the corresponding azoxy compound **4a**,^15,36^ and the one *via* the hydrazine derivative **5a**.^16,22−24,35^ Typical challenges of all the published synthetic strategies are the fair to poor yields, the long reaction times of several hours up to days, or the equipment, which typically cannot be found in organic labs, such as ball mills. Also, often heavy metal reagents are employed, which might interfere with biological systems even in trace amounts.

To our surprise, we found that the same reagent, which is frequently employed for the formation of the acyclic azobenzenes from the respective nitrobenzenes, lithium tetrahydridoaluminate,^37,38^ can be successfully employed for the direct synthesis of diazocine (**1a**) too. When using standard conditions, **1a** was obtained in a yield of 28%, setting the basis for further investigations. As this approach led to the formation of significant amounts of azoxybenzene derivative **4a** as a by-product, we tried to increase the number of hydride equivalents successively. At about 20 equiv, the formation of **4a** could basically be suppressed, but a major side-product now was hydrazobenzene **5a**. In contrast to the azoxybenzene derivative **4a**, a convenient protocol exists for the efficient conversion of the hydrazobenzene **5a** into the diazocine **1a**.^24^ Thus, by exposing the organic phase after the work-up to air in the presence of Cu^2+^, the yield of **1a** could be maximized. We also tried to moderate the reactivity of the LiAlH_4_ by replacing some of the hydrogen atoms with other ligands like alkoxy groups.^39,40^ In addition, alternative complex hydrides were tested, which have been reported for the dimerization of nitroarenes to the analogous azobenzenes.^41−45^ The results are summarized in Table 1.

**Table 1:** Optimization of the Synthesis of Diazocine **1a** Starting from 2,2′-Dinitrobibenzyl **2a**

reducing agent/mol L^–1^^a^		molar ratio to **2**	*T*/°C^b^	time/min^c^	isol. yield/%
LiAlH_4_	2.4	6.25	RT	80	28
LiAlH_4_	2.4	15	RT	10	44
LiAlH_4_	0.2	20	RT	10	53
LiAlH_4_	2.4	20	RT	10	58
LiAlH_4_	2.4	20	40	10	41
LiAlH_4_	2.4	20	0	70	58
LiAlH_4_	2.4	20	–78	170	-
LiAlH_4_^d^	2.4	20	RT	10	22
LiAlH(OMe)_3_	1.7	15	RT	230	48
LiAlH(OMe)_3_	0.9	80	RT	230	33
LiAlH(OMe)_3_	0.2	25	RT	230	40
LiBHEt_3_	1.0	10	RT	230	-e
LiAlH_4_·NMP	0.5	20	RT	230	6
LiAlH_2_(O*t*Bu)_2_	0.5	20	RT	230	11
LiAlH(S*t*Bu)_3_	0.6	20	RT	230	-
LiAlH(SEt)_3_	0.6	20	RT	50	-e
LiBH_4_	0.6	3	RT	230	-

a Solution in THF.

b RT = room temperature.

c After the indicated reaction time, a 10 min reaction time for the reoxidation of the hydrazine side product followed.

d Inverted addition.

e Some formation of respective 2,2′-diaminobibenzyl.

LiAlH_4_ at a high concentration (2.4 mol L^–1^, concentration of the commercial reagent) and room temperature or 0 °C provided **1a** with a yield of 58%, which is the same as the highest yields reported in the literature for the reductive coupling.^24^ When the reaction was run at 0 °C, it was completed after 80 min, while at room temperature, it took only 20 min, including the reoxidation time. This is a significant acceleration as compared to all the literature-known reductive ring closures, which take at least several hours. Of the alternative complex hydrides, only the trimethoxy derivative, LiAlH(OMe)_3_, resulted in acceptable yields.

This efficient method was extended to the synthesis of several derivatives in order to understand the width of applicability and substituent effects. A particular focus was put onto molecules, which can be integrated into biochemical systems *via* suitable linker groups. In contrast to many previous publications,^16,19,27,28^ we decided to use the hydroxyl group rather than the amino group, as we were planning to open a window into carbohydrate chemistry, where the typical functional groups are hydroxyl groups. Consequently, a number of protected OH groups, such as methoxymethoxy (OMOM) or benzyloxy (OBn), were tested together with other substituents, which are known to significantly alter the switching behavior in the acyclic azobenzene series.^5^

**Scheme 2:**
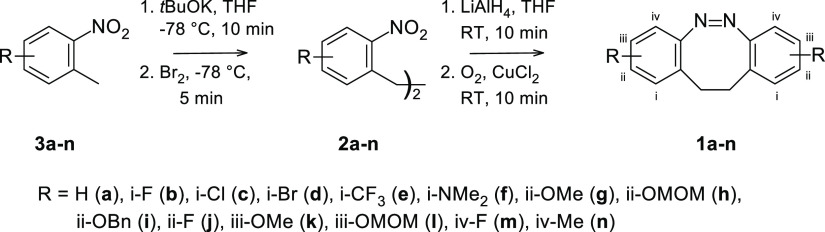
Synthesis of the Symmetrical Dibenzodiazocines **1a–n** with Different Functional Groups in Varying Substitution Patterns by Oxidative Coupling of the 2-Nitrotoluenes **3a–n** Followed by Reductive Ring Closure with LiAlH_4_ For indicating the position, we chose Roman numerals to avoid confusion with the IUPAC nomenclature or other assignments used in this publication.

To this end, a series of 2-nitrotoluene derivatives **3b–n** was oxidatively coupled to form the respective 2,2′-dinitrobibenzyls **2b–n** (Scheme 2), see the Supporting Information for details. For the coupling, we basically used the established routine^24^ with the exception of lowering the reaction temperature to −78 °C, because this provided more reproducible results. The yields for the coupling steps are summarized in Table 2.

**Table 2:** Synthetic Yields and Photochemical Parameters for the Dibenzodiazocines **1a–n** Obtained by Reductive Ring Closure with Complex Metal Hydrides^c^

derivative with R =		yield bibenzyl **2**/%	yield diazocine **1**/%	τ__1/2__/h	% *E* @ PSS_385_
H	**a**	60	58	5.30 ± 0.07	82
i-F	**b**	74	38	7.18 ± 0.02	78
i-Cl	**c**	80	26	10.5 ± 0.2	75
i-Br	**d**	64	20^a^	10.8 ± 0.1	73
i-CF_3_	**e**	79	33^b^	8.44 ± 0.05	77
i-NMe_2_	**f**	65	28	14.3 ± 0.2	28
ii-OMe	**g**	14	10	2.64 ± 0.03	31
ii-OMOM	**h**	21	22	2.21 ± 0.02	25
ii-OBn	**i**	57	3	2.64 ± 0.06	45
ii-F	**j**	71	44	10.2 ± 0.1	41
iii-OMe	**k**	25	20^b^	8.6 ± 0.2	35
iii-OMOM	**l**	36	12	11.3 ± 0.2	37
iv-F	**m**	64	17^a^	14.1 ± 0.2	49
iv-Me	**n**	44	6	20.0 ± 0.2	72

a reduction with LiAlH(OMe)_3_

b These compounds could not be completely purified, see Supp. Info. for the spectra. Nevertheless, the impurities turned out not be photoactive

c Thermal half-life of the *E* isomers and their fraction at PSS at λ = 385 nm were determined at 23 °C by UV/vis spectroscopy in MeOH and ^1^H NMR in CD_3_OD, respectively.

As can be seen, the yields directly correlate with the substituents of the 2-nitrotoluene derivatives: electron-withdrawing groups such as −CF_3_ or −Cl increase the yields, presumably through the increased acidity of the methyl groups, while electron-donating groups typically lower the yields of the 2,2′-dinitrobibenzyls. A notable exception is **2f**, in which the *ortho* dimethylamino group might stabilize the intermediate potassium salt by complexation.

The different 2,2′-dinitrobibenzyl derivatives **2b–n** were subjected to the optimized ring-closure conditions, which in most cases, successfully yielded the respective dibenzodiazocins. Again, clear effects of the substituents could be observed: while the compounds with electron-withdrawing groups, such as −CF_3_ or halogen, generally resulted higher yields (up to 44%), the yields for the compounds with electron-donating groups were lower. A number of special cases have to be discussed: (1) The bromo derivative **2d** lost its bromine atoms when treated with LiAlH_4_, resulting in the formation of the parent compound **1a**. This could be suppressed by using LiAlH(OMe)_3_ instead, which worked satisfactory in the screening study (see Table 1) and is known to tolerate more functional groups than LiAlH_4_.^41^ (2) The benzyloxy compound **3i** became partly cleaved by the hydride ions. In this case, the unwanted side reaction could not be suppressed by the use of the methoxylated aluminate so that the yield never exceeded 3%. (3) In the case of the *ortho* methyl compound **2n**, the yields presumably are low because of the steric hindrance at the nitrogen atoms hampering the ring-closure reaction. (4) A similar effect might play a role for the *ortho* fluorine derivative **2 m**, for which additionally, the formation of significant amounts of the respective 2,2′-diaminobibenzyl was observed. In this case, using LiAlH(OMe)_3_ could minimize the over-reduction and a yield of 17% diazocine **1m** was obtained.

With this series of symmetrical dibenzodiazocines at hand, we set out to explore the photochemical properties. The thermal stability of the excited state and the photoconvertibility are important parameters, in particular, if molecular switches are to be included into larger systems. For many applications, an optimal photoswitch should be quantitatively converted from one state to the other by irradiation. Therefore, the n_+_ → π* transitions in the absorption spectra of the *E* and the *Z* isomers should be energetically well separated. To minimize uncontrolled back-switching during the experiments, the thermal half-life τ__1/2__ should be as long as possible.^32^ As τ__1/2__ significantly depends on the environment, in particular the solvent, it is advisable to determine its value under conditions as close as possible to the latter applications. For the use in biological systems, aqueous buffers would be ideal, but as the simple dibenzodiazocines are not soluble in water, we rather chose methanol as the model solvent for the photochemical experiments.

**Figure 1:**
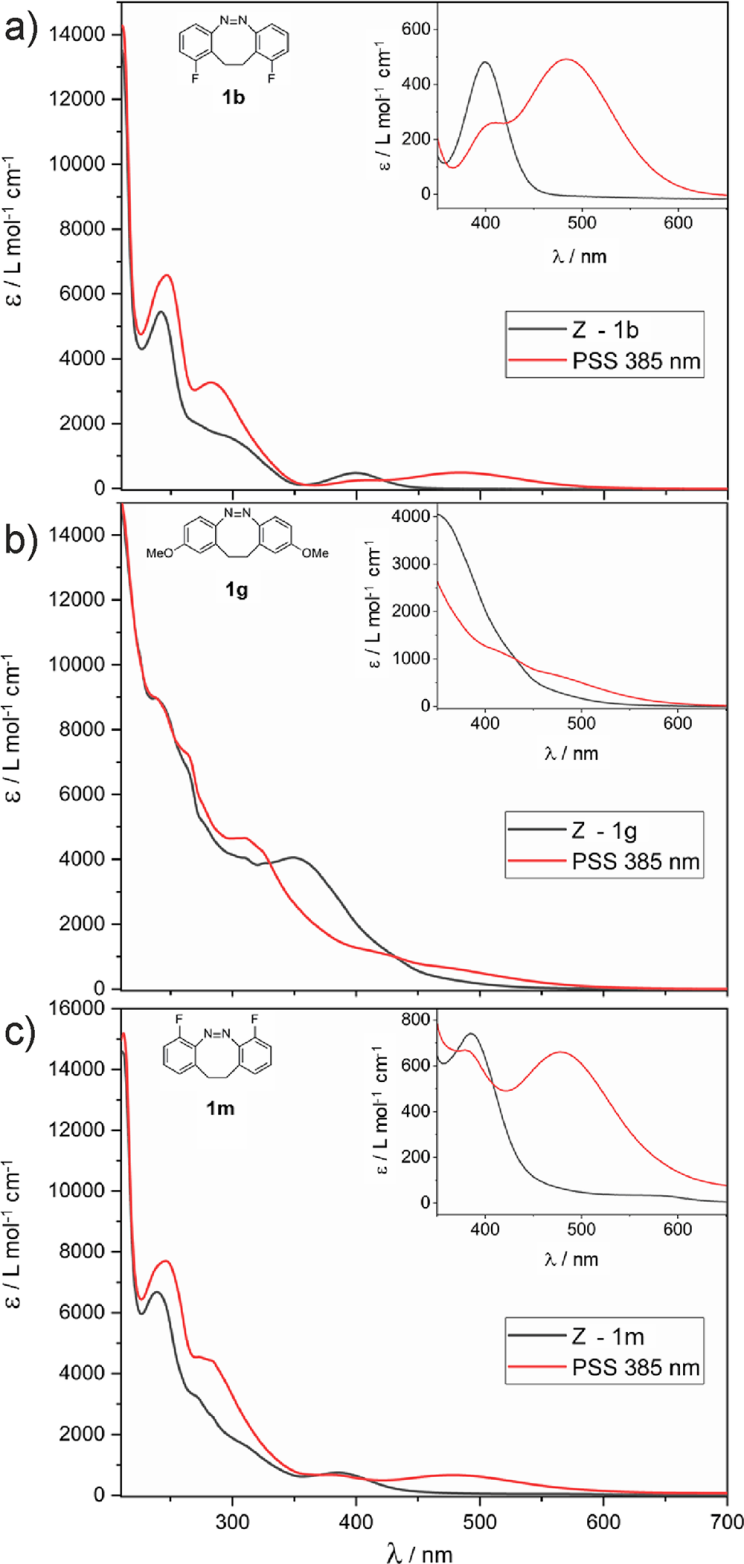
Absorption spectra of representative dibenzodiazocines at the ground state (*Z* isomer; black) and at the photostationary state (PSS; red) at 385 nm, recorded in MeOH. (A) Spectra of 1,10-difluoro-11,12-dihydrodibenzo[*c*,*g*]-1,2-diazocine **1b**. (b) Spectra of 2,9-dimethoxy-11,12-dihydrodibenzo[*c*,*g*]-1,2-diazocine **1g**. (c) Spectra of 4,7-difluoro-11,12-dihydrodibenzo[*c*,*g*]-1,2-diazocine **1m**. Insets show the n_+_ → π* transitions.

We first recorded the UV spectra for the ground state, *Z*-configured isomers, and then the ones of the PSS, in which a dynamic equilibrium between the *E* and the *Z* isomers is established. For the excitation of the system, we chose to use light of 385 nm, as in most of the derivatives, including the parent compound **1a**, the absorption of the *Z* state at this wavelength is significantly higher than for the *E* state, favoring a maximal switching. Representative spectra are shown in Figure 1; the spectra of the other diazocines are depicted in the Supporting Information. The solutions in the PSS were then characterized by ^1^H NMR spectroscopy, where the signals of the two isomers are separated and can be individually integrated to evaluate the composition of the binary mixtures (see Table 2). In a series of separate experiments, the mixtures were permitted to relax thermally (at 23 °C) to the ground state while being observed by UV/vis spectroscopy. In all cases, clean isosbestic points were obtained, confirming the direct switching of the isomers without any intermediate states. The kinetic data were evaluated quantitatively by determination of the absorbances at 480 nm, which is close to the maximum of the n_+_ → π* transition of the *E* isomers. The respective semilogarithmic plots are shown in the Supporting information and yielded the τ__1/2__ for the different derivatives.

It turns out that the substituents have significant impact on the spectra of both states. In the nonsubstituted 11,12-dihydrodibenzo[*c*,*g*]-1,2-diazocine **1a**, the absorption maxima have been reported to be well separated, which is the basis for the excellent performance of the system. As a first trend, it has to be noted that the peak separation in methanol is less pronounced than in the aprotic solvents used in most previous studies. As a result, the fraction of the *E* isomer in the PSS is limited to 82% (Table 2, first entry). The same is true for all the other derivatives tested in this study and presumably is a general trend that needs to be accounted for in other protic solvents, such as water.

In the following, we discuss the effect of the substitutions in the four possible positions with the different functional groups. In the i position, which is *meta* to the azo group (see Scheme 2 for numbering), the separation and intensity of the absorption bands basically stays the same as in the parent compound **1a** for most substituents. This goes in line with previous observations in the azobenzene system, the properties of which are not affected much by *meta*-substituents.^5^ Indeed, the −F group in the position i even induced a slight increase in the molar extinction of the n_+_ → π* absorption of the *Z* isomers while rendering the spectrum of the PSS almost unchanged (Figure 1a). In contrast to this, introducing −NMe_2_ groups into the same position led to a significant reduction of the intensity of the respective band basically to the point that the extinction maxima for both isomers became the same. This certainly also resulted in a decreased switching effect, as in the PSS only 28% of the molecules were converted to the *E* form. We believe that this is caused by the very strong +M effect of this group, but we cannot exclude at this stage that protonation by the solvent might play a role as well. A weakening of the double-bond character of the N=N bond can be excluded in this case, as the half-life of the *E* isomer is clearly longer (14.3 h) than the average life time in this series.

In the ii position, *para* to the azo group, the presence of substituents with +M effect is generally detrimental (**1g–j**). While for the case of the fluorine atoms, the effects are limited (spectra are similar to the ones of **1a**, τ__1/2__ ≈ 10.2 h with 41% of *E* in the PSS), and the spectra of the alkoxy derivatives become almost featureless, as the clear maxima disappear for both, the *Z* and the *E* states (Figure 1b). This observation is in agreement with the situation for azobenzenes, where alkoxy groups in the *para* position lead to deteriorated switching and significantly reduced half-lives (by factors of up to 10^6^), which generally is attributed to the weakening of the N=N double-bond character.^46,47^ As expected, this effect is less pronounced in the position iii, where the half-life of the *E* isomers rises to about 10 h again, although the fractions of *E* isomers in the PSS remain low (∼35%, **1k,l**). This observation is meaningful, as the ii and iii positions are sterically equivalent in the dibenzodiazocine system, which is not the case in the azobenzene system.

Of basic interest are also the substitutions at the iv position, the only available *ortho* position to the azo group in this system. As mentioned before, this kind of substitution results in the azobenzene system in significantly prolonged half-lives and in red-shifted maxima in the spectra.^30−32^ In our case, the fluorine derivative **1m** again shows spectra similar to the one of the lead compound **1a**, only with a significantly increased shoulder at 400 nm for the *E* state (see Figure 1c), which limits the fraction of the *E* isomer in the PSS to 49%. The situation is even worse for the methyl derivative, for which neither the *E* nor the *Z* states show a maximum around 400 nm. Although no positive electronic effect was found, these two derivatives show the longest half-lives, with 14.1 h for **1m** and 20 h for **1n**, demonstrating a significant steric effect, although much smaller than the one found in the azobenzene series.^27−30^

## 3 Conclusions

As the use of lithium tetrahydridoaluminate significantly simplifies the ring-closure reaction of 2,2′-dinitrobibenzyls; we now have a facile route at hand to access dibenzodiazocines in two steps starting from the respective 2-nitrotoluenes. As both reactions can be run on the time scale of minutes and in homogeneous solution, this is the fastest way to this highly interesting class of photoswitches. For the lead compound **1a**, the achievable yield is the same as the best ones previously published for the reductive ring closure. For the substituted derivatives, lower yields were attained, although the derivatives with electron-withdrawing groups show yields up to 44%. In the case of functional groups that become easily cleaved off by LiAlH_4_, a milder alternative was found in LiAlH(OMe)_3_.

The photochemical properties of the new diazocines in methanolic solution were investigated. Similar to acyclic azobenzenes, the variation of the substituents enables the tuning of the photochemistry of diazocines. Halogenation in the *meta* position to the N=N moiety leads to an increased stability of the excited state, whereas a NMe_2_ group at the same site results in an even more stable *E* isomer but leads to a lower photoconversion. A significant stabilization of the *E* state could also be attained by substitution in the *ortho* position. In general, the effects of the substitution on the photochemical properties are much smaller in the diazocine system than for the azobenzenes, e.g., the half-life can only be increased by a factor of four, not by several orders of magnitude. None of the substituents had an advantageous effect on the photoconversion efficiency: the value of 82% for **1a**, which is lower than the one found in aprotic solvents (92%), could not be exceeded by any derivative.

For future incorporation into larger (bio)molecular systems *via* oxygen-containing groups, substitution at the *meta* position (iii) seems to be advisable, as substituents in the *para* position seem to be generally detrimental. The optimized synthetic procedure presented here and the determined impact of substituents on the photochemistry of 11,12-dihydrodibenzo[*c*,*g*]-1,2-diazocine will facilitate their application in further biochemical, pharmacological, and chemical studies.

## 4 Experimental Part

### 4.1 General Information

#### 4.1.1 NMR Spectroscopy

The following NMR spectrometers were used in this study: Bruker AV400 and Bruker DRX600. All measurements were performed at room temperature in deuterated chloroform (CDCl_3_). For calibration, the signal of residual CHCl_3_ was employed. Chemical shifts refer to the δ scale in parts per million (ppm). Coupling constants *J* are given in Hertz (Hz).

#### 4.1.2 Mass Spectrometry

Electrospray ionization mass spectrometry (ESI-MS) spectra were measured on a Thermo Fisher Surveyor MSQ. All high-resolution mass spectrometry (HRMS) spectra were recorded on a Thermo Fisher Scientific LTQ Orbitrap XL.

#### 4.1.3 UV–Vis Spectroscopy

The UV–vis spectra were recorded in a 1.0 cm quartz cuvette using methanol (≥99.8%, HiPerSolv CHROMANORM, VWR) as a solvent.

The thermal half-life of the *E* isomers was determined with an Ocean Optics USB4000 detector, which is connected *via* optical fibers to a CVH100 cuvette holder from Thorlabs and to a DH-mini light source (Ocean Optics). The optical setup was controlled by PHITS (photoswitch irradiator test suite), which was programmed by the Heckel group at the University of Frankfurt.

All other UV–vis spectra were recorded on a Specord S 600 (Analytic Jena).

#### 4.1.4 Light Sources

The samples for the determination of the thermal half-life were irradiated by an M385L2 LED (385 nm; 700 mA) from Thorlabs run with a DC2100 LED driver (Thorlabs).

The evaluation of the photoconvertibility was performed *via* NMR spectroscopy after irradiation with a custom-made 3 W LED (370 nm–380 nm; 750 mA) mounted on a starboard from Avonec.

#### 4.1.5 Chemicals and Column Chromatography

All reactions except the synthesis of 6-(dimethylamino)-2-nitrotoluene were carried out under a nitrogen atmosphere using absolute solvents. DCM was dried over activated 4 Å molecular sieves. THF was first dried with Na and benzophenone and then distilled. LiAlH_4_ suspension in THF (2.40 M) was received from Albemarle.

Silica gel 60 (Macherey–Nagel) had a particle size of 0.063–0.2 mm. TLC plates with a layer thickness of 0.2 mm with a fluorescence indicator from Macherey–Nagel were used.

### 4.2 Syntheses

#### 4.2.1 Syntheses of 2-Nitrotoluene Derivatives **3**

##### 4.2.1.1 6-(Dimethylamino)-2-nitrotoluene **3f**

The synthesis of 6-(dimethylamino)-2-nitrotoluene was performed according to Otevrel and Bobal*.*^48^ To a solution of formaldehyde (37%, 17.0 mL, 120 mmol) and sulfuric acid (20%, 27 mL, 99 mmol) in THF (33 mL) were alternatingly added a solution of 2-methyl-3-nitroaniline (2.17 g, 13.0 mmol) in THF (67 mL) and NaBH_4_ (6.23 g, 184 mmol). After 2 h of stirring at room temperature, the reaction mixture was transferred to a saturated solution of K_2_CO_3_ and then extracted with EtOAc. The combined organic layers were dried over MgSO_4_ and the solvent was removed in vacuum. The product could be isolated quantitatively (2.34 g) as yellow oil.

^1^H NMR (400 MHz, CDCl_3_) δ: 7.49 (dd, *J* = 6.6, 2.7 Hz, 1H), 7.31–7.23 (m, 2H), 2.77 (s, 6H), 2.46 (s, 3H).

ESI-MS: 181.21 [M + H]^+^

HRMS: calcd, 181.09770; found, 181.09722 [M + H]^+^

##### 4.2.1.2 4- and 5-(Methoxymethoxy)-2-nitrotoluene **3h,l**

The introduction of the methoxymethyl group was performed according to Fuji *et al.*(49)

*5-(Methoxymethoxy)-2-nitrotoluene**3h***: To a solution of 3-methyl-4-nitrophenol (10.18 g, 65.45 mmol) in dichloromethane (400 mL) were added first dimethoxymethane (29 mL, 0.33 mol) and then P_2_O_5_ (75 g, 0.53 mol). The reaction mixture was stirred at RT overnight and then decanted. The organic layer was washed with KOH solution and water. The combined organic layers were dried over MgSO_4_ and the solvent was removed in vacuum. The product could be isolated in a yield of 11.5 g (89%) as brown oil. ^1^H NMR (600 MHz, CDCl_3_) δ: 7.66 (d, *J* = 2.6 Hz, 1H), 7.23 (d, *J* = 8.5 Hz, 1H), 7.18 (dd, *J* = 8.5, 2.6 Hz, 1H), 5.19 (s, 2H), 3.48 (s, 3H), 2.52 (s, 3H). ^13^C NMR (101 MHz, CDCl_3_) δ: 160.8, 143.2, 137.0, 127.5, 119.5, 114.1, 94.3, 56.5, 21.7. ESI-MS: 197.93 [M + H]^+^, 196.24 [M-H]^−^. HRMS: calcd, 198.07608; found, 198.07599 [M + H]^+^

*4-(Methoxymethoxy)-2-nitrotoluene **3l***: 4-Methyl-3-nitrophenol was transformed analogously using the procedure described above. The product was isolated in a yield of 5.08 g (79%) as beige solid. ^1^H NMR (400 MHz, CDCl_3_) δ: 7.66 (d, *J* = 2.5 Hz, 1H), 7.23 (d, *J* = 8.5 Hz, 1H), 7.18 (dd, *J* = 8.5, 2.5 Hz, 1H), 5.19 (s, 2H), 3.48 (s, 3H), 2.53 (s, 3H). ^13^C NMR (101 MHz, CDCl_3_) δ: 155.8, 149.8, 133.6, 126.7, 121.6, 112.4, 94.8, 56.4, 19.8. ESI-MS: 197.94 [M + H]^+^. HRMS: calcd, 198.07608; found, 198.07609 [M + H]^+^

#### 4.2.2 General Synthetic Procedure for 2,2′-Dinitrobibenzyls

The dimerization of the 2-nitrotoluenes to the corresponding bibenzyls was performed according to Moormann *et al*.^24^ In contrast to the literature, the best yields were observed at a reaction temperature of −78 °C.

At −78 °C, *t*BuOK solution in THF (1.05 equiv; 1.60 M) was slowly added to a solution of the 2-nitrotoluene derivative in THF (0.2 mM). After stirring for 5 min., Br_2_ (1 equiv) was added dropwise at −78 °C and the mixture was stirred again for 5 min. Then, the cooling was removed and the reaction was stopped by adding saturated aqueous Na_2_SO_3_ solution. The phases were separated and the aqueous one was extracted with DCM. The organic solvent was removed in vacuum and the raw product was further purified. Experimental details, purification, yields, and the spectroscopic data of compounds **2a–n** can be found in the Supporting Information.

#### 4.2.3 General Procedure for the Reductive Ring Closure to Dibenzodiazocines **1a–n**

The corresponding bibenzyl **2** was slowly added to a suspension of LiAlH_4_ in THF (2.4 M, 80 equiv H^–^) at room temperature. After 10 min of stirring, the reaction was stopped by the slow addition of a THF/water mixture (9/1) until the gas evolution ceased. The resulting suspension was filtered and the residue was washed with acetone. CuCl_2_·2H_2_O (0.1 equiv) was added to the filtrate and compressed air was passed through the solution for 10 min. The solvent was removed in vacuum and the raw product was further purified. Experimental details, purification, yields, and the spectroscopic data of compounds **2a–n** can be found in the Supporting Information.

## Supporting Information Available

It contains copies of the UV/vis, ^1^H, and ^13^C NMR spectra of relevant products as well as the experimental kinetic plots for the thermal relaxation (PDF)

## References

[ref1] Hüll K.; Morstein J.; Trauner D. In Vivo Photopharmacology. Chem. Rev. 2018, 118, 10710–10747. 10.1021/acs.chemrev.8b00037. 29985590

[ref2] Lubbe A.; Szymanski W.; Feringa B. L. Recent Developments in Reversible Photoregulation of Oligonucleotide Structure and Function. Chem. Soc. Rev. 2017, 46, 1052–1079. 10.1039/C6CS00461J. 28128377

[ref3] Wang L.; Li Q. Photochromism into Nanosystems: Towards Lighting up the Future Nanoworld. Chem. Soc. Rev. 2018, 47, 1044–1097. 10.1039/C7CS00630F. 29251304

[ref4] Stoll R. S.; Hecht S. Artificial Light-Gated Catalyst Systems. Angew. Chem., Int. Ed. 2010, 49, 5054–5075. 10.1002/anie.201000146. 20712033

[ref5] Bandara H. M. D.; Burdette S. C. Photoisomerization in Different Classes of Azobenzene. Chem. Soc. Rev. 2012, 41, 1809–1825. 10.1039/C1CS15179G. 22008710

[ref6] Beharry A. A.; Woolley G. A. Azobenzene Photoswitches for Biomolecules. Chem. Soc. Rev. 2011, 40, 4422–4437. 10.1039/c1cs15023e. 21483974

[ref7] Baroncini M.; Silvi S.; Credi A. Photo- and Redox-Driven Artificial Molecular Motors. Chem. Rev. 2020, 120, 200–268. 10.1021/acs.chemrev.9b00291. 31415169

[ref8] Weber T.; Chandrasekaran V.; Stamer I.; Thygesen M. B.; Terfort A.; Lindhorst T. K. Switching of bacterial adhesion to a glycosylated surface by reversible reorientation of the carbohydrate ligand. Angew. Chemie Int. Ed. 2014, 53, 14583–14586. 10.1002/anie.201409808. 25429860

[ref9] Chandrasekaran V.; Lindhorst T. K. Sweet Switches: Azobenzene Glycoconjugates Synthesized by Click Chemistry. Chem. Commun. 2012, 48, 7519–7521. 10.1039/c2cc33542e. 22728339

[ref10] Duval H. Recherches sur la Benzidination. III. Recherches dans la série du diphényléthane. Bull. Soc. Chim. Fr. 1910, 7, 727–732.

[ref11] Siewertsen R.; Neumann H.; Buchheim-Stehn B.; Herges R.; Näther C.; Renth F.; Temps F. Highly Efficient Reversible Z–E Photoisomerization of a Bridged Azobenzene with Visible Light through Resolved S_1_ (nπ*) Absorption Bands. J. Am. Chem. Soc. 2009, 131, 15594–15595. 10.1021/ja906547d. 19827776

[ref12] Siewertsen R.; Schönborn J. B.; Hartke B.; Renth F.; Temps F. Superior Z→E and E→Z Photoswitching Dynamics of Dihydrodibenzodiazocine, a Bridged Azobenzene, by S_1_ (nπ*) Excitation at λ = 387 and 490 nm. Phys. Chem. Chem. Phys. 2011, 13, 1054–1063. 10.1039/C0CP01148G. 21072405

[ref13] Thapaliya E. R.; Zhao J.; Ellis-Davies G. C. R. Locked-Azobenzene: Testing the Scope of a Unique Photoswitchable Scaffold for Cell Physiology. ACS Chem. Neurosci. 2019, 10, 2481–2488. 10.1021/acschemneuro.8b00734. 30767510 PMC6657492

[ref14] Cabré G.; Garrido-Charles A.; González-Lafont À.; Moormann W.; Langbehn D.; Egea D.; Lluch J. M.; Herges R.; Alibés R.; Busqué F.; Gorostiza P.; Hernando J. Synthetic Photoswitchable Neurotransmitters Based on Bridged Azobenzenes. Org. Lett. 2019, 21, 3780–3784. 10.1021/acs.orglett.9b01222. 31070376

[ref15] Eljabu F.; Dhruval J.; Yan H. Incorporation of Cyclic Azobenzene into Oligodeoxynucleotides for the Photo-Regulation of DNA Hybridization. Bioorg. Med. Chem. Lett. 2015, 25, 5594–5596. 10.1016/j.bmcl.2015.10.043. 26592170

[ref16] Samanta S.; Qin C.; Lough A. J.; Woolley G. A. Bidirectional Photocontrol of Peptide Conformation with a Bridged Azobenzene Derivative. Angew. Chem., Int. Ed. 2012, 51, 6452–6455. 10.1002/anie.201202383. 22644657

[ref17] Li S.; Han G.; Zhang W. Concise Synthesis of Photoresponsive Polyureas Containing Bridged Azobenzenes as Visible-Light-Driven Actuators and Reversible Photopatterning. Macromolecules 2018, 51, 4290–4297. 10.1021/acs.macromol.8b00687.

[ref18] Zhu Q.; Wang S.; Chen P. Diazocine Derivatives: A Family of Azobenzenes for Photochromism with Highly Enhanced Turn-On Fluorescence. Org. Lett. 2019, 21, 4025–4029. 10.1021/acs.orglett.9b01215. 31084009

[ref19] Löw R.; Rusch T.; Röhricht F.; Magnussen O.; Herges R. Diazocine-Functionalized TATA Platforms. Beilstein J. Org. Chem. 2019, 15, 1485–1490. 10.3762/bjoc.15.150. 31354866 PMC6633206

[ref20] Moormann W.; Langbehn D.; Herges R. Synthesis of functionalized diazocines for application as building blocks in photo- and mechanoresponsive materials. Beilstein J. Org. Chem. 2019, 15, 727–732. 10.3762/bjoc.15.68. 30992720 PMC6444418

[ref21] Moormann W.; Tellkamp T.; Stadler E.; Röhricht F.; Näther C.; Puttreddy R.; Rissanen K.; Gescheidt G.; Herges R. Efficient Conversion of Light to Chemical Energy: Directional, Chiral Photoswitches with Very High Quantum Yields. Angew. Chem., Int. Ed. 2020, 59, 15081–15086. 10.1002/anie.202005361. PMC7496762 32348617

[ref22] Tellkamp T.; Shen J.; Okamoto Y.; Herges R. Diazocines on Molecular Platforms. Eur. J. Org. Chem. 2014, 2014, 5456–5461. 10.1002/ejoc.201402541.

[ref23] Paudler W. W.; Zeiler A. G. Diazocine chemistry. VI. Aromaticity of 5,6-dihydrodibenzo[b,f][1,2]diazocine. J. Org. Chem. 1969, 34, 3237–3239. 10.1021/jo01263a004.

[ref24] Moormann W.; Langbehn D.; Herges R. Solvent-Free Synthesis of Diazocine. Synthesis 2017, 49, 3471–3475. 10.1055/s-0036-1590685.

[ref25] Sell H.; Näther C.; Herges R. Amino-Substituted Diazocines as Pincer-Type Photochromic Switches. Beilstein J. Org. Chem. 2013, 9, 1–7. 10.3762/bjoc.9.1. 23399830 PMC3566864

[ref26] Wang J.; He J.; Zhi C.; Luo B.; Li X.; Pan Y.; Cao X.; Gu H. Highly Efficient Synthesis of Azos Catalyzed by the Common Metal Copper (0) Through Oxidative Coupling Reactions. RSC Adv. 2014, 4, 16607–16611. 10.1039/c4ra00749b.

[ref27] Li S.; Eleya N.; Staubitz A. Cross-Coupling Strategy for the Synthesis of Diazocines. Org. Lett. 2020, 22, 1624–1627. 10.1021/acs.orglett.0c00122. 32009408

[ref28] Maier M. S.; Hüll K.; Reynders M.; Matsuura B. S.; Leippe P.; Ko T.; Schäffer L.; Trauner D. Oxidative Approach Enables Efficient Access to Cyclic Azobenzenes. J. Am. Chem. Soc. 2019, 141, 17295–17304. 10.1021/jacs.9b08794. 31584272

[ref29] Dokić J.; Gothe M.; Wirth J.; Peters M. V.; Schwarz J.; Hecht S.; Saalfrank P. Quantum Chemical Investigation of Thermal Cis-to-Trans Isomerization of Azobenzene Derivatives: Substituent Effects, Solvent Effects, and Comparison to Experimental Data. J. Phys. Chem. A 2009, 113, 6763–6773. 10.1021/jp9021344. 19453149

[ref30] Dong M.; Babalhavaeji A.; Samanta S.; Beharry A. A.; Woolley G. A. Red-Shifting Azobenzene Photoswitches for in Vivo Use. Acc. Chem. Res. 2015, 48, 2662–2670. 10.1021/acs.accounts.5b00270. 26415024

[ref31] Beharry A. A.; Sadovski O.; Woolley G. A. Azobenzene Photoswitching without Ultraviolet Light. J. Am. Chem. Soc. 2011, 133, 19684–19687. 10.1021/ja209239m. 22082305

[ref32] Bléger D.; Schwarz J.; Brouwer A. M.; Hecht S. o-Fluoroazobenzenes as Readily Synthesized Photoswitches Offering Nearly Quantitative Two-Way Isomerization with Visible Light. J. Am. Chem. Soc. 2012, 134, 20597–20600. 10.1021/ja310323y. 23236950

[ref33] Benov L. Photodynamic Therapy: Current Status and Future Directions. Med. Princ. Pract. 2015, 24, 14–28. 10.1159/000362416. 24820409 PMC6489067

[ref34] Enyedy I. J.; Ling Y.; Nacro K.; Tomita Y.; Wu X.; Cao Y.; Guo R.; Li B.; Zhu X.; Huang Y.; Long Y. Q.; Roller P. P.; Yang D.; Wang S. Discovery of Small-Molecule Inhibitors of Bcl-2 through Structure-Based Computer Screening. J. Med. Chem. 2001, 44, 4313–4324. 10.1021/jm010016f. 11728179

[ref35] Deo C.; Bogliotti N.; Métivier R.; Retailleau P.; Xie J. A Visible-Light-Triggered Conformational Diastereomer Photoswitch in a Bridged Azobenzene. Chem. – Eur. J. 2016, 22, 9092–9096. 10.1002/chem.201601400. 27145736

[ref36] Joshi D. K.; Mitchell M. J.; Bruce D.; Lough A. J.; Yan H. Synthesis of Cyclic Azobenzene Analogues. Tetrahedron 2012, 68, 8670–8676. 10.1016/j.tet.2012.06.007.

[ref37] Nystrom R. F.; Brown W. G. Reduction of Organic Compounds by Lithium Aluminum Hydride. III. Halides, Quinones, Miscellaneous Nitrogen Compounds^1^. J. Am. Chem. Soc. 1948, 70, 3738–3740. 10.1021/ja01191a057. 18102934

[ref38] Merino E. Synthesis of Azobenzenes: the Coloured Pieces of Molecular Materials. Chem. Soc. Rev. 2011, 40, 3835–3853. 10.1039/c0cs00183j. 21409258

[ref39] Málek J. Reductions by Metal Alkoxyaluminum Hydrides. Org. React. 2004, 34, 1–99. 10.1002/0471264180.or034.01.

[ref40] Málek J. Reductions by Metal Alkoxyaluminum Hydrides. Part II. Carboxylic Acids and Derivatives, Nitrogen Compounds, and Sulfur Compounds. Org. React. 2004, 36, 249–334. 10.1002/0471264180.or036.03.

[ref41] Brown H. C.; Weissman P. M. Selective Reductions. VII. Reaction of Lithium Trimethoxyaluminohydride with Selected Organic Compounds Containing Representative Functional Groups^1^. J. Am. Chem. Soc. 1965, 87, 5614–5620. 10.1021/ja00952a018.

[ref42] Gaylord N. G. Reduction with Complex Metal Hydrides. J. Chem. Educ. 1957, 34, 367–374. 10.1021/ed034p367.

[ref43] Brown H. C.; Kim S. C.; Krishnamurthy S. Selective Reductions. 26. Lithium Triethylborohydride as an Exceptionally Powerful and Selective Reducing Agent in Organic Synthesis. Exploration of the Reactions with Selected Organic Compounds Containing Representative Functional Groups. J. Org. Chem. 1980, 45, 1–12. 10.1021/jo01289a001.

[ref44] Cha J. S.; Yu S. J. Reaction of Lithium Tris(tert-butylthiolato)hydridoaluminate with Selected Organic Compounds Containing Representative Functional Groups. J. Inclusion Phenom. Macrocyclic Chem. 2009, 65, 7–13. 10.1007/s10847-009-9628-4.

[ref45] Fuller J. C.; Stangeland E. L.; Jackson T. C.; Singaram B. Lithium Aluminum Hydride-N-Methylpyrrolidine Complex. 1. Synthesis and Reactivity of Lithium Aluminum Hydride-N-Methylpyrrolidine Complex. An Air and Thermally Stable Reducing Agent Derived from Lithium Aluminum Hydride. Tetrahedron Lett. 1994, 35, 1515–1518. 10.1016/S0040-4039(00)76746-3.

[ref46] Nishimura N.; Sueyoshi T.; Yamanaka H.; Imai E.; Yamamoto S.; Hasegawa S. Thermal Cis-to-Trans Isomerization of Substituted Azobenzenes II. Substituent and Solvent Effects. Bull. Chem. Soc. Jpn. 1976, 49, 1381–1387. 10.1246/bcsj.49.1381.

[ref47] We wish to thank one of the reviewers of the manuscript for pointing out that the increased flexibility of the eight-membered ring becomes also visible in the ^1^H NMR spectra as coalescence of the methylene signals into one broad singlet.

[ref48] Otevrel J.; Bobal P. Diamine-Tethered Bis(thiourea) Organocatalyst for Asymmetric Henry Reaction. J. Org. Chem. 2017, 82, 8342–8358. 10.1021/acs.joc.7b00079. 28715189

[ref49] Fuji K.; Nakano S.; Fujita E. An Improved Method for Methoxymethylation of Alcohols under Mild Acidic Conditions. Synthesis 1975, 4, 276–277.

