# Supplementary material for: Substituted Dibenzodiazocines: Rapid Synthesis and Photochemical Properties
Source: ACS Omega. 2021 Jul 8;6(28):18434–41. doi: 10.1021/acsomega.1c02524 (PMC8296553; doi:10.1021/acsomega.1c02524)
Supplement: Supplementary file 1 — ao1c02524_si_001.pdf [file ao1c02524_si_001.pdf]

Supporting Information

Substituted Dibenzodiazocines: Rapid Synthesis and Photochemical Properties

Felix Klockmann,[1] Camilla Fangmann,[1] Elena Zender,[1] Tobias Schanz,[1] Claudia Catapano,[1] Andreas Terfort[1,*]

[1] Institute of Inorganic and Analytical Chemistry, University of Frankfurt, Max-von-Laue-Strasse 7, 60438 Frankfurt, Germany)

E-mail: aterfort@chemie.uni-frankfurt.de

Table of contents

1. Experimental Details, Purification, Yields and Spectroscopic Data S2
1.1 Diphenylethanes **2a-n** S2
1.2 Dibenzodiazocines **1a-n** S8
2. UV-Vis spectra and kinetics S14
2.1 UV-Vis spectra of substituted dibenzodiazocines **1c-n** S14
2.2 Determination of thermal half-life of the *E*-isomers S19
3. NMR spectra of synthesized compounds S27

1. Experimental Details, Purification, Yields and Spectroscopic Data

1.1 Diphenylethanes 2a-n

1,2-Bis(2-nitrophenyl)ethane 2a

2-Nitrotoluene (2.73 g, 191.9 mmol), t-BuOK in THF (1.60 M, 13 mL, 21 mmol) and bromine (1.0 mL, 20 mmol) were reacted.

^{1}H NMR (400 MHz, $CDCl_3$): δ 7.96 (d, *J* = 8.1 Hz, 2H), 7.54 (t, *J* = 7.5 Hz, 2H), 7.40 (m, 4H), 3.25 (s, 4H).

Purification:	excess 2-nitrotoluene was removed in vacuum at 80 °C. The residue was dissolved in DCM and filtered through a pad of silica gel.
Appearance:	yellow solid
Yield:	1.63 g, 60%

1,2-Bis(2-fluoro-6-nitrophenyl)ethane 2b

2-Fluoro-6-nitrotoluene (3.00 g, 19.3 mmol), t-BuOK in THF (1.60 M, 24 mL, 38 mmol) and bromine (1.0 mL, 20 mmol) were employed.

^{1}H NMR (400 MHz, $CDCl_3$): δ 7.70 (d, *J* = 8.1 Hz, 2H), 7.36 (td, *J* = 8.2, 5.4 Hz, 2H), 7.27 (t, *J* = 8.1 Hz, 2H), 3.32 (s, 4H).

^{13}C NMR (101 MHz, $CDCl_3$): δ 161.6 (d, *J* = 249.6 Hz), 150.6 (d, *J* = 4.8 Hz), 128.4 (d, *J* = 9.7 Hz), 123.7 (d, *J* = 20.3 Hz), 120.5 (d, *J* = 3.4 Hz), 120.1 (d, *J* = 24.1 Hz), 25.0 (dd, *J* = 2.9, 1.4 Hz).

ESI-MS:	308.97 $[M+H]^+$	
	330.92 $[M+Na]^+$	
HRMS:	found	309.06812 $[M+H]^+$
	calc.	309.06814
Purification:	column chromatography (DCM)	
Appearance:	yellow solid	
Yield:	2.20 g, 74%	

1,2-Bis(2-chloro-6-nitrophenyl)ethane 2c

2-Chloro-6-nitrotoluene (5.20 g, 29.1 mmol), THF (55 mL), t-BuOK in THF (1.60 M, 36 mL, 58 mmol) and bromine (1.5 mL, 29 mmol) were used.

^{1}H NMR (400 MHz, $CDCl_3$): δ 7.64 (dd, *J* = 8.1, 1.2 Hz, 2H), 7.60 (dd, *J* = 8.1, 1.2 Hz, 2H), 7.32 (t, *J* = 8.1 Hz, 2H), 3.39 (s, 4H).

^{13}C NMR (101 MHz, $CDCl_3$): δ 151.8, 137.0, 133.9, 132.5, 128.3, 123.1, 28.3.

ESI-MS:	341.00 $[M+H]^+$	
HRMS:	found	340.00121 $[M]^+$
	calc.	340.00176
Purification:	filtered through a pad of silica gel with DCM as eluent	
Appearance:	orange solid	
Yield:	3.97 g, 80%	

1,2-Bis(2-bromo-6-nitrophenyl)ethane 2d

2-Bromo-6-nitrotoluene (4.02 g, 18.6 mmol), t-BuOK in THF (1.60 M, 12.5 mL, 20.0 mmol) and bromine (1.0 mL, 19 mmol) were reacted.

^{1}H NMR (400 MHz, $CDCl_3$): δ 7.78 (d, *J* = 8.0 Hz, 2H), 7.66 (d, *J* = 8.1 Hz, 2H), 7.24 (t, *J* = 8.1 Hz, 2H), 3.43 (s, 4H).

^{13}C NMR (101 MHz, $CDCl_3$): δ 151.7, 137.3, 133.8, 128.6, 127.4, 123.8, 30.8.

ESI-MS:	431.16 $[M+H]^+$	
HRMS:	found	430.93768 $[M+H]^+$
	calc.	430.90596
	found	452.88742 $[M+Na]^+$
	calc.	452.88791
Remarks:	The synthesis was performed under exclusion of light.	
Purification:	column chromatography (cyclohexane/EtOAc = 5/1)	
Appearance:	yellow solid	
Yield:	2.54 g, 64%	

1,2-Bis(6-trifluoromethyl-2-nitrophenyl)ethane 2e

6-Trifluoromethyl-2-nitrotoluene (4.05 g, 19.7 mmol), t-BuOK in THF (1.60 M, 13 mL, 21 mmol) and bromine (1.0 mL, 20 mmol) reacted under the conditions described above.

^{1}H NMR (400 MHz, $CDCl_3$): δ 7.90 (d, *J* = 8.0 Hz, 2H), 7.86 (d, *J* = 8.1 Hz, 2H), 7.53 (t, *J* = 8.0 Hz, 2H), 3.43 (s, 4H).

^{13}C NMR (101 MHz, $CDCl_3$): δ 152.3 (s), 133.1 (s), 131.6 (q, *J* = 30.6 Hz), 130.3 (s), 128.3 (s), 128.1 (s), 123.4 (q, *J* = 274.6 Hz), 27.8 (s).

ESI-MS: 339.12 $[M-CF_3]^+$

431.15 $[M+Na]^+$

HRMS: found 431.04974 $[M+Na]^+$

calc. 431.04370

found 447.01737 $[M+K]^+$

calc. 447.01763

Purification: column chromatography (cyclohexane/EtOAc = 3/1)

Appearance: orange solid

Yield: 3.18 g, 79%

1,2-Bis(2-dimethylamino-6-nitrophenyl)ethane 2f

3f (1.83 g, 10.2 mmol) was reacted with t-BuOK in THF (1.60 M, 13 mL, 20 mmol) and bromine (0.52 mL, 10 mmol).

^{1}H NMR (400 MHz, $CDCl_3$): δ 7.47 – 7.38 (m, 2H), 7.22 – 7.15 (m, 4H), 3.25 (s, 4H), 2.50 (s, 12H).

^{13}C NMR (101 MHz, $CDCl_3$): δ 155.4, 151.9, 131.3, 127.1, 124.5, 119.4, 45.2, 26.3.

ESI-MS: 359.04 $[M+H]^+$

HRMS: found 358.16356 $[M]^+$

calc. 358.16411

Purification: column chromatography (*n*-hexane/EtOAc = 4/1)

Appearance: yellow solid

Yield: 1.19 g, 65%

1,2-Bis(5-methoxy-2-nitrophenyl)ethane 2g

5-Methoxy-2-nitrotoluene (4.98 g, 29.8 mmol), t-BuOK in THF (1.60 M, 37 mL, 60 mmol) and bromine (1.5 mL, 30 mmol) were employed.

^{1}H NMR (400 MHz, $CDCl_3$): δ 8.09 (d, J = 9.1 Hz, 2H), 6.89 (d, J = 2.6 Hz, 2H), 6.84 (dd, J = 9.1, 2.6 Hz, 2H), 3.89 (s, 6H), 3.29 (s, 4H).

^{13}C NMR (126 MHz, $CDCl_3$): δ 163.5, 142.1, 139.8, 127.9, 116.8, 113.2, 56.0, 35.4.

ESI-MS: 355.05 $[M+Na]^+$

330.98 $[M-H]^-$

HRMS: found 355.09015 $[M+Na]^+$

calc. 355.09006

HRMS: found 371.06409 $[M+K]^+$

calc. 371.06399

Purification: column chromatography (*n*-hexane/EtOAc = 1/1)

Appearance: beige solid

Yield: 693 g, 14%

1,2-Bis(5-(methoxymethoxy)-2-nitrophenyl)ethane 2h

3h (4.98 g, 25.4 mmol), THF (60 mL), t-BuOK in THF (1.60 M, 32 mL, 51 mmol) and bromine (1.3 mL, 25 mmol) were used.

^{1}H NMR (400 MHz, $CDCl_3$): δ 8.05 (d, *J* = 9.9 Hz, 2H), 7.04 – 6.88 (m, 4H), 5.22 (s, 4H), 3.48 (s, 6H), 3.28 (s, 4H).

^{13}C NMR (101 MHz, $CDCl_3$): δ 161.0, 143.0, 139.4, 127.8, 119.4, 114.5, 94.4, 56.6, 35.0.

ESI-MS: 414.89 $[M+Na]^+$

HRMS: found 415.11089 $[M+Na]^+$

calc. 415.11119

Purification: gradient sublimation (185 °C, 10^{-5} mbar, 5 h)

Appearance: beige solid

Yield: 1.05 g, 21%

1,2-Bis(5-benzyloxy-2-nitrophenyl)ethane 2i

5-Benzyloxy-2-nitrotoluene (1.51 g, 6.17 mmol), t-BuOK in THF (1.60 M, 7.7 mL, 12 mmol) and bromine (0.3 mL, 6 mmol) were used.

^{1}H NMR (400 MHz, $CDCl_3$): δ 8.08 (d, *J* = 9.1 Hz, 2H), 7.51 – 7.32 (m, 10H), 6.99 (d, *J* = 2.7 Hz, 2H), 6.91 (dd, *J* = 9.1, 2.7 Hz, 2H), 5.14 (s, 4H), 3.27 (s, 4H).

^{13}C NMR (101 MHz, $CDCl_3$): δ 162.6, 142.2, 139.8, 135.7, 128.9, 128.6, 127.9, 127.8, 117.8, 113.9, 70.7, 35.4.

ESI-MS: 507.19 $[M+Na]^+$

HRMS: found 507.15042 $[M+Na]^+$

calc. 507.15266

Purification: gradient sublimation (130 °C, 10^{-5} mbar, 2.5 h)

Appearance: brownish solid

Yield: 852 mg, 57%

1,2-Bis(3-fluoro-6-nitrophenyl)ethane 2j

5-Fluor-2-nitrotoluene (2.00 g, 12.9 mmol) was reacted with t-BuOK in THF (1.60 M, 8.5 mL, 14 mmol) and bromine (0.7 mL, 14 mmol).

^{1}H NMR (400 MHz, $CDCl_3$): δ 8.08 (dd, *J* = 9.0, 5.1 Hz, 2H), 7.14 (dd, *J* = 8.8, 2.7 Hz, 2H), 7.12 – 7.05 (m, 2H), 3.28 (s, 4H).

^{13}C NMR (101 MHz, $CDCl_3$): δ 165.0 (d, *J* = 257.6 Hz), 145.3 (s), 139.6 (d, *J* = 9.0 Hz), 128.1 (d, *J* = 10.0 Hz), 119.2 (d, *J* = 23.1 Hz), 115.1 (d, *J* = 23.2 Hz), 34.6 (s).

HRMS: found 309.06767 $[M+H]^+$

calc. 309.06814

Purification: column chromatography (cyclohexane/EtOAc = 5/1)

Appearance: yellow solid

Yield: 1.41 g, 71%

1,2-Bis(4-methoxy-2-nitrophenyl)ethane 2k

4-Methoxy-2-nitrotoluene (0.36 g, 2.2 mmol), t-BuOK in THF (1.60 M, 2.7 mL, 4.3 mmol) and bromine (0.11 mL, 2.2 mmol) were used.

^{1}H NMR (400 MHz, $CDCl_3$): δ 7.46 (d, *J* = 2.7 Hz, 2H), 7.28 (d, *J* = 8.5 Hz, 2H), 7.08 (dd, *J* = 8.5, 2.7 Hz, 2H), 3.86 (s, 6H), 3.14 (s, 4H).

^{13}C NMR (101 MHz, $CDCl_3$): δ 158.6, 149.7, 133.5, 128.3, 120.3, 109.4, 56.0, 34.1.

ESI-MS: 354.87 $[M+Na]^+$

HRMS: found 332.10029 $[M]^+$

calc. 332.10000

Purification: column chromatography (*n*-hexane/EtOAc = 4/1)

Appearance: yellow solid

Yield: 91 mg, 25%

1,2-Bis(4-(methoxymethoxy)-2-nitrophenyl)ethane 2l

The compound was formed from **3l** (2.50 g, 12.6 mmol), t-BuOK in THF (1.60 M, 16 mL, 25 mmol) and bromine (0.70 mL, 13 mmol).

^{1}H NMR (400 MHz, $CDCl_3$): δ 7.63 (d, *J* = 2.6 Hz, 2H), 7.32 (d, *J* = 8.5 Hz, 2H), 7.22 (dd, *J* = 8.5, 2.6 Hz, 2H), 5.21 (s, 4H), 3.49 (s, 6H), 3.13 (s, 4H).

^{13}C NMR (101 MHz, $CDCl_3$): δ 156.2, 149.6, 133.4, 129.3, 121.8, 112.4, 94.8, 56.4, 34.1.

ESI-MS: 414.99 $[M+Na]^+$

HRMS: found 415.11078 $[M+Na]^+$

calc. 415.11119

Purification: gradient sublimation (180 °C, 10^{-5} mbar, 1.5 h)

Appearance: brown solid

Yield: 890 mg, 36%

1,2-Bis(3-fluoro-2-nitrophenyl)ethane 2m

3-Fluor-2-nitrotoluene (1.50 g, 9.65 mmol), t-BuOK in THF (1.60 M, 12 mL, 19 mmol) and bromine (0.50 mL, 9.8 mmol) were employed.

^{1}H NMR (400 MHz, $CDCl_3$): δ 7.42 (td, *J* = 8.1, 5.3 Hz, 2H), 7.15 (t, *J* = 8.9 Hz, 2H), 7.07 (d, *J* = 7.8 Hz, 2H), 2.99 (s, 4H).

^{13}C NMR (101 MHz, $CDCl_3$): δ 154.1 (d, *J* = 258.1 Hz), 140.0 (d, *J* = 10.3 Hz), 135.1 (d, *J* = 1.4 Hz), 132.4 (d, *J* = 8.3 Hz), 126.4 (d, *J* = 3.6 Hz), 115.7 (d, *J* = 19.2 Hz), 32.9 (d, *J* = 1.8 Hz).

ESI-MS: 308.97 $[M+H]^+$
330.95 $[M+Na]^+$

HRMS: found 309.06744 $[M+H]^+$
calc. 309.06814

Purification: gradient sublimation (125 °C, 10^{-5} mbar, 2 h)

Appearance: yellow solid

Yield: 952 mg, 64%

1,2-Bis(3-methyl-2-nitrophenyl)ethane 2n

2-Nitro-m-xylene (1.00 g, 6.62 mmol) was reacted with t-BuOK in THF (1.60 M, 4.3 mL, 7.0 mmol) and bromine (0.30 mL, 6.6 mmol).

^{1}H NMR (400 MHz, $CDCl_3$): δ 7.29 (t, ^{3}J = 7.6 Hz, 2H), 7.16 (d, ^{3}J = 7.7 Hz, 2H), 7.09 (d, ^{3}J = 7.7 Hz, 2H), 2.85 (s, 4H), 2.33 (s, 6H).

^{13}C-NMR (101 MHz, $CDCl_3$): δ 151.72, 132.28, 130.38, 129.81, 129.68, 128.53, 33.21, 17.61.

ESI-MS: 300.33 $[M]^+$

HRMS: found 323.10006 $[M+Na]^+$
calc. 323.10023

Purification: gradient sublimation (110 °C, 10^{-5} mbar, 15 h)

Appearance: yellow solid

Yield: 437 mg, 44%

1.2 Dibenzodiazocines 1a-n

11,12-Dihydrodibenzo[*c,g*]-1,2-diazocine 1a

2a (404 mg, 1.48 mmol) was reacted with $LiAlH_4$ in THF (2.40 M, 12 mL, 29 mmol).

^{1}H NMR (600 MHz, $CDCl_3$): δ 7.13 (t, *J* = 7.5 Hz, 2H), 7.01 (t, *J* = 7.4 Hz, 2H), 6.97 (d, *J* = 7.4 Hz, 2H), 6.82 (d, *J* = 7.8 Hz, 2H), 2.88 (m, 4H).

Purification: column chromatography (*n*-hexane/EtOAc = 4/1)

Appearance: yellow solid

Yield: 179 mg, 58%

1,10-Difluoro-11,12-dihydrodibenzo[*c,g*]-1,2-diazocine 1b

2b (1.00 g, 3.24 mmol) and $LiAlH_4$ in THF (2.40 M, 27 mL, 65 mmol) were reacted.

^{1}H NMR (400 MHz, $CDCl_3$): δ 7.12 (td, *J* = 8.0, 5.7 Hz, 2H), 6.81 (t, *J* = 8.8 Hz, 2H), 6.66 (d, *J* = 7.9 Hz, 2H), 3.30 – 3.12 (m, 2H), 2.74 – 2.56 (m, 2H).

^{13}C NMR (101 MHz, $CDCl_3$): δ 160.4 (d, *J* = 247.7 Hz), 156.7 (d, *J* = 3.1 Hz), 127.8 (d, *J* = 9.2 Hz), 116.0 (d, *J* = 18.3 Hz), 114.8 (d, *J* = 3.7 Hz), 113.7 (d, *J* = 23.4 Hz), 22.7 (d, *J* = 3.6 Hz).

ESI-MS: 245.07 $[M+H]^+$

HRMS: found 245.08896 $[M+H]^+$

calc. 245.08848

Purification: column chromatography (*n*-hexane/EtOAc = 3/1)

Appearance: yellow solid

Yield: 301 mg, 38%

1,10-Dichloro-11,12-dihydrodibenzo[*c,g*]-1,2-diazocine 1c

2c (585 mg, 1.47 mmol) and $LiAlH_4$ in THF (2.40 M, 12.2 mL, 29.3 mmol) were employed.

^{1}H NMR (400 MHz, $CDCl_3$): δ 7.14 (dd, *J* = 8.0, 1.2 Hz, 2H), 7.07 (t, *J* = 7.9 Hz, 2H), 6.74 (dd, *J* = 7.8, 1.2 Hz, 2H), 3.50 – 3.29 (m, 2H), 2.86 – 2.69 (m, 2H).

^{13}C NMR (101 MHz, $CDCl_3$): δ 156.4, 134.7, 128.2, 127.5, 126.1, 117.5, 27.5.

HRMS: found 276.02156 $[M]^+$

calc. 276.02210

Purification: column chromatography (*n*-hexane/EtOAc = 4/1)

Appearance: yellow solid

Yield: 106 mg, 26%

1,10-Dibromo-11,12-dihydrodibenzo[*c*,*g*]-1,2-diazocine 1d

LiAlH(OMe)$_3$ was first formed by reaction of LiAlH$_4$ in THF (2.40 M, 10.7 mL, 25.6 mmol) with MeOH (3.12 mL, 76.8 mmol) at 0 °C, before **2d** (550 mg, 1.28 mmol) was added. The rest of the reaction proceeded like decribed above.

^{1}H NMR (400 MHz, CDCl$_3$): δ 7.33 (d, *J* = 8.0 Hz, 2H), 6.99 (t, *J* = 7.9 Hz, 2H), 6.76 (d, *J* = 7.8 Hz, 2H), 3.47 – 3.27 (m, 2H), 2.88 – 2.71 (m, 2H).

^{13}C NMR (101 MHz, CDCl$_3$): δ 156.1, 131.5, 127.9, 127.8, 125.4, 118.1, 30.5.

HRMS: found 366.92672 [M+H]$^+$
calc. 366.92630

Remarks: LiAlH(OMe)$_3$ (20 eq. H$^-$) was used instead of LiAlH$_4$. The reagent was prepared according to chapter 2.1.

Purification: gradient sublimation (125 °C, 10^{-5} mbar, 1.5 h)

Appearance: orange solid

Yield: 94 mg, 20%

1,10-Bis(trifluoromethyl)-11,12-dihydrodibenzo[*c*,*g*]-1,2-diazocine 1e

2e (820 mg, 2.00 mmol) and LiAlH$_4$ in THF (2.40 M, 17 mL, 40 mmol) were employed.

^{1}H NMR (400 MHz, CDCl$_3$): δ 7.42 (d, *J* = 7.9 Hz, 2H), 7.24 (t, *J* = 7.9 Hz, 2H), 6.96 (d, *J* = 7.9 Hz, 2H), 3.37 – 3.21 (m, 2H), 2.92 – 2.76 (m, 2H).

^{13}C NMR (126 MHz, CDCl$_3$): δ 156.86 (s), 130.24 – 129.20 (m), 126.96 (s), 126.44 (s), 125.06 – 124.88 (m), 123.70 (q, *J* = 274.7 Hz), 121.71 (s), 26.58 (s).

ESI-MS: 344.15 [M]$^+$

HRMS: found 345.08215 [M+H]$^+$
calc. 345.08209

Purification: gradient sublimation (125 °C, 10^{-5} mbar, 3.5 h)

Appearance: yellow solid

Yield: 227 mg, 33%. This product contained some photo-inactive impurities, see the spectra in the Supp. Info.

1,10-Bis(dimethylamino)-11,12-dihydrodibenzo[*c*,*g*]-1,2-diazocine 1f

2f (118 mg, 0.279 mmol) and LiAlH$_4$ in THF (2.40 M, 2.3 mL, 5.6 mmol) were used.

^{1}H NMR (400 MHz, CDCl$_3$): δ 7.02 (t, *J* = 7.9 Hz, 2H), 6.75 (dd, *J* = 8.0, 0.9 Hz, 2H), 6.44 (dd, *J* = 7.8, 0.9 Hz, 2H), 3.53 – 3.34 (m, 2H), 2.65 – 2.46 (m, 2H).

^{13}C NMR (101 MHz, CDCl$_3$): δ 157.7, 153.1, 126.8, 123.8, 117.1, 112.7, 45.0, 24.9.

ESI-MS: 295.13 $[M+H]^+$

HRMS: found 295.19189 $[M+H]^+$

calc. 295.19172

Purification: column chromatography (*n*-hexane/EtOAc = 2/1)

Appearance: yellow solid

Yield: 23 mg, 28%

2,9-Dimethoxy-11,12-dihydrodibenzo[*c*,*g*]-1,2-diazocine 1g

Obtained by reaction of **2g** (201 mg, 0.602 mmol) with $LiAlH_4$ in THF (2.40 M, 5.0 mL, 12 mmol).

^{1}H NMR (400 MHz, $CDCl_3$): δ 6.81 (d, *J* = 8.6 Hz, 2H), 6.68 (dd, *J* = 8.6, 2.6 Hz, 2H), 6.52 (d, *J* = 2.6 Hz, 2H), 3.72 (s, 6H), 2.83 (s, 4H).

^{13}C NMR (101 MHz, $CDCl_3$): δ 158.3, 149.2, 129.8, 121.2, 114.6, 112.0, 55.5, 32.2.

ESI-MS: 269.15 $[M+H]^+$

HRMS: found 269.12836 $[M+H]^+$

calc. 269.12845

Purification: column chromatography (*n*-hexane/EtOAc = 1/1)

Appearance: orange solid

Yield: 16 mg, 10%

2,9-Bis(methoxymethoxy)-11,12-dihydrodibenzo[*c*,*g*]-1,2-diazocine 1h

2h (500 mg, 1.27 mmol) and $LiAlH_4$ in THF (2.40 M, 11 mL, 26 mmol) were used.

^{1}H NMR (600 MHz, $CDCl_3$): δ 6.87 – 6.77 (m, 4H), 6.67 (d, *J* = 2.1 Hz, 2H), 5.08 (s, 4H), 3.44 (s, 6H), 2.83 (bs, 4H).

^{13}C NMR (151 MHz, $CDCl_3$): δ 156.1, 150.1, 129.7, 121.2, 117.0, 114.6, 94.7, 56.2, 32.1.

ESI-MS: 329.20 $[M+H]^+$

HRMS: found 329.15016 $[M+H]^+$

calc. 329.14958

Purification: column chromatography (*n*-hexane/EtOAc = 2/1)

Appearance: yellow solid

Yield: 92 mg, 22%

2,9-Dibenzyloxy-11,12-dihydrodibenzo[*c*,*g*]-1,2-diazocine 1i

2i (310 mg, 0.640 mmol) was reduced with $LiAlH_4$ in THF (2.40 M, 5.5 mL, 13 mmol).

^{1}H NMR (400 MHz, $CDCl_3$): δ 7.42 – 7.34 (m, 10H), 6.82 (d, *J* = 8.6 Hz, 2H), 6.76 (dd, *J* = 8.6, 2.5 Hz, 2H), 6.62 (d, *J* = 2.5 Hz, 2H), 4.97 (s, 4H), 2.82 (bs, 4H).

^{13}C NMR (101 MHz, $CDCl_3$): δ 157.6, 149.4, 136.8, 129.9, 128.7, 128.2, 127.7, 121.2, 115.7, 112.9, 70.3, 32.2.

ESI-MS: 421.15 $[M+H]^+$

HRMS: found 421.19105 $[M+H]^+$

calc. 421.19099

Purification: column chromatography (*n*-hexane/EtOAc = 4/1)

Appearance: yellow solid

Yield: 8 mg, 3%

2,9-Difluoro-11,12-dihydrodibenzo[*c,g*]-1,2-diazocine 1j

2j (480 mg, 1.56 mmol) and $LiAlH_4$ in THF (2.40 M, 13 mL, 31 mmol) were utilized.

^{1}H NMR (400 MHz, $CDCl_3$): δ 6.91 – 6.78 (m, 4H), 6.72 (dd, *J* = 9.2, 2.4 Hz, 2H), 2.96 (bs, 2H), 2.74 (bs, 2H).

^{13}C NMR (101 MHz, $CDCl_3$): δ 161.2 (d, *J* = 246.6 Hz), 151.5 (d, *J* = 3.1 Hz), 130.3 (d, *J* = 7.8 Hz), 121.0 (d, *J* = 8.8 Hz), 116.3 (d, *J* = 22.4 Hz), 114.0 (d, *J* = 22.6 Hz), 31.7 (d, *J* = 1.3 Hz).

HRMS: found 245.08874 $[M+Na]^+$

calc. 245.08848

Purification: column chromatography (*n*-hexane/EtOAc = 4/1)

Appearance: orange solid

Yield: 168 mg, 44%

3,8-Dimethoxy-11,12-dihydrodibenzo[*c,g*]-1,2-diazocine 1k

2k (96.7 mg, 0.301 mmol) was reacted with $LiAlH_4$ in THF (2.40 M, 2.5 mL, 6.0 mmol).

^{1}H NMR (400 MHz, $CDCl_3$): δ 6.87 (d, *J* = 8.4 Hz, 2H), 6.57 (dd, *J* = 8.4, 2.6 Hz, 2H), 6.38 (d, *J* = 2.6 Hz, 2H), 3.72 (s, 6H), 2.95 – 2.58 (m, 4H).

^{13}C NMR (101 MHz, $CDCl_3$): δ 158.3, 156.2, 130.8, 120.5, 113.1, 104.2, 55.5, 31.2.

ESI-MS: 269.09 $[M+H]^+$

HRMS: found 268.12063 $[M]^+$

calc. 268.12000

Purification: column chromatography (cyclohexane/EtOAc = 2/1)

Appearance: yellow solid

Yield: 16 mg, 20%. This product contained some photo-inactive impurities, see the spectra in the Supp. Info.

3,8-Bis(methoxymethoxy)-11,12-dihydrodibenzo[*c*,*g*]-1,2-diazocine 1l

2l (397 mg, 1.01 mmol) was reacted with $LiAlH_4$ in THF (2.40 M, 8.5 mL, 20 mmol).

^{1}H NMR (400 MHz, $CDCl_3$): δ 6.88 (d, *J* = 8.4 Hz, 2H), 6.70 (dd, *J* = 8.4, 2.5 Hz, 2H), 6.54 (d, *J* = 2.5 Hz, 2H), 5.07 (s, 4H), 3.42 (s, 6H), 2.96 – 2.80 (m, 2H), 2.77 – 2.62 (m, 2H).

^{13}C NMR (101 MHz, $CDCl_3$): δ 156.2, 156.0, 130.7, 121.7, 115.1, 107.0, 94.8, 56.2, 31.2.

ESI-MS: 329.08 $[M+H]^+$

351.02 $[M+Na]^+$

HRMS: found 329.14932 $[M+H]^+$

calc. 329.14958

Purification: gradient sublimation (150 °C, 10^{-5} mbar, 1.5 h)

Appearance: yellow solid

Yield: 40 mg, 12%

4,7-Difluoro-11,12-dihydrodibenzo[*c*,*g*]-1,2-diazocine 1m

For the ring closure of **2m** (0.30 g, 1.0 mmol), the reagent $LiAlH(OMe)_3$ was first formed from $LiAlH_4$ in THF (2.40 M, 8.1 mL, 19 mmol) and MeOH (2.4 mL, 58.4 mmol) at 0 °C.

^{1}H NMR (400 MHz, $CDCl_3$): δ 7.03 (td, *J* = 8.0, 5.4 Hz, 2H), 6.89 (t, *J* = 8.9 Hz, 2H), 6.77 (d, *J* = 7.6 Hz, 2H), 3.04 – 2.89 (m, 2H), 2.88 – 2.74 (m, 2H).

^{13}C NMR (101 MHz, $CDCl_3$): δ 152.2 (d, *J* = 249.2 Hz), 143.2 (d, *J* = 13.6 Hz), 131.2 (s), 128.7 (d, *J* = 7.9 Hz), 124.9 (d, *J* = 3.5 Hz), 114.5 (d, *J* = 19.2 Hz), 31.5 (s).

ESI-MS: 225.08 $[M-F]^+$

245.07 $[M+H]^+$

HRMS: found 245.08897 $[M+H]^+$

calc. 245.08848

Purification: column chromatography (*n*-hexane/EtOAc = 4/1)

Appearance: yellow solid

Yield: 42 mg, 17%

4,7-Dimethyl-11,12-dihydrodibenzo[*c*,*g*]-1,2-diazocine 1n

2n (0.10 g, 0.33 mmol) and $LiAlH_4$ in THF (2.40 M, 2.8 mL, 6.6 mmol) were reacted

^{1}H NMR (400 MHz, $CDCl_3$): δ 6.93 – 6.91 (m, 4H), 6.80 (t, *J* = 4.4 Hz, 2H), 2.97 – 2.90 (m, 2H), 2.76 – 2.70 (m, 2H), 2.16 (s, 6H).

^{13}C NMR (101 MHz, $CDCl_3$): δ 154.3, 128.9, 128.4, 127.9, 127.3, 127.2, 32.1, 17.1.

ESI-MS: 237.16 $[M+H]^+$

HRMS: found 237.13862 $[M+H]^+$

calc. 237.13863

Purification: gradient sublimation (125 °C, 10^{-5} mbar, 2 h)

Appearance: yellow solid, Yield: 5 mg, 6%

2. UV-Vis spectra and kinetics

2.1 UV-Vis spectra of substituted dibenzodiazocines 1b-n

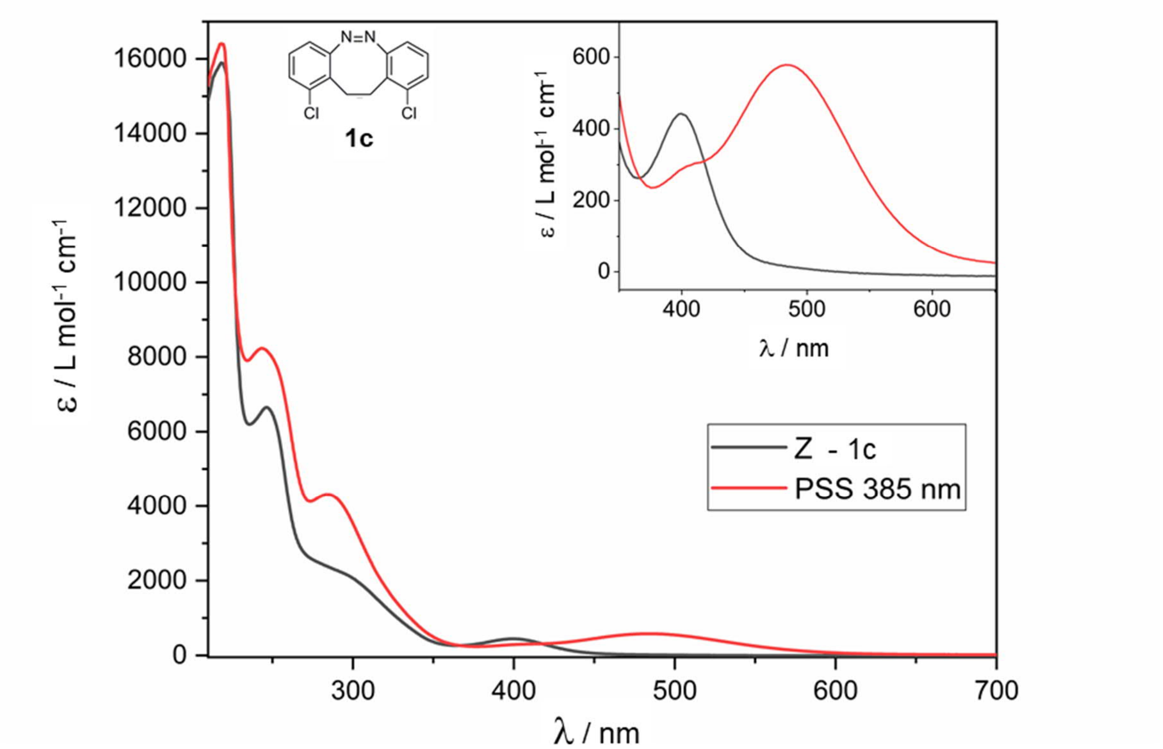

Figure S1. Absorption spectra of 1,10-dichloro-11,12-dihydrodibenzo[*c*,*g*]-1,2-diazocine **1c** in MeOH at ground state (Z-isomer; black) and at photo stationary state (PSS; red) at λ = 385 nm. Insert shows n→π* transitions.

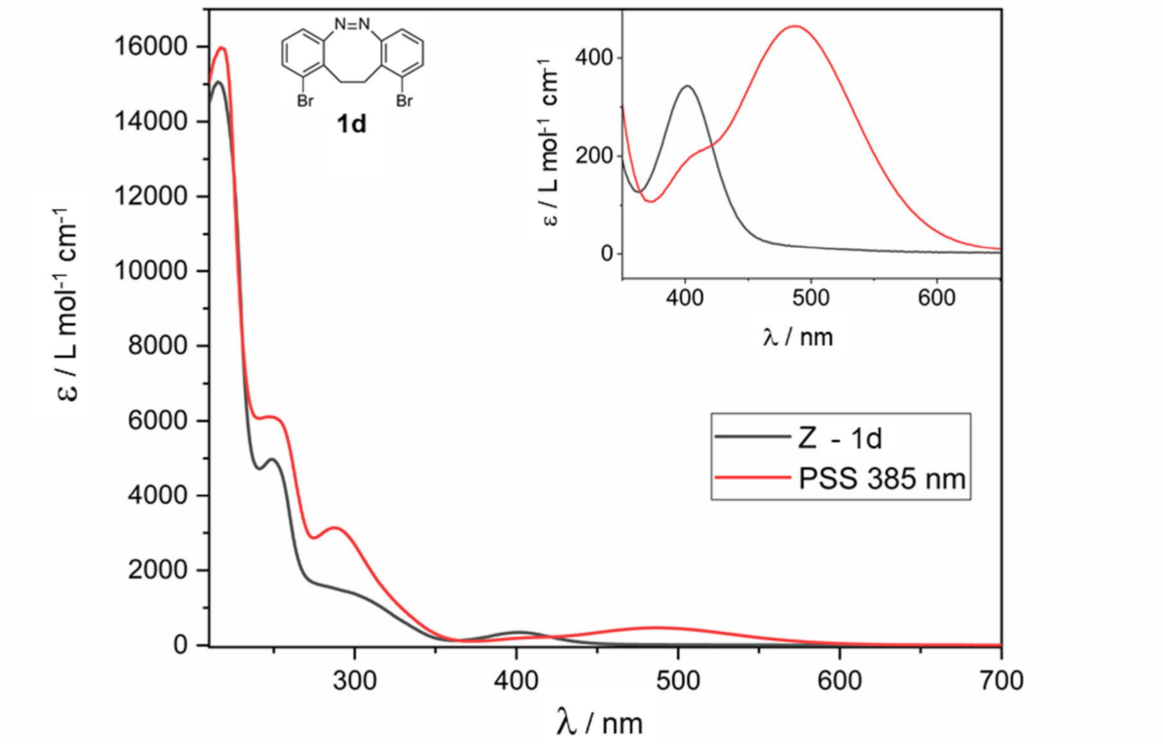

Figure S2. Absorption spectra of 1,10-dibromo-11,12-dihydrodibenzo[*c*,*g*]-1,2-diazocine **1d** in MeOH at ground state (*Z*-isomer; black) and at PSS (red) at λ = 385 nm. Insert shows n→π* transitions.

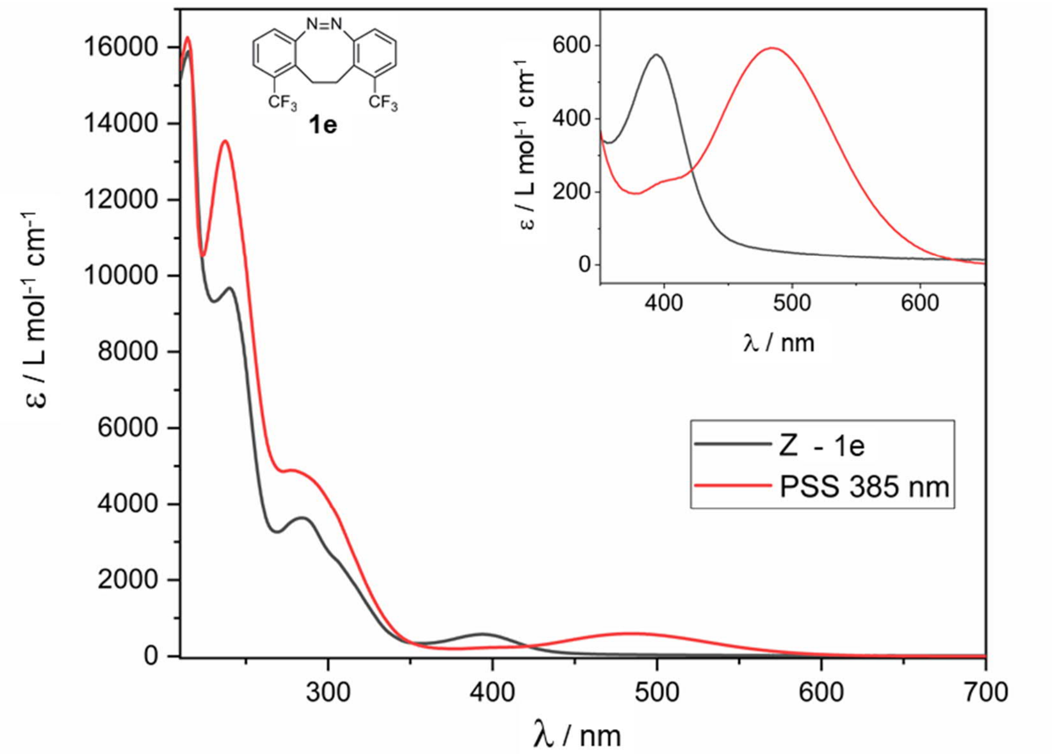

Figure S3. Absorption spectra of 1,10-bis(trifluoromethyl)-11,12-dihydrodibenzo[*c*,*g*]-1,2-diazocine **1e** in MeOH at ground state (*Z*-isomer; black) and at PSS (red) at λ = 385 nm. Insert shows n→π* transitions.

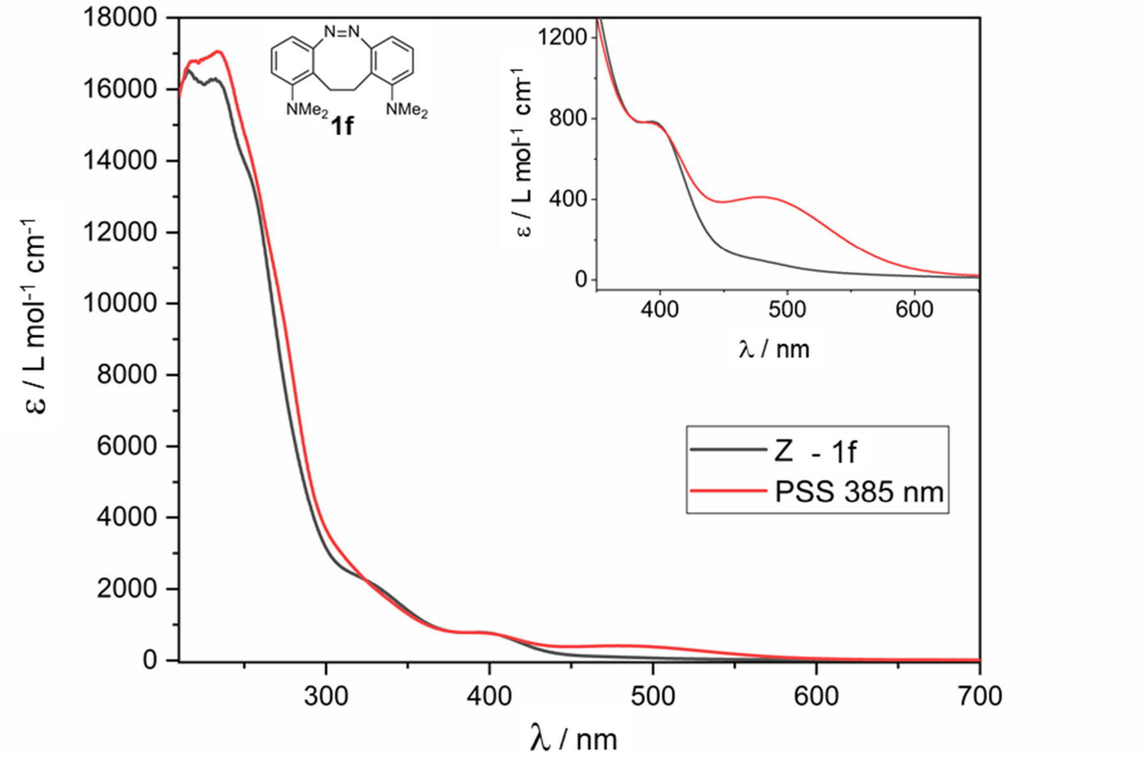

Figure S4. Absorption spectra of 2,9-bis(dimethylamino)-11,12-dihydrodibenzo[*c*,*g*]-1,2-diazocine **1f** in MeOH at ground state (*Z*-isomer; black) and at PSS (red) at λ = 385 nm. Insert shows n→π* transitions.

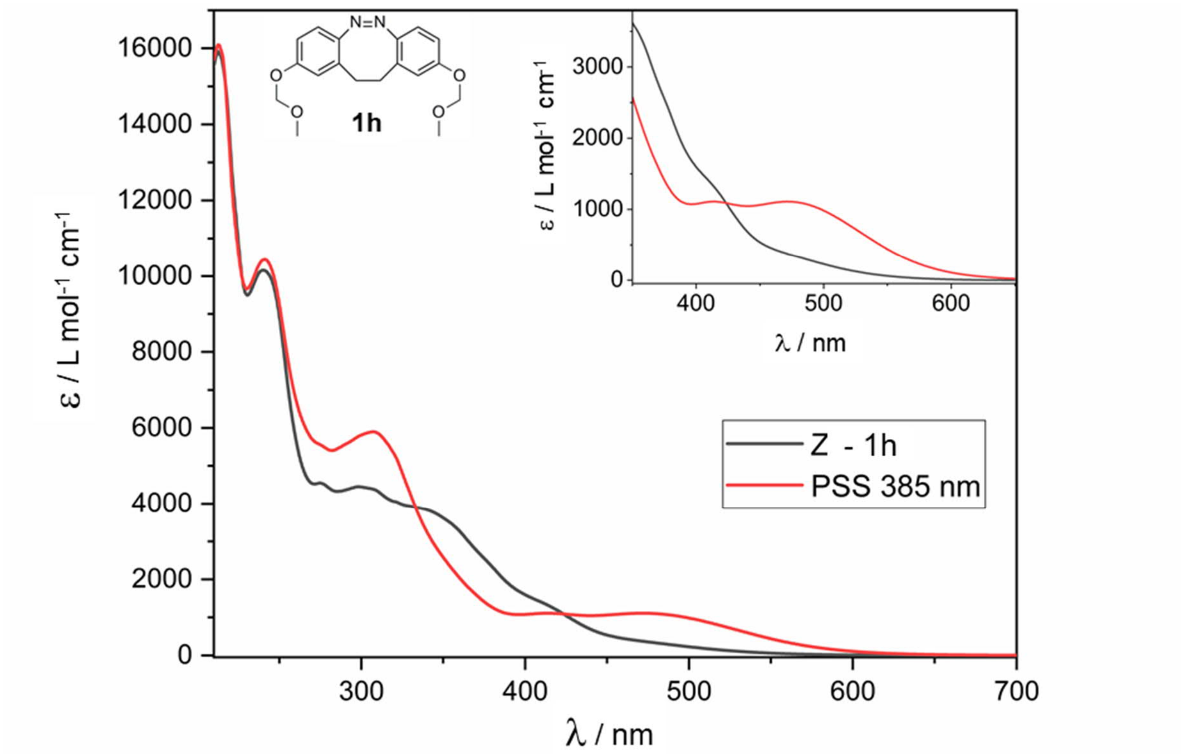

Figure S5. Absorption spectra of 2,9-bis(methoxymethoxy)-11,12-dihydrodibenzo[*c*,*g*]-1,2-diazocine **1h** in MeOH at ground state (*Z*-isomer; black) and at PSS (red) at λ = 385 nm. Insert shows n→π* transitions.

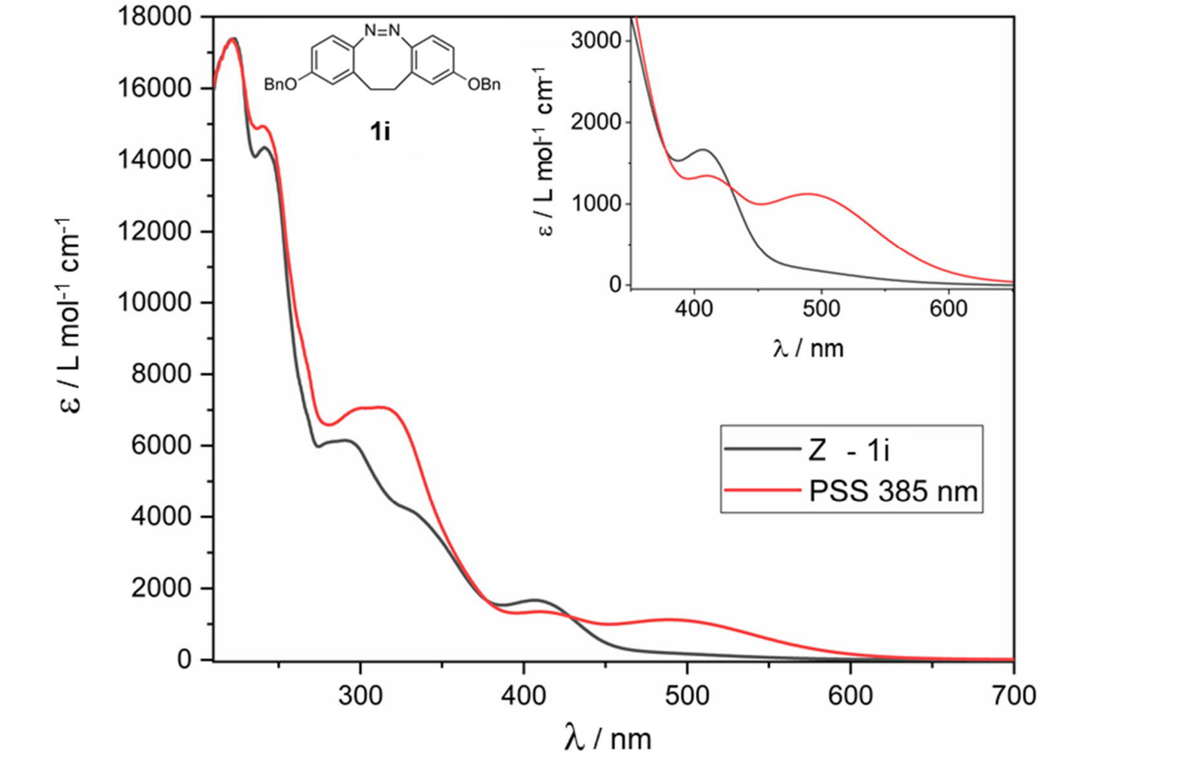

Figure S6. Absorption spectra of 2,9-dibenzyloxy-11,12-dihydrodibenzo[*c*,*g*]-1,2-diazocine **1i** in MeOH at ground state (*Z*-isomer; black) and at PSS (red) at λ = 385 nm. Insert shows n→π* transitions.

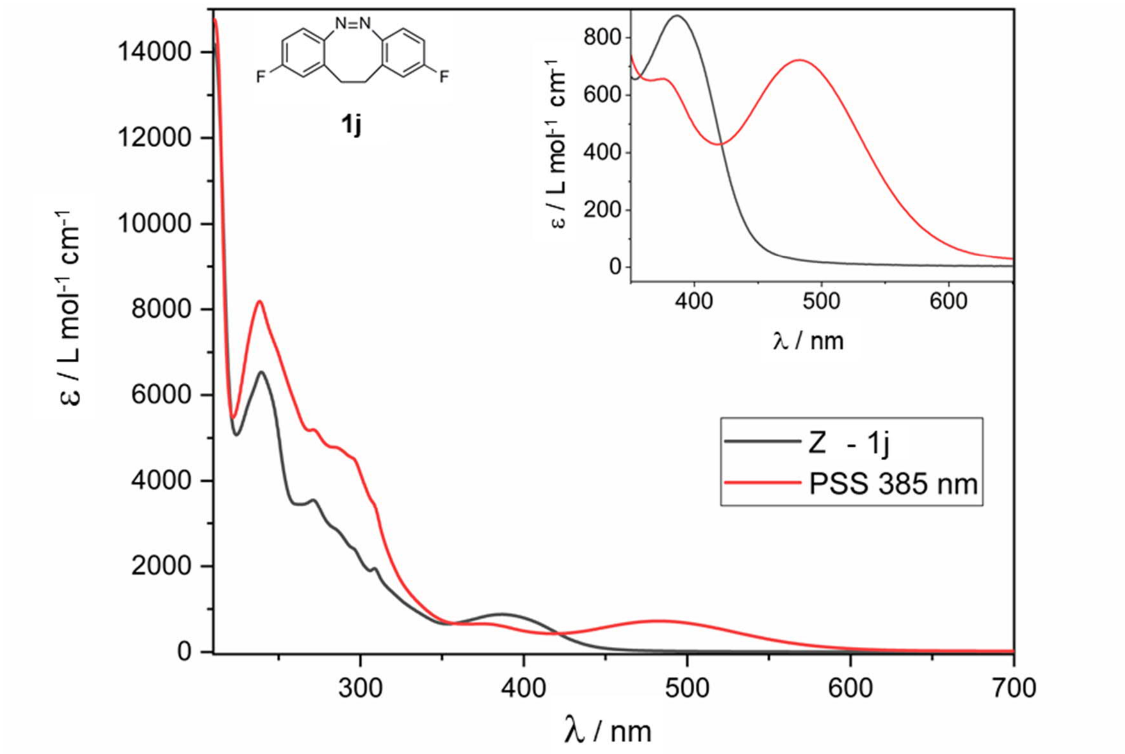

Figure S7. Absorption spectra of 2,9-difluoro-11,12-dihydrodibenzo[*c*,*g*]-1,2-diazocine **1j** in MeOH at ground state (*Z*-isomer; black) and at PSS (red) at λ = 385 nm. Insert shows n→π* transitions.

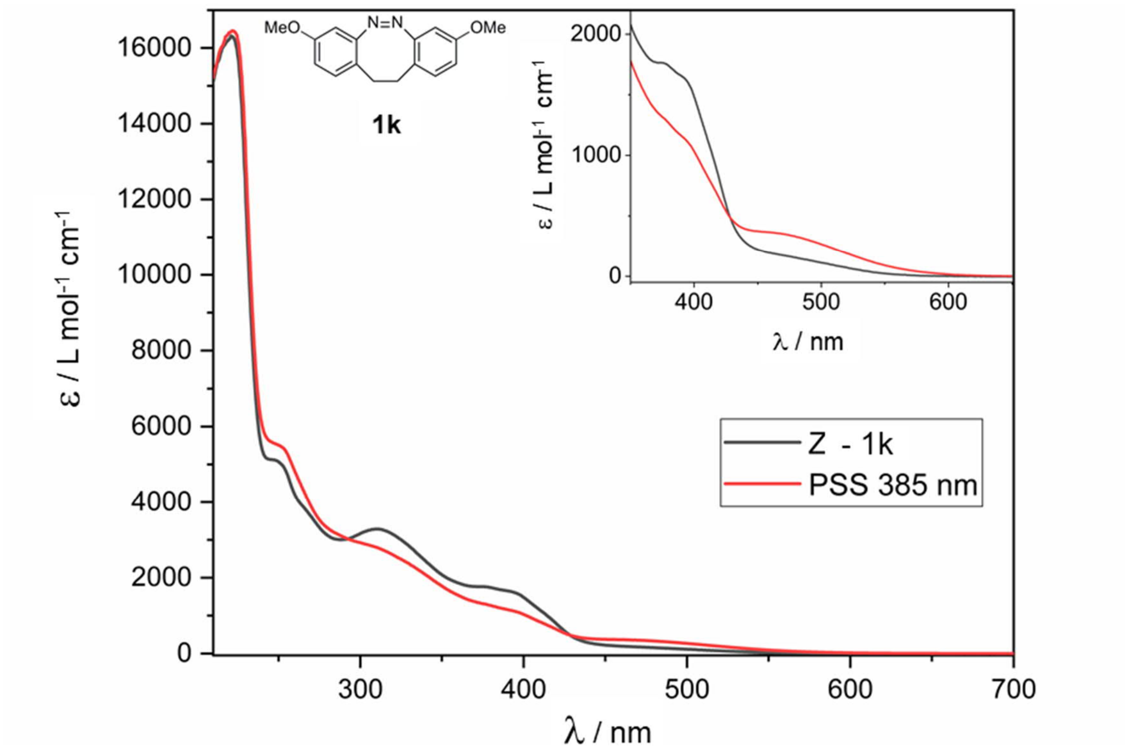

Figure S8. Absorption spectra of 3,8-dimethoxy-11,12-dihydrodibenzo[*c*,*g*]-1,2-diazocine **1k** in MeOH at ground state (*Z*-isomer; black) and at PSS (red) at λ = 385 nm. Insert show n→π* transitions.

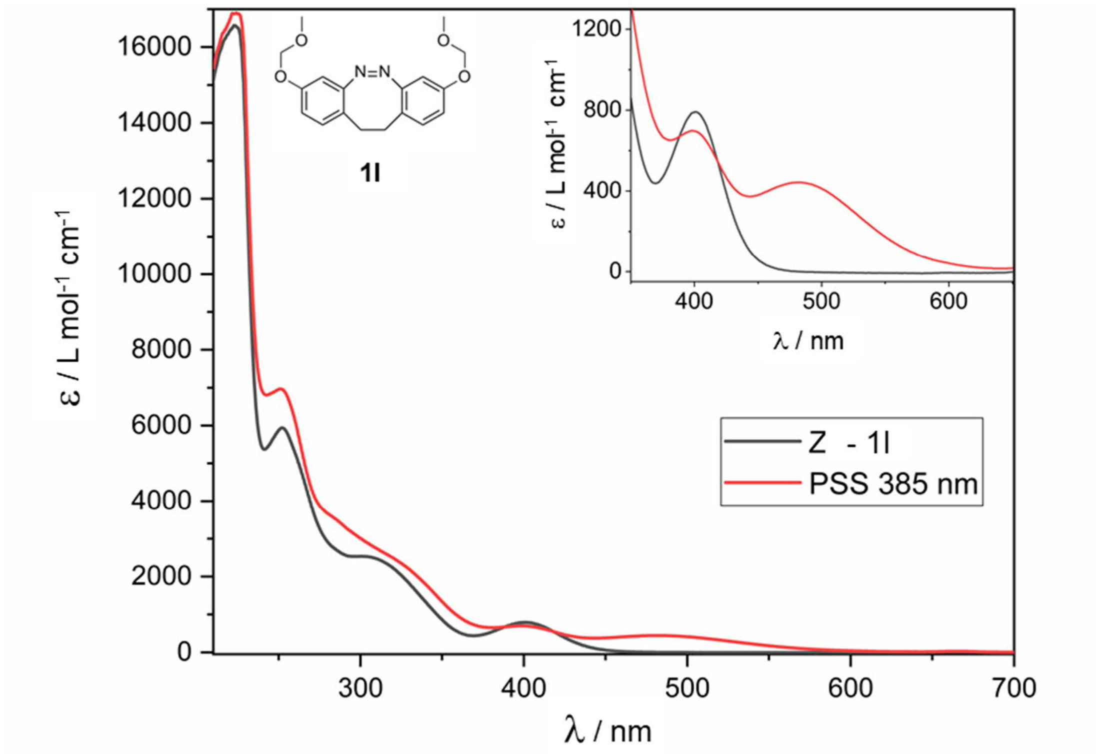

Figure S9. Absorption spectra of 3,8-bis(methoxymethoxy)-11,12-dihydrodibenzo[*c*,*g*]-1,2-diazocine **1l** in MeOH at ground state (*Z*-isomer; black) and at PSS (red) at λ = 385 nm. Insert show n→π* transitions.

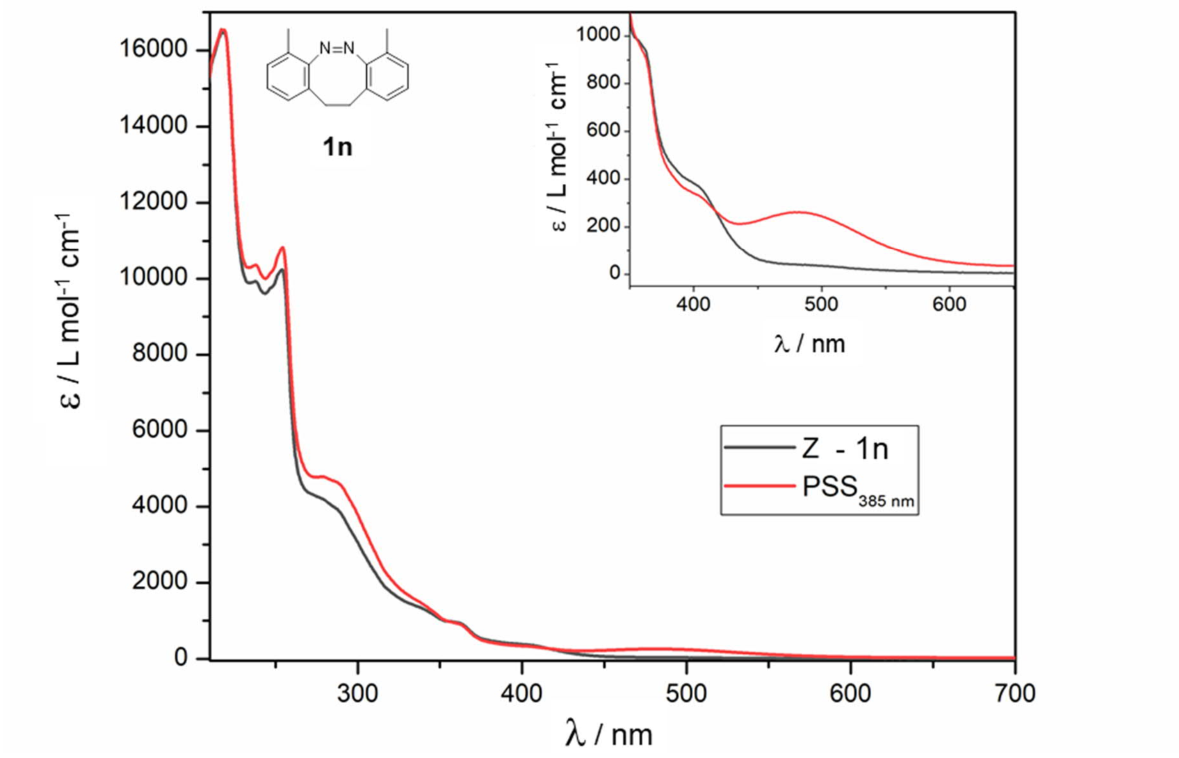

Figure S10. Absorption spectra of 4,10-dimethyl-11,12-dihydrodibenzo[*c*,*g*]-1,2-diazocine **1n** in MeOH at ground state (*Z*-isomer; black) and at PSS (red) at λ = 385 nm. Insert show n→π* transitions.

2.2 Determination of thermal half-life of the *E*-isomers

The thermal reisomerization reaction from the *E*- to the *Z*-isomer was assumed as a first order reaction:

$$ln\left(\frac{[E]_0}{[E]_t}\right) = kt \qquad (1)$$

with the reaction constant k and the concentration of the *E*-isomer [E].
In consideration of Beer's law, equation (1) can be converted to:

$$ln\left(\frac{A_\infty - A_0}{A_\infty - A_t}\right) = kt \qquad (2)$$

with the absorbance A of the *E*-isomer at λ = 480 nm.
The thermal half-life $\tau_{½}$ is given by:

$$\tau_{½} = \frac{\ln(2)}{k} \qquad (3)$$

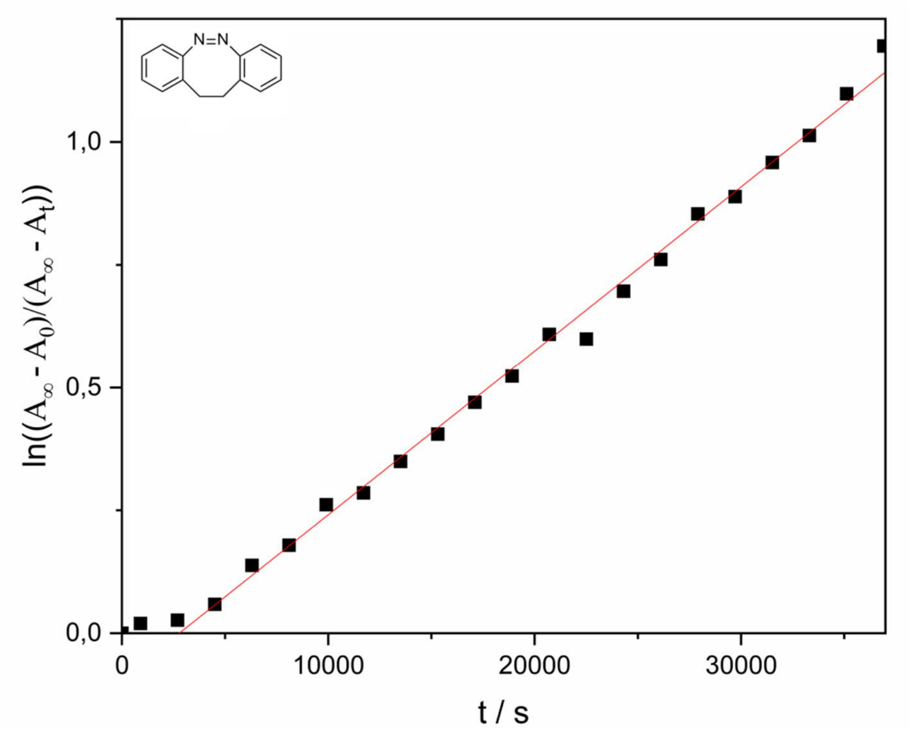

Figure S11. Logarithmized absorbance plotted as a function of time in seconds of 11,12-dihydrodibenzo[*c*,*g*]-1,2-diazocine **1a** in methanol. The slope of the linear fit (in red) gives the reaction constant k = (3.63 ± 0.05) × 10^{-5} s^{-1} ($\tau_{½}$ = 5.30 ± 0.07 h).

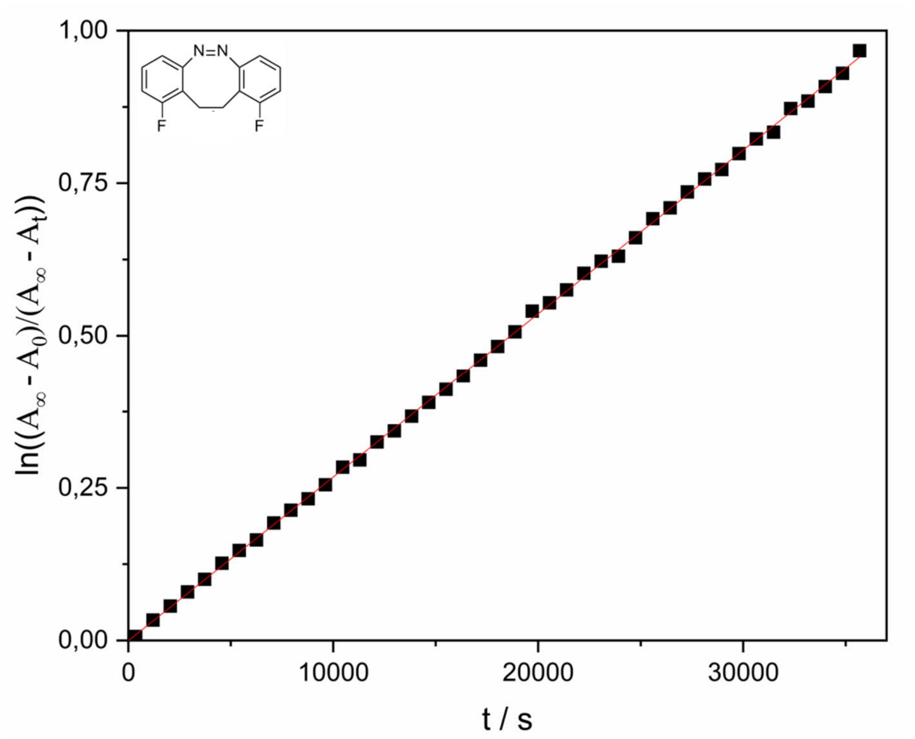

Figure S12. Logarithmized absorbance plotted as a function of time in seconds of 1,10-difluoro-11,12-dihydrodibenzo[*c*,*g*]-1,2-diazocine **1b** in methanol. The slope of the linear fit (in red) gives the reaction constant k = (2.682 ± 0.007) × 10^{-5} s^{-1} ($\tau_{½}$ = 7.18 ± 0.02 h).

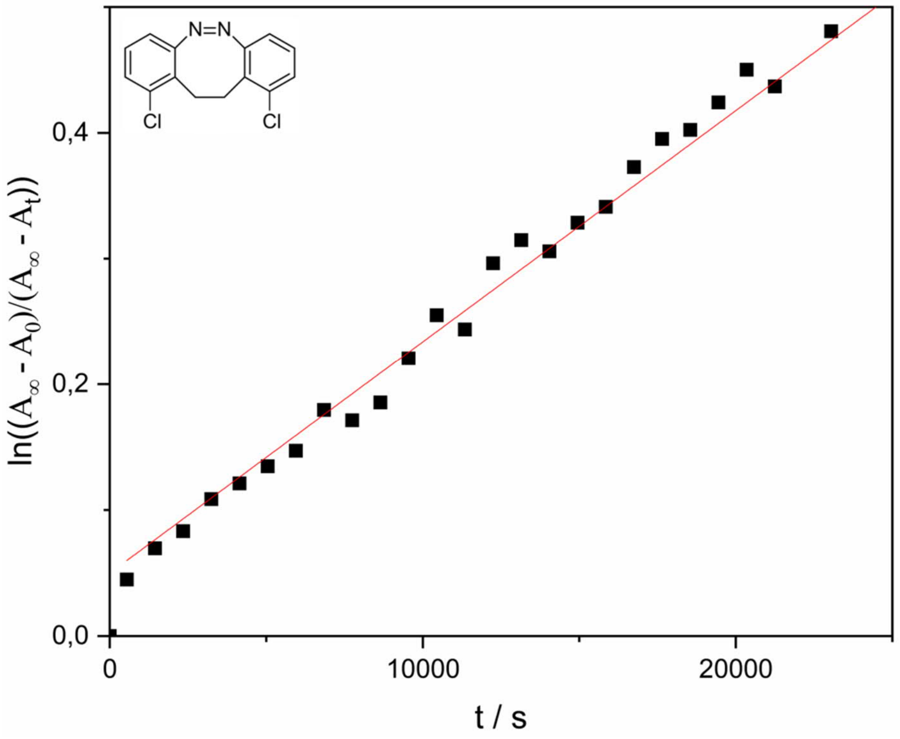

Figure S13. Logarithmized absorbance plotted as a function of time in seconds of 1,10-dichloro-11,12-dihydrodibenzo[*c*,*g*]-1,2-diazocine **1c** in methanol. The slope of the linear fit (in red) gives the reaction constant k = (1.84 ± 0.03) × 10^{-5} s^{-1} ($\tau_{½}$ = 10.5 ± 0.2 h).

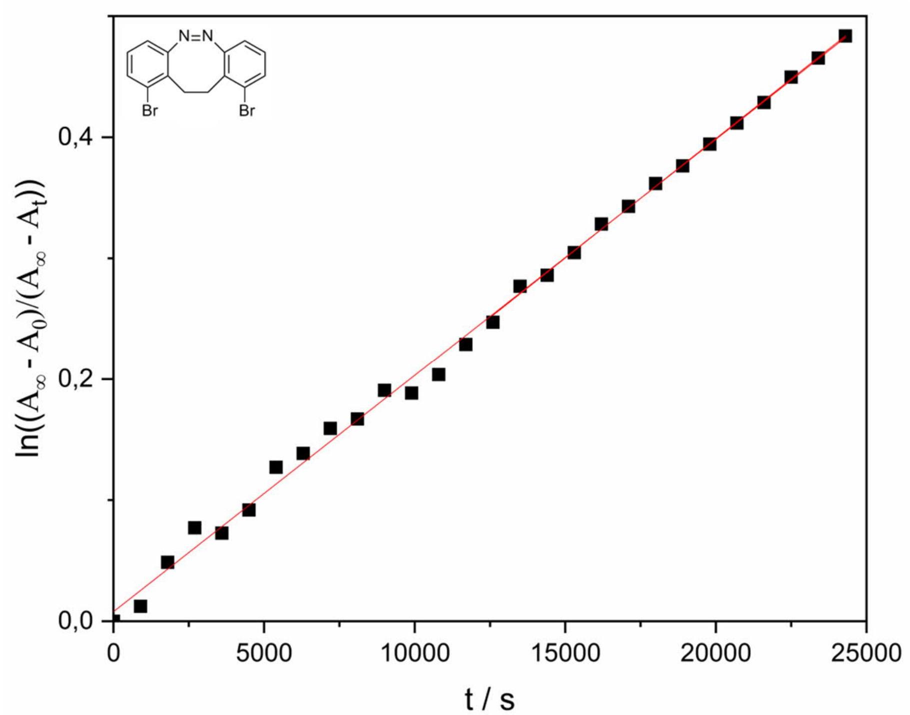

Figure S 14. Logarithmized absorbance plotted as a function of time in seconds of 1,10-dibromo-11,12-dihydrodibenzo[*c*,*g*]-1,2-diazocine **1d** in methanol. The slope of the linear fit (in red) gives the reaction constant k = (1.95 ± 0.02) × 10^{-5} s^{-1} ($\tau_{½}$ = 9.87 ± 0.10 h).

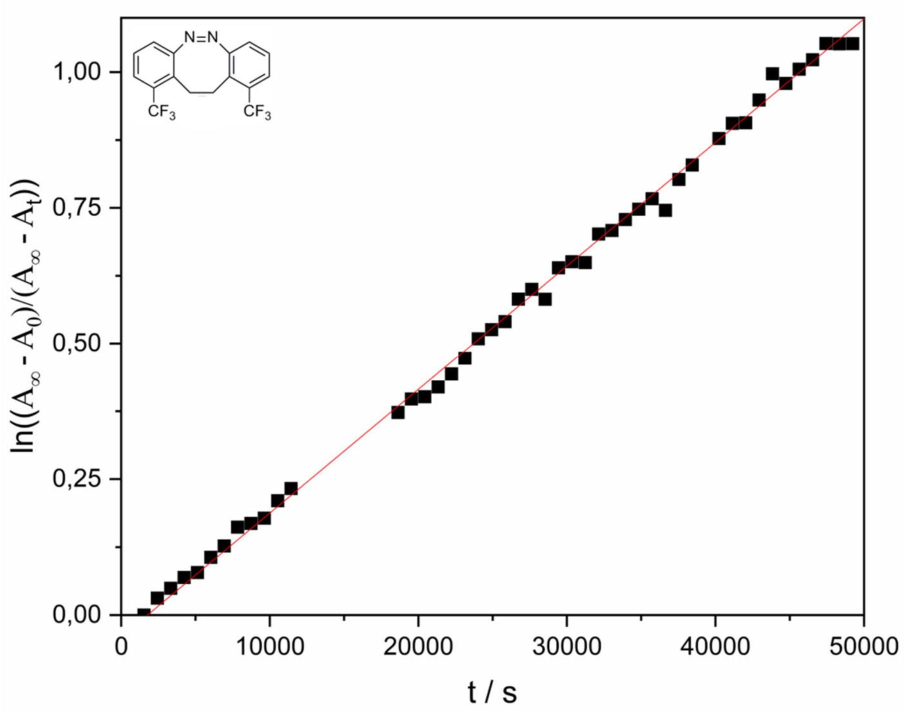

Figure S15. Logarithmized absorbance plotted as a function of time in seconds of 1,10-bis(trifluoromethyl)-11,12-dihydrodibenzo[*c*,*g*]-1,2-diazocine **1e** in methanol. The slope of the linear fit (in red) gives the reaction constant k = (2.277 ± 0.013) × 10^{-5} s^{-1} ($\tau_{½}$ = 8.46 ± 0.05 h).

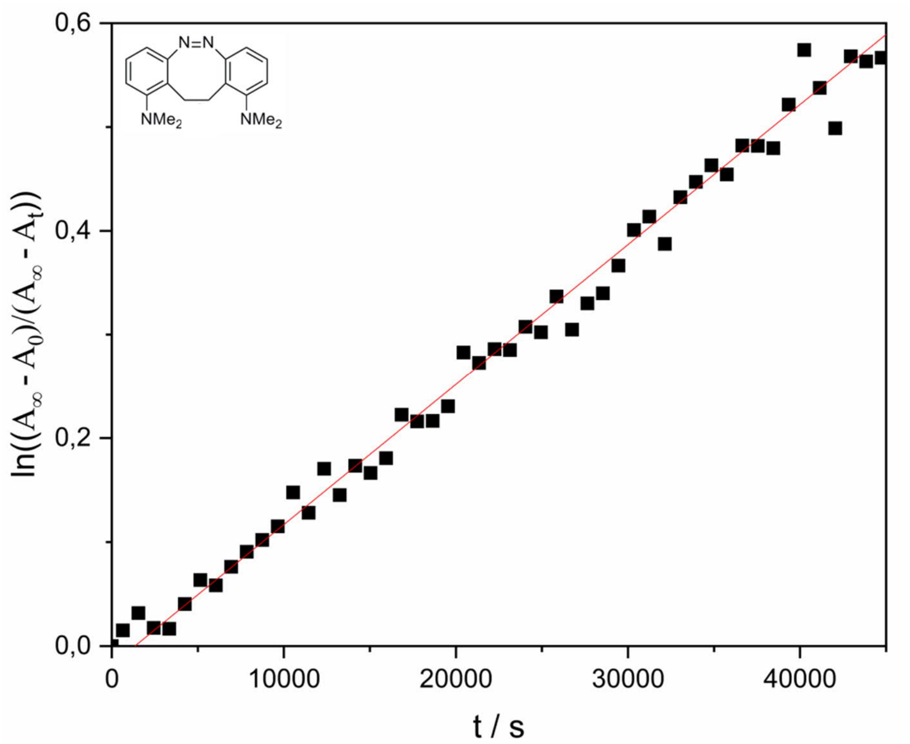

Figure S16. Logarithmized absorbance plotted as a function of time in seconds of 1,10-di(dimethylamino)-11,12-dihydrodibenzo[*c*,*g*]-1,2-diazocine **1f** in methanol. The slope of the linear fit (in red) gives the reaction constant k = (1.349 ± 0.015) × 10^{-5} s^{-1} ($\tau_{½}$ = 14.3 ± 0.2 h).

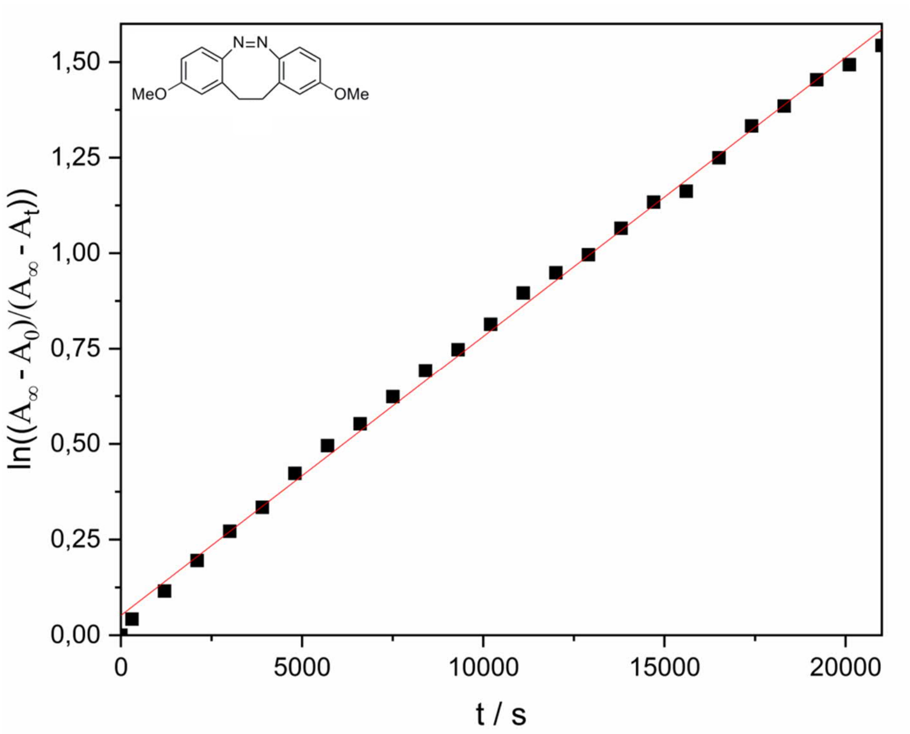

Figure S17. Logarithmized absorbance plotted as a function of time in seconds of 2,9-dimethoxy-11,12-dihydrodibenzo[*c*,*g*]-1,2-diazocine **1g** in methanol. The slope of the linear fit (in red) gives the reaction constant k = (7.30 ± 0.08) × 10^{-5} s^{-1} ($\tau_{½}$ = 2.64 ± 0.03 h).

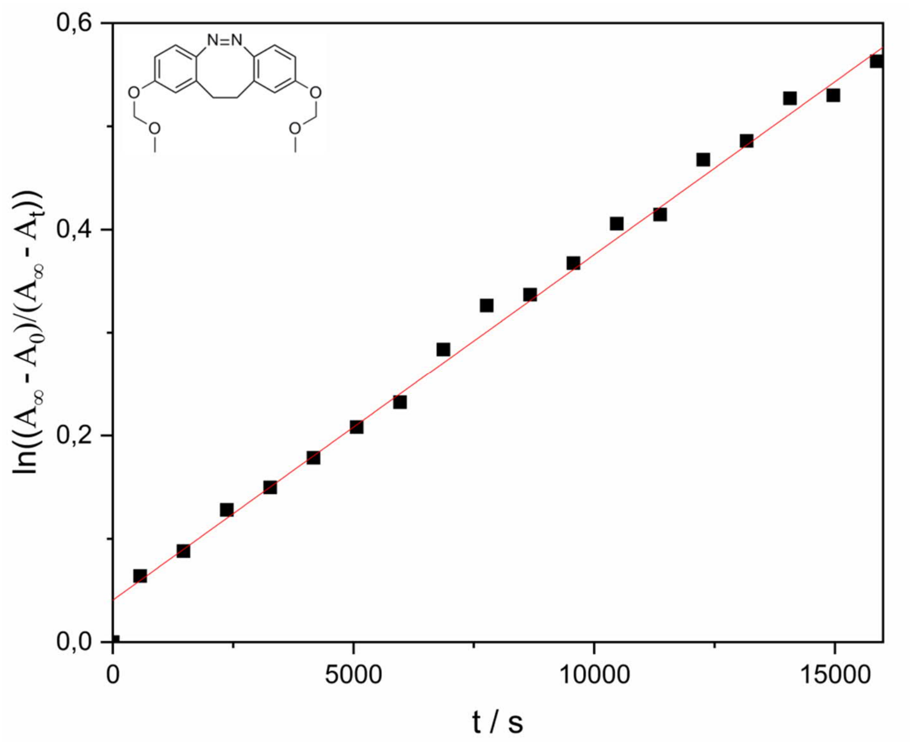

Figure S18. Logarithmized absorbance plotted as a function of time in seconds of 2,9-bis(methoxymethoxy)-11,12-dihydrodibenzo[*c*,*g*]-1,2-diazocine **1h** in methanol. The slope of the linear fit (in red) gives the reaction constant k = (8.70 ± 0.06) × 10^{-5} s^{-1} ($\tau_{½}$ = 2.21 ± 0.02 h).

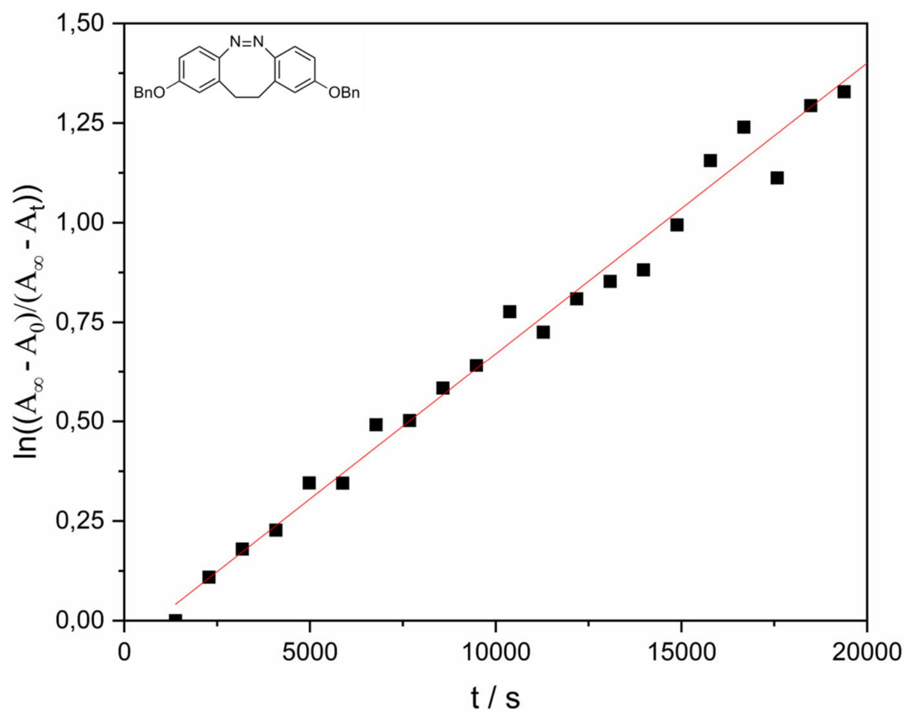

Figure S19. Logarithmized absorbance plotted as a function of time in seconds of 2,9-dibenzyloxy-11,12-dihydrodibenzo[*c*,*g*]-1,2-diazocine **1j** in methanol. The slope of the linear fit (in red) gives the reaction constant k = (7.30 ± 0.17) × 10^{-5} s^{-1} ($\tau_{½}$ = 2.64 ± 0.06 h).

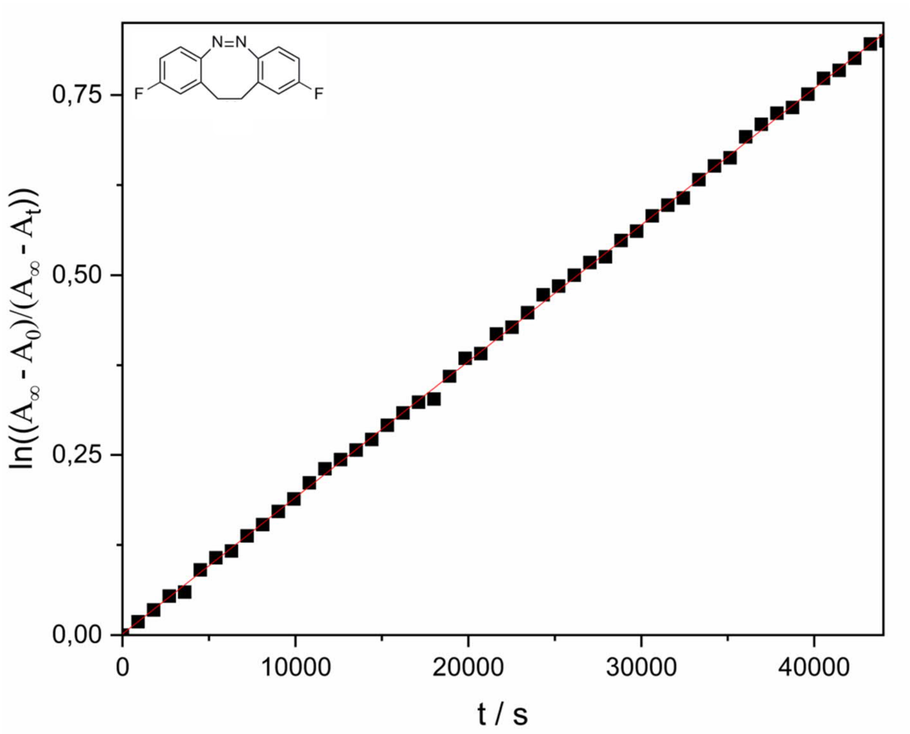

Figure S20. Logarithmized absorbance plotted as a function of time in seconds of 2,9-difluoro-11,12-dihydrodibenzo[*c*,*g*]-1,2-diazocine **1k** in methanol. The slope of the linear fit (in red) gives the reaction constant k = (1.894 ± 0.006) × 10^{-5} s^{-1} ($\tau_{½}$ = 10.2 ± 0.1 h).

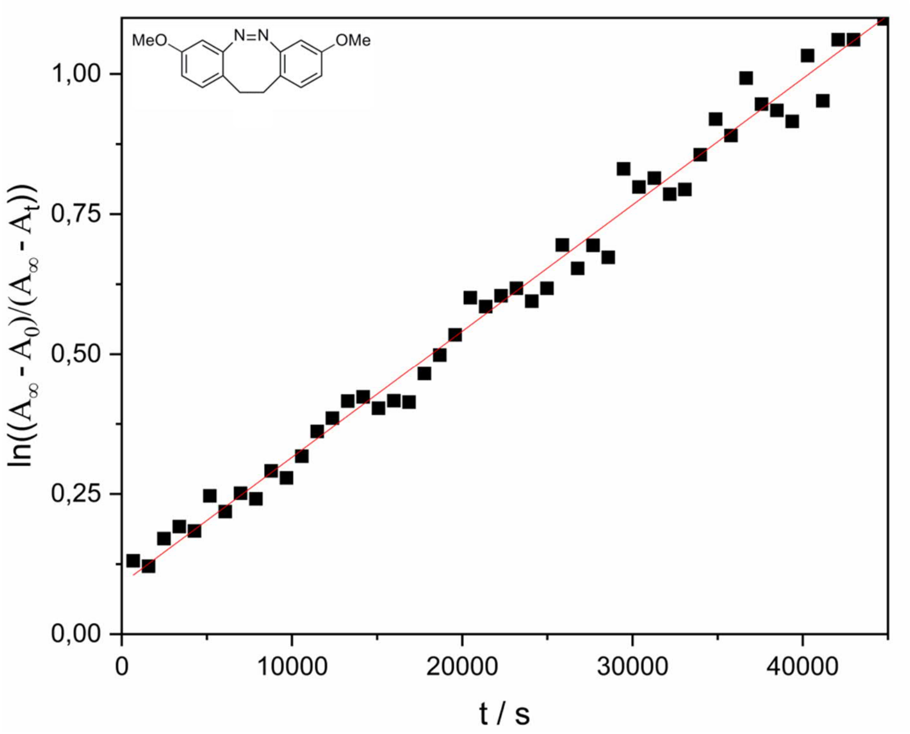

Figure S21. Logarithmized absorbance plotted as a function of time in seconds of 3,8-dimethoxy-11,12-dihydrodibenzo[*c*,*g*]-1,2-diazocine **1k** in methanol. The slope of the linear fit (in red) gives the reaction constant k = (2.25 ± 0.04) × 10^{-5} s^{-1} ($\tau_{½}$ = 8.56 ± 0.15 h).

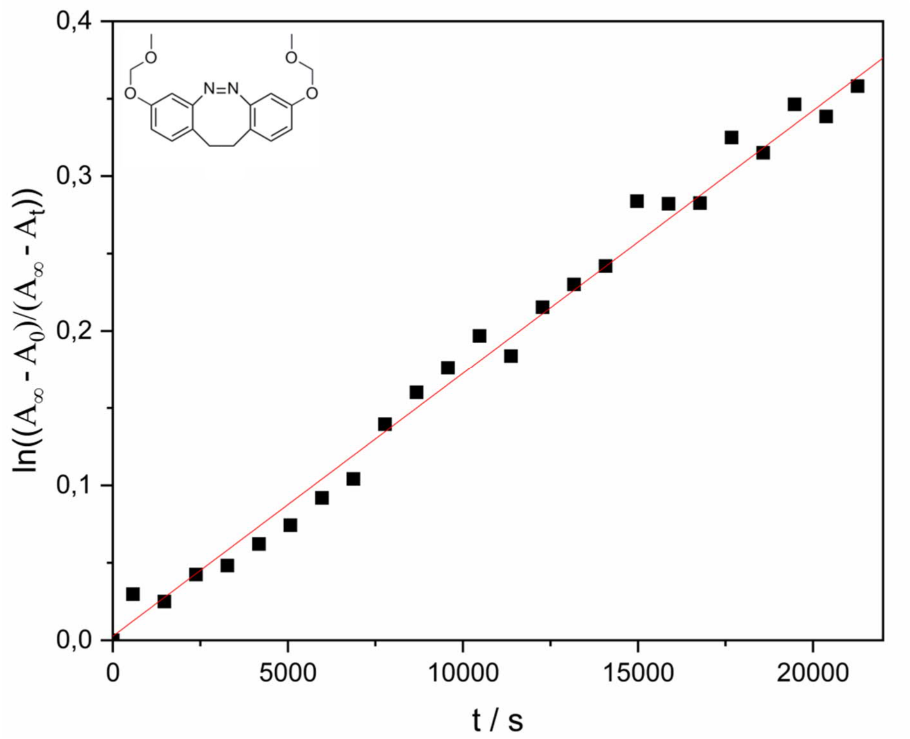

Figure S22. Logarithmized absorbance plotted as a function of time in seconds of 3,8-bis(methoxymethoxy)-11,12-dihydrodibenzo[*c*,*g*]-1,2-diazocine **1l** in methanol. The slope of the linear fit (in red) gives the reaction constant k = (1.70 ± 0.02) × 10^{-5} s^{-1} ($\tau_{½}$ = 11.3 ± 0.2 h).

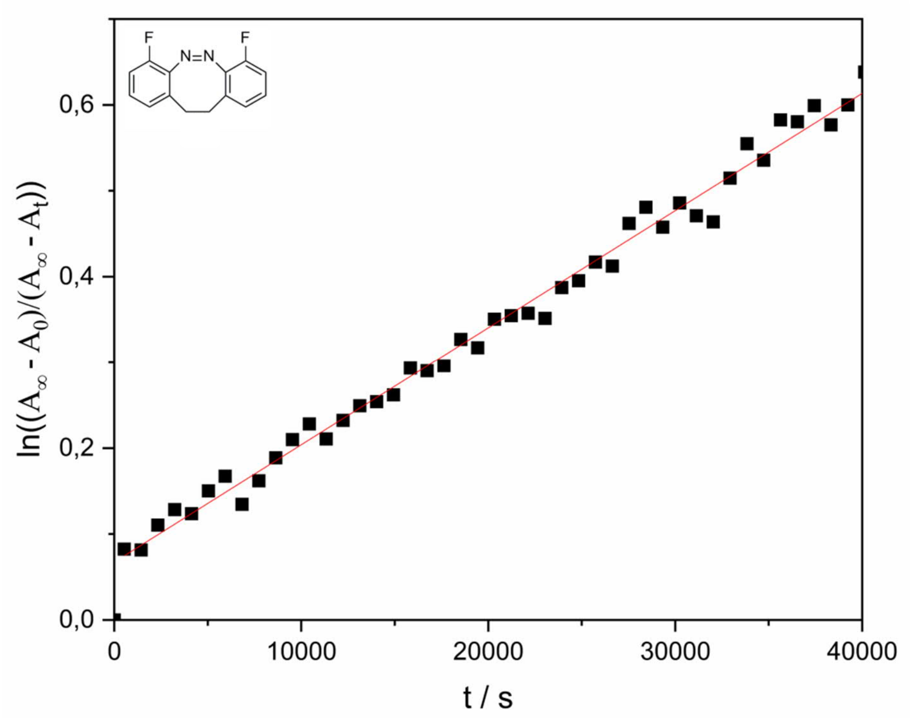

Figure S23. Logarithmized absorbance plotted as a function of time in seconds of 4,7-difluoro-11,12-dihydrodibenzo[*c*,*g*]-1,2-diazocine **1m** in methanol. The slope of the linear fit (in red) gives the reaction constant k = (1.37 ± 0.02) × 10^{-5} s^{-1} ($\tau_{½}$ = 14.1 ± 0.2 h).

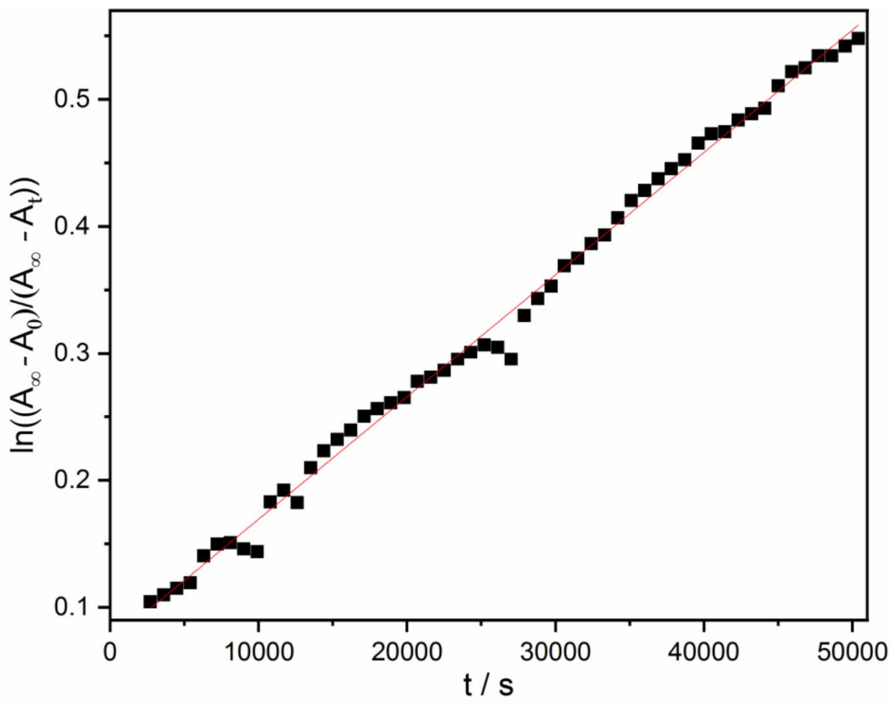

Figure S24. Logarithmized absorbance plotted as a function of time in seconds of 4,7-dimethyl-11,12-dihydrodibenzo[*c*,*g*]-1,2-diazocine **1n** in methanol. The slope of the linear fit (in red) gives the reaction constant k = (9.63 ± 0.09) × 10^{-6} s^{-1} ($\tau_{½}$ = 20.0 ± 0.2 h).

3. NMR spectra of synthesized compounds

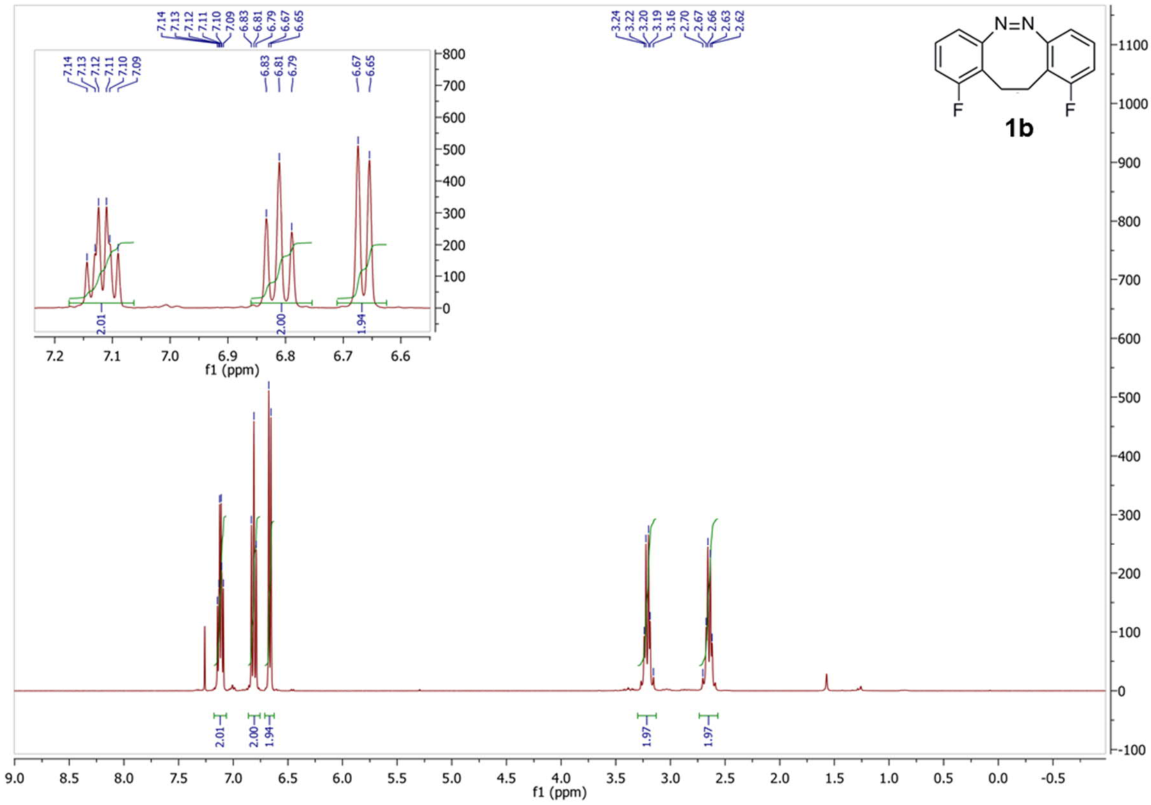

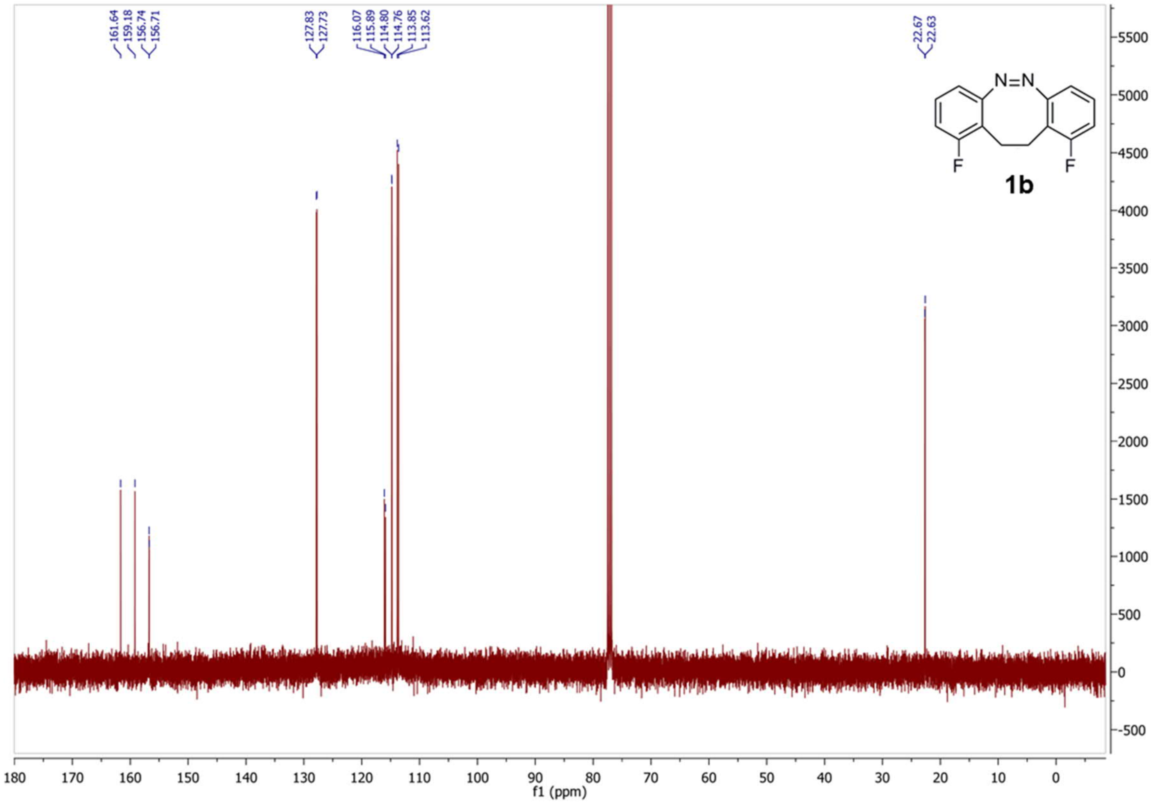

Figure S25. ^{1}H NMR (400 MHz) and ^{13}C NMR (101 MHz) spectra of 1,10-difluoro-11,12-dihydrodibenzo[*c*,*g*]-1,2-diazocine **1b** in $CDCl_3$.

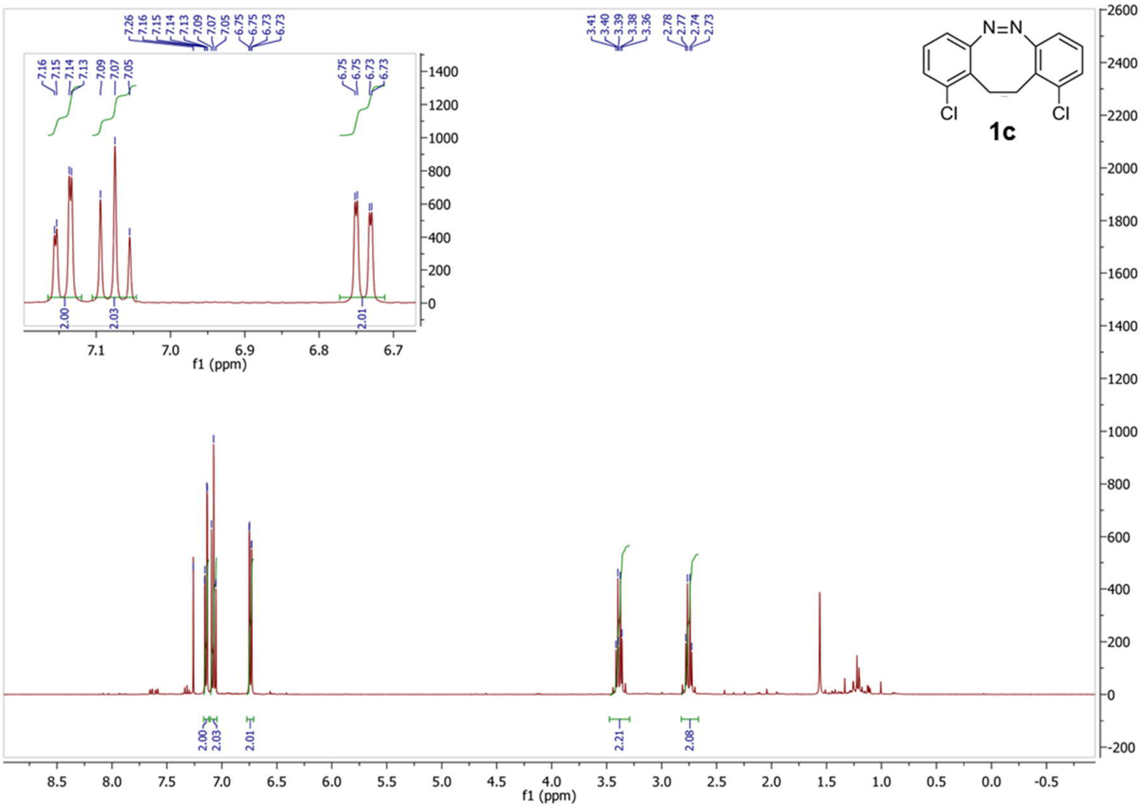

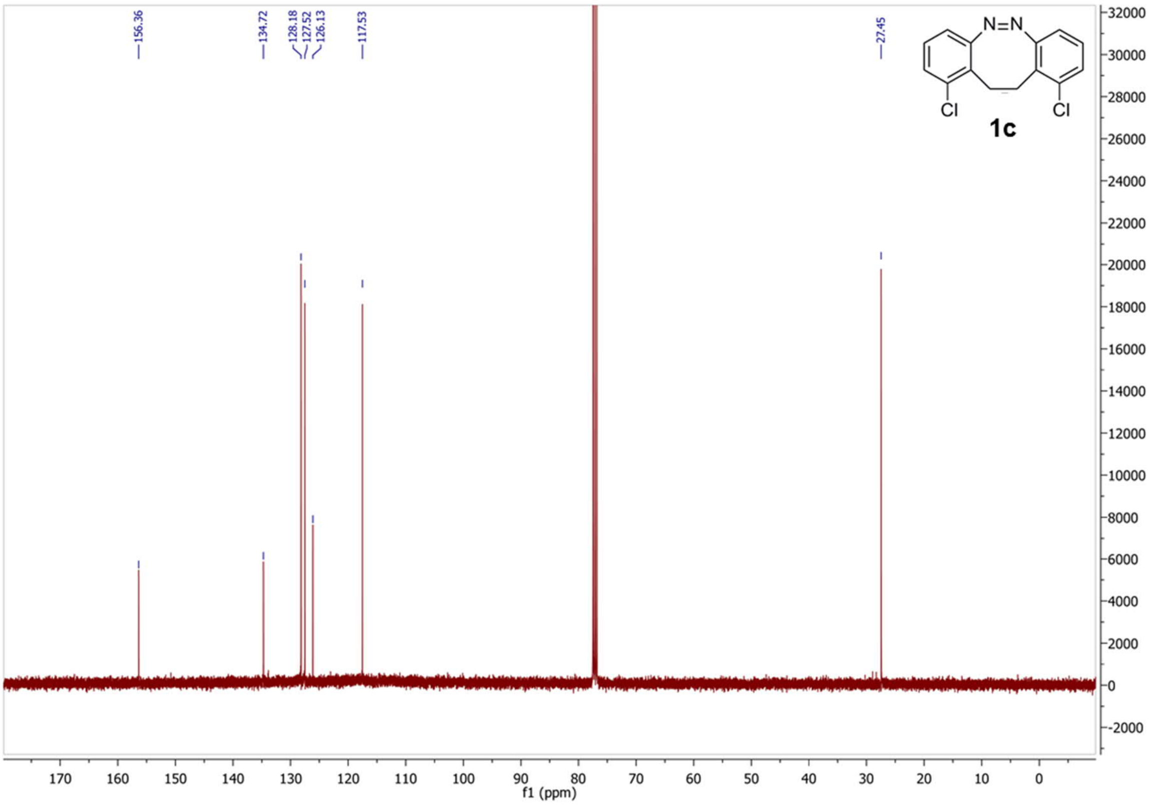

Figure S26. ^{1}H NMR (400 MHz) and ^{13}C NMR (101 MHz) spectra of 1,10-dichloro-11,12-dihydrodibenzo[*c*,*g*]-1,2-diazocine **1c** in $CDCl_3$.

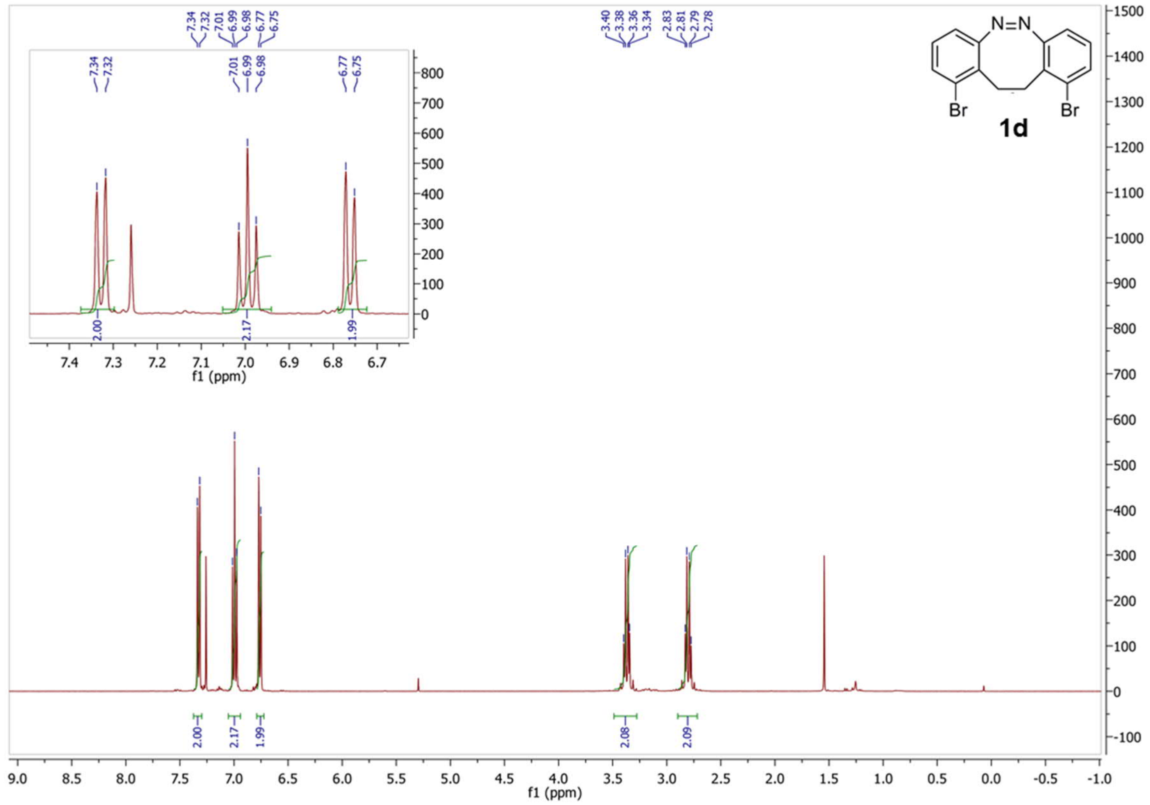

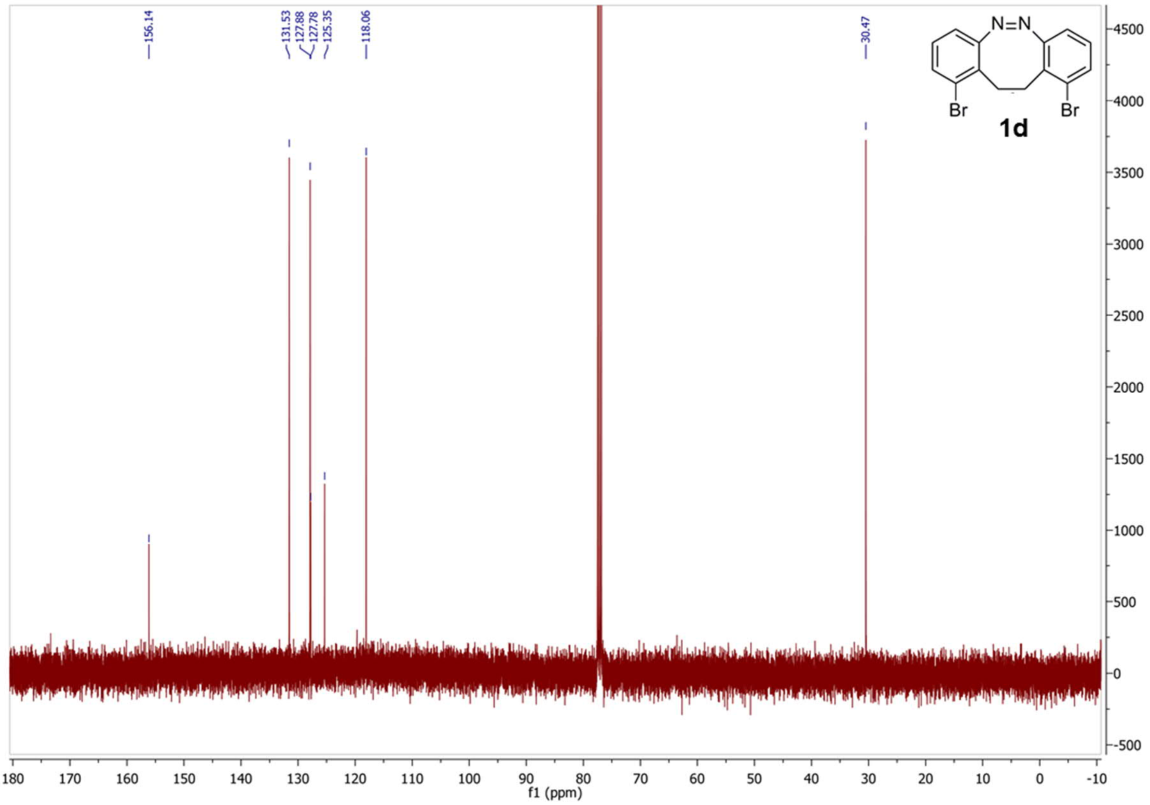

Figure S27. ^{1}H NMR (400 MHz) and ^{13}C NMR (101 MHz) spectra of 1,10-dibromo-11,12-dihydrodibenzo[*c*,*g*]-1,2-diazocine **1d** in $CDCl_3$.

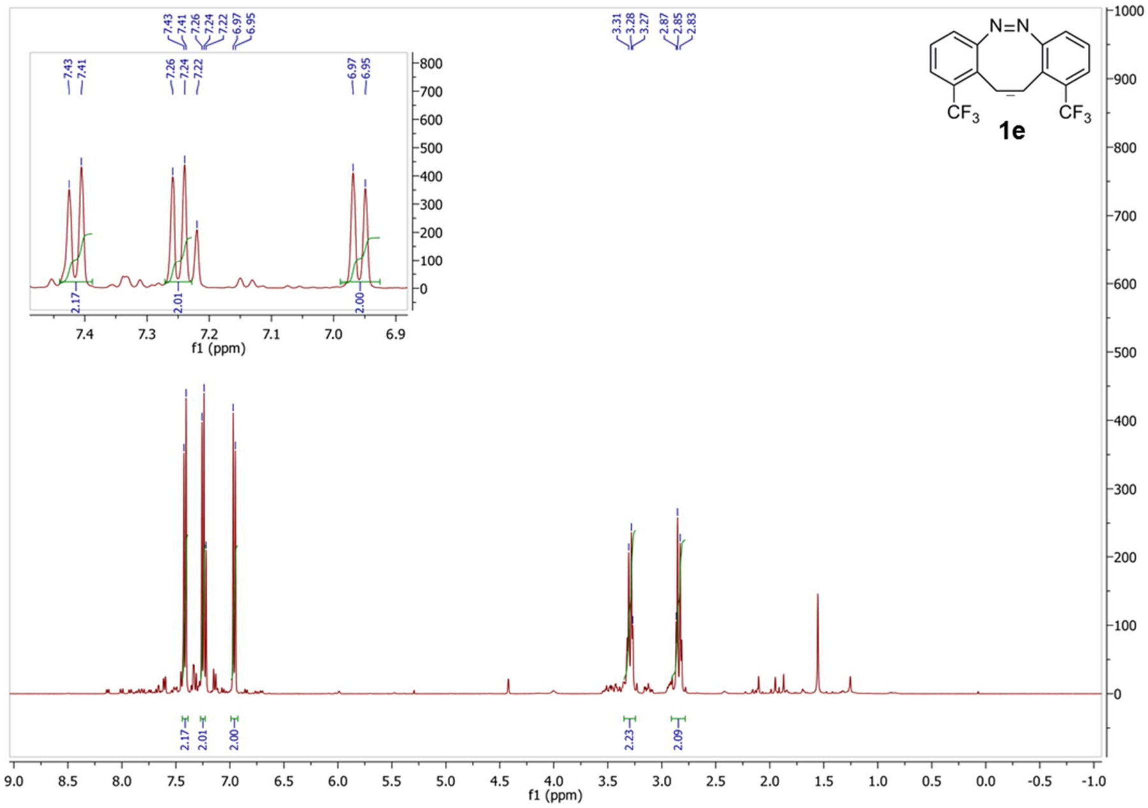

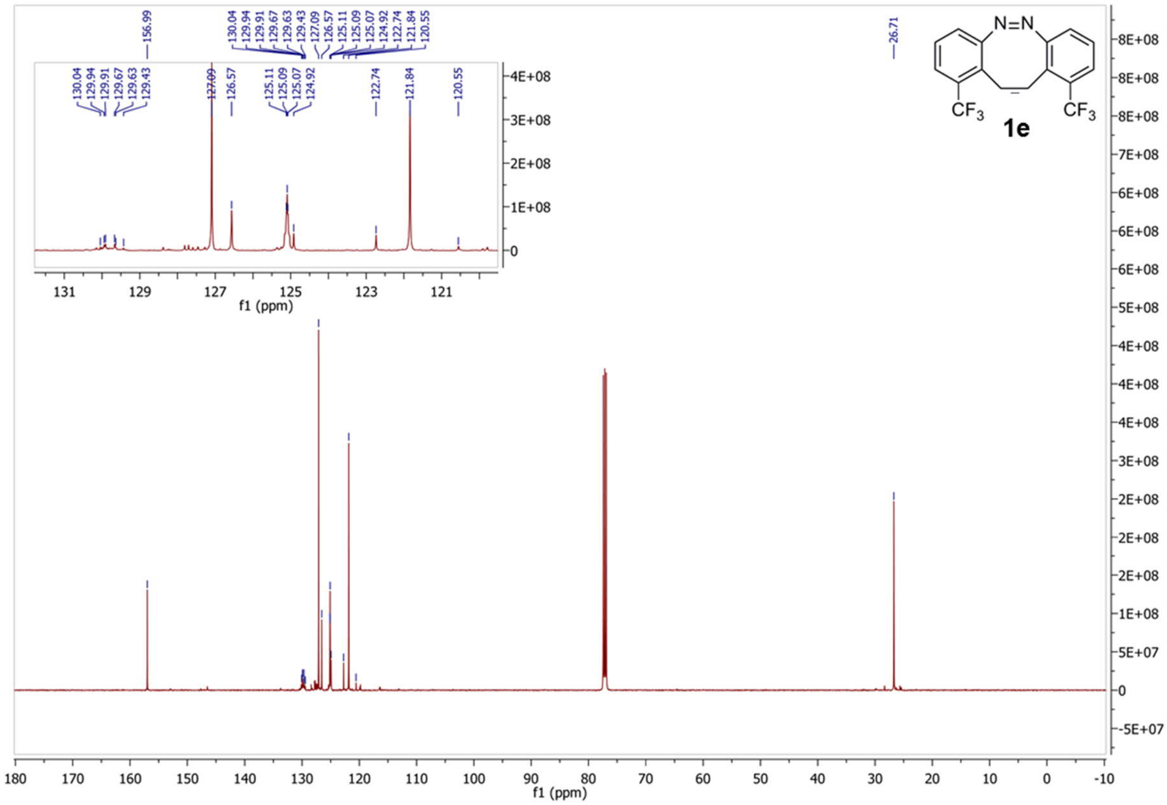

Figure S28. ^{1}H NMR (400 MHz) and ^{13}C NMR (126 MHz) spectra of 1,10-bis(trifluoromethyl)-11,12-dihydrodibenzo[*c*,*g*]-1,2-diazocine **1e** in $CDCl_3$.

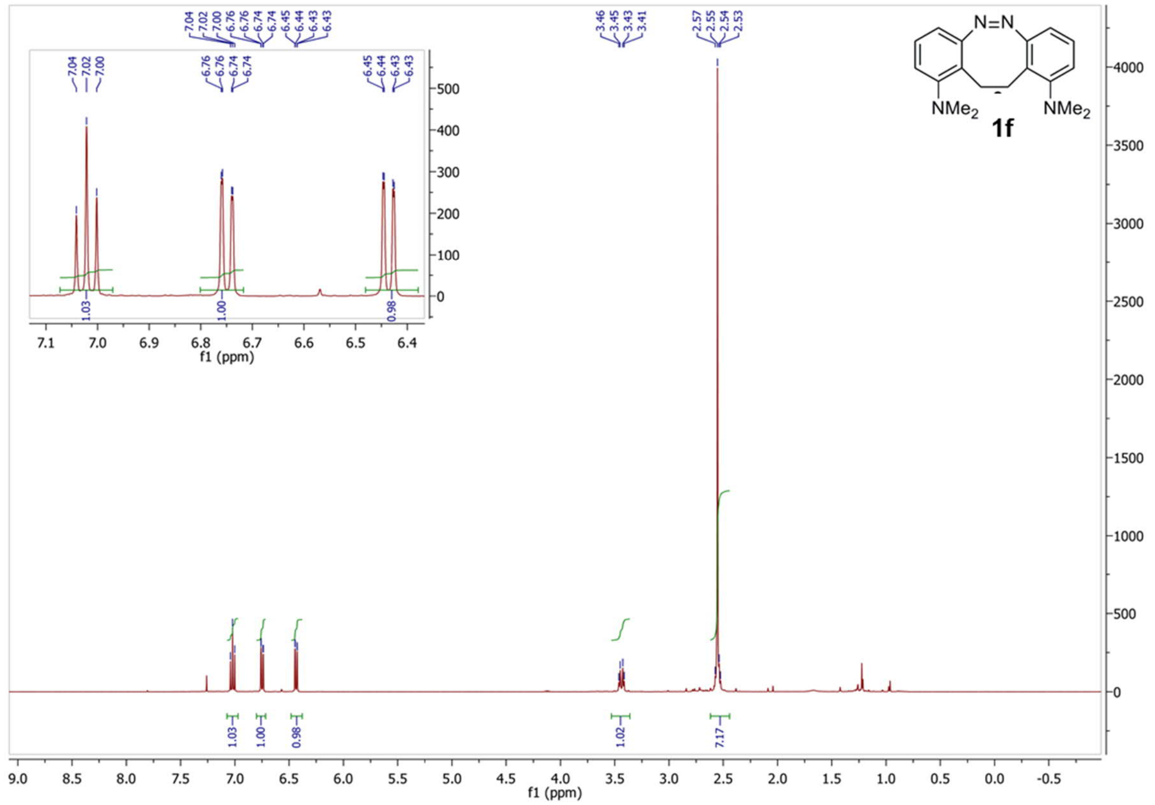

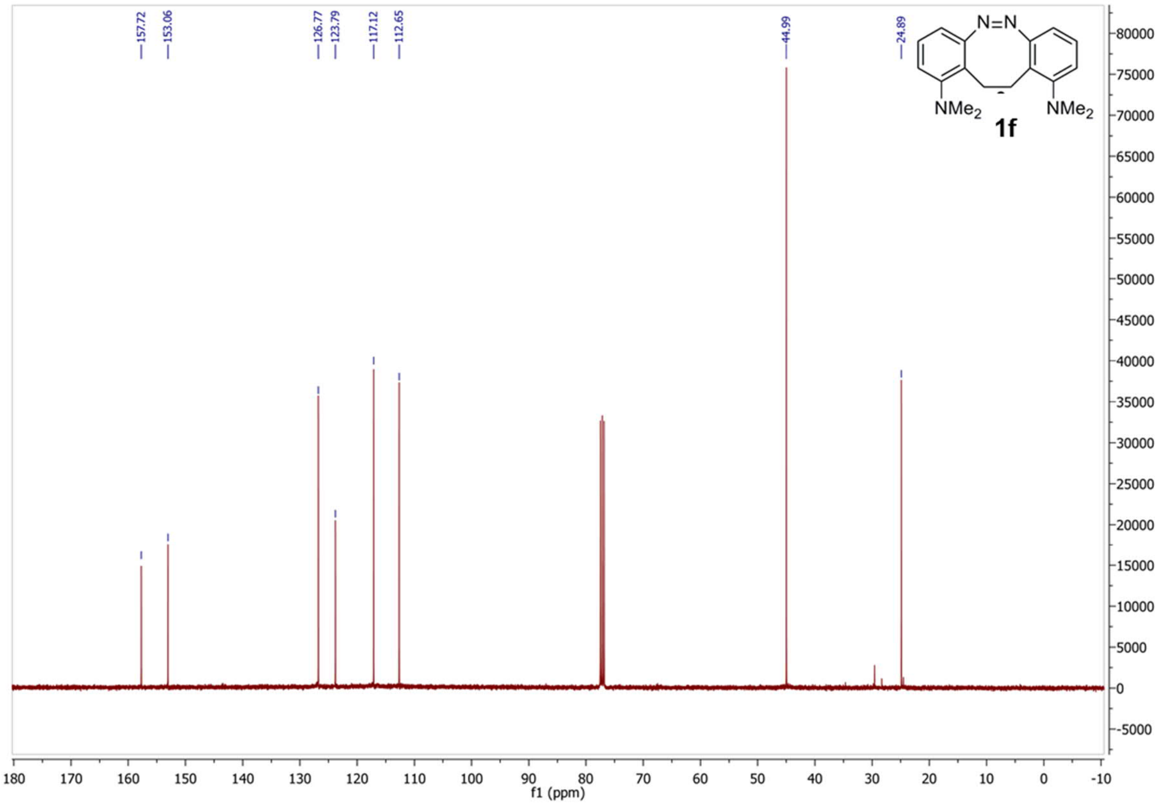

Figure S29. ^{1}H NMR (400 MHz) and ^{13}C NMR (101 MHz) spectra of 1,10-bis(dimethylamino)-11,12-dihydrodibenzo[*c*,*g*]-1,2-diazocine **1f** in $CDCl_3$.

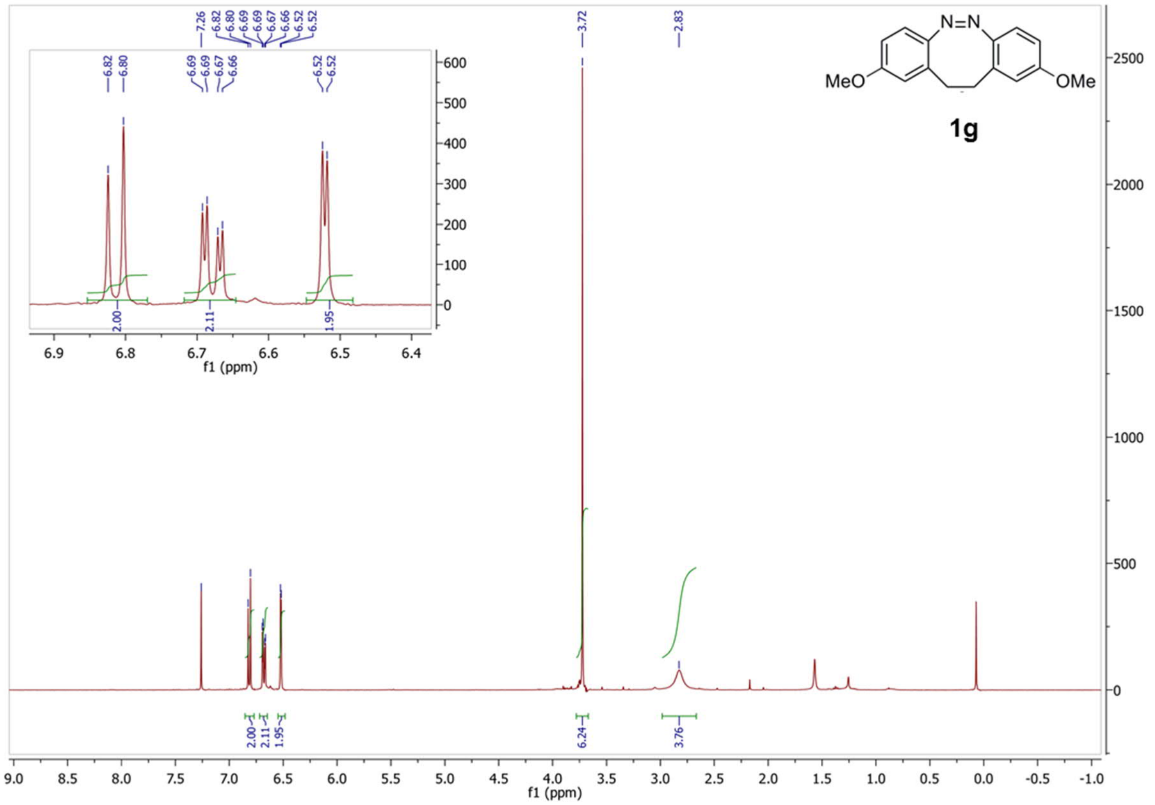

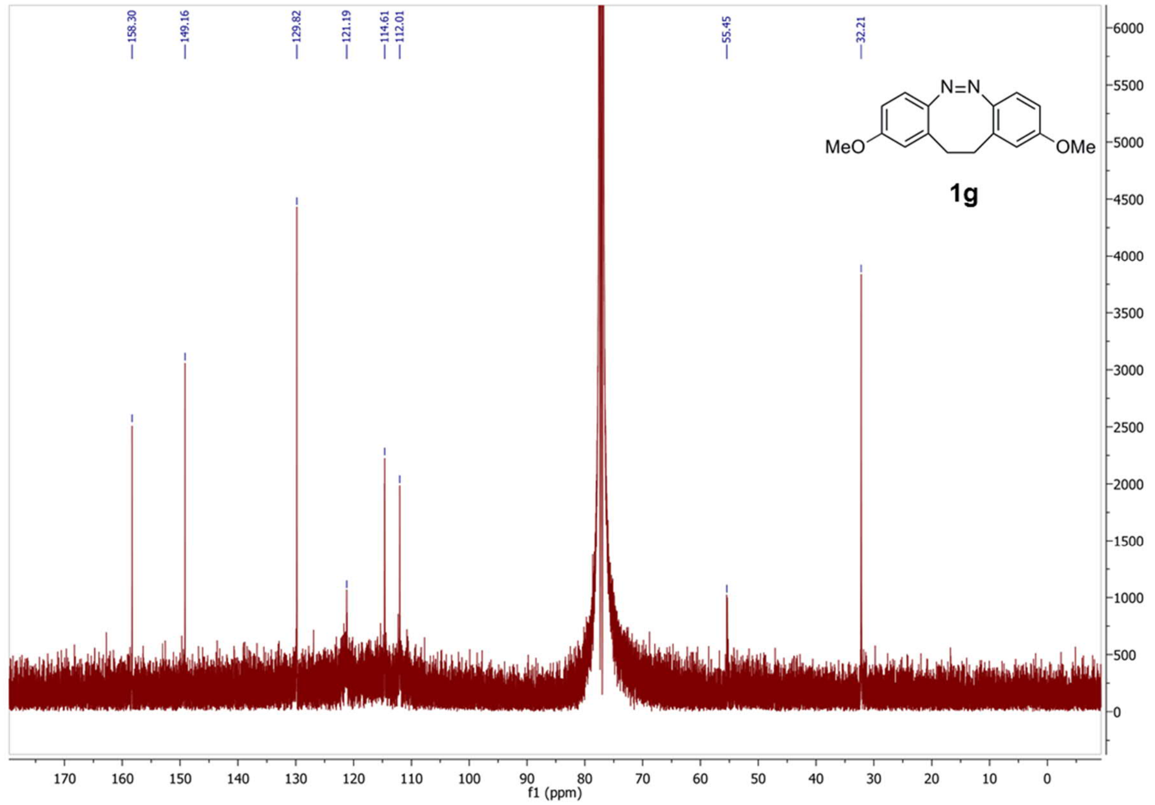

Figure S30. ^{1}H NMR (400 MHz) and ^{13}C NMR (101 MHz) spectra of 2,9-dimethoxy-11,12-dihydrodibenzo[*c*,*g*]-1,2-diazocine **1g** in $CDCl_3$.

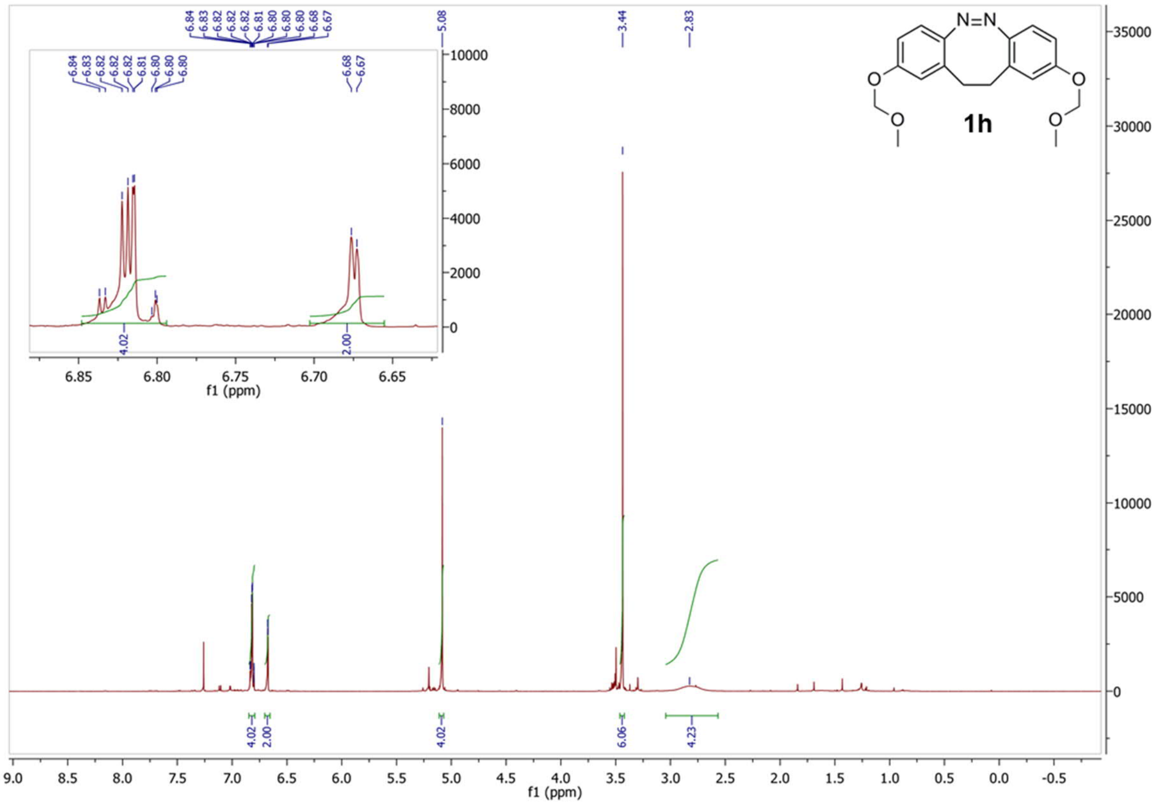

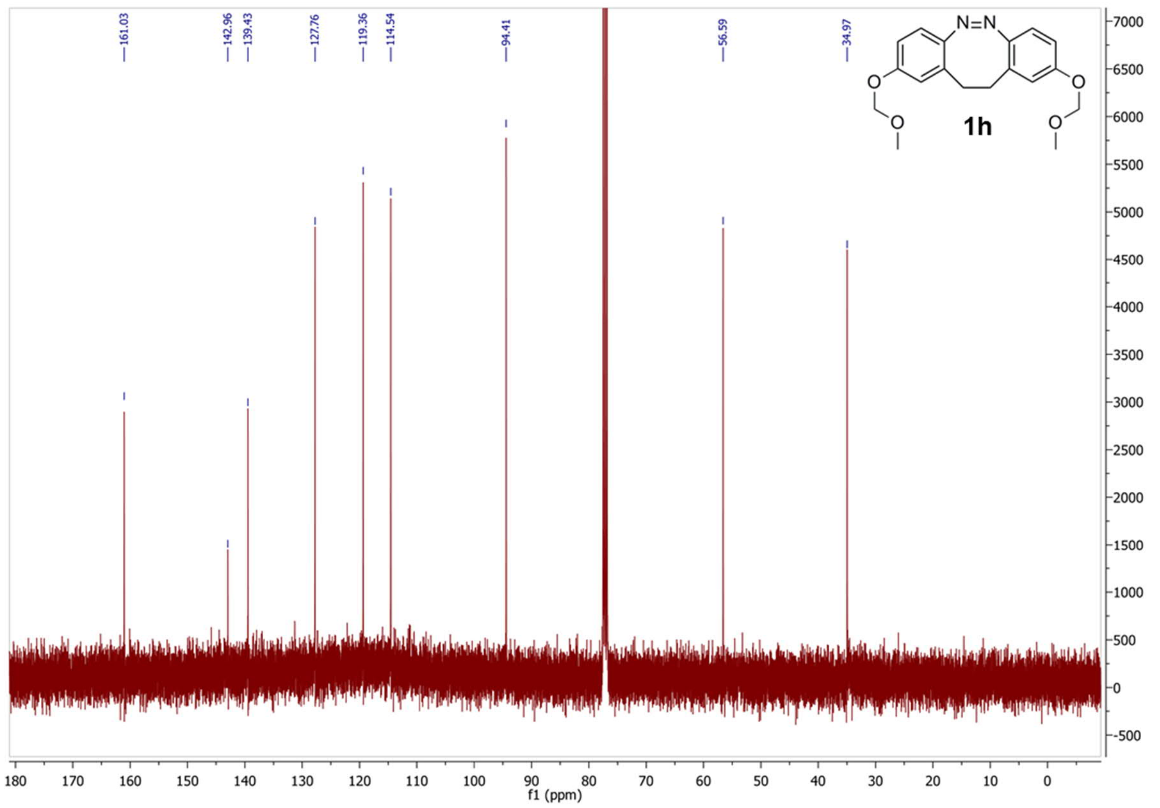

Figure S31. ^{1}H NMR (600 MHz) and ^{13}C NMR (151 MHz) spectra of 2,9-bis(methoxymethoxy)-11,12-dihydrodibenzo[*c*,*g*]-1,2-diazocine **1h** in $CDCl_3$.

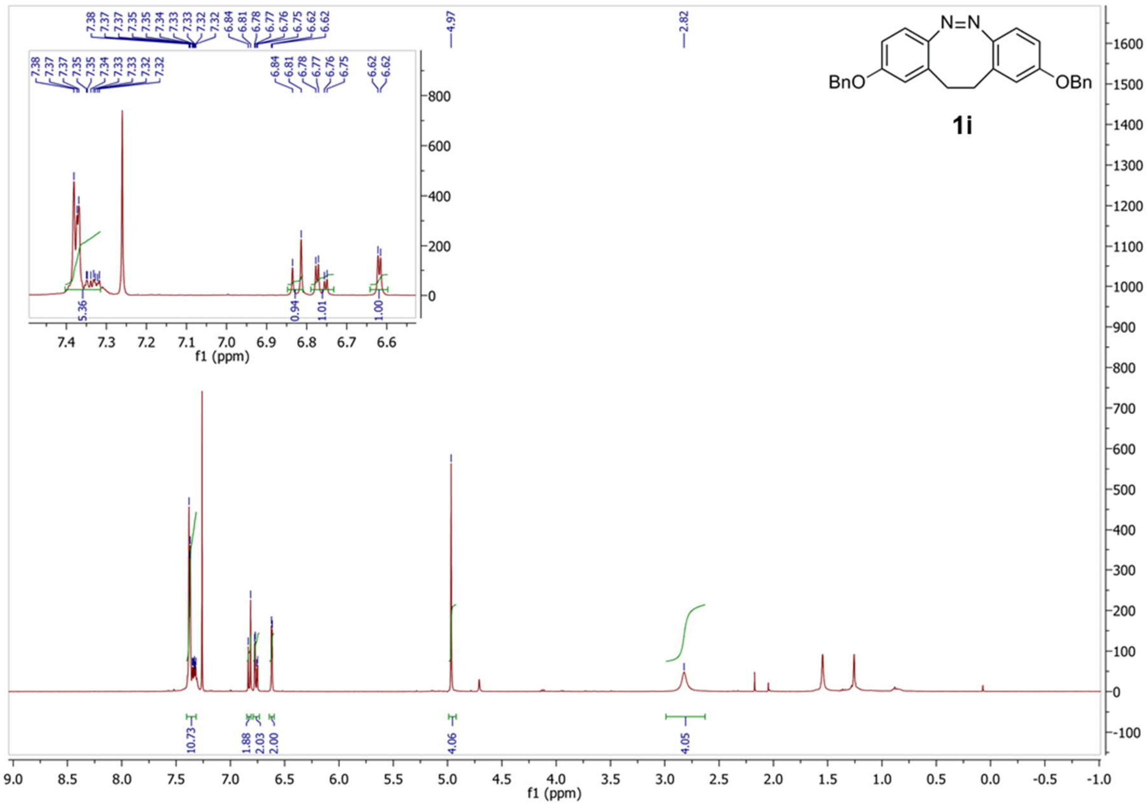

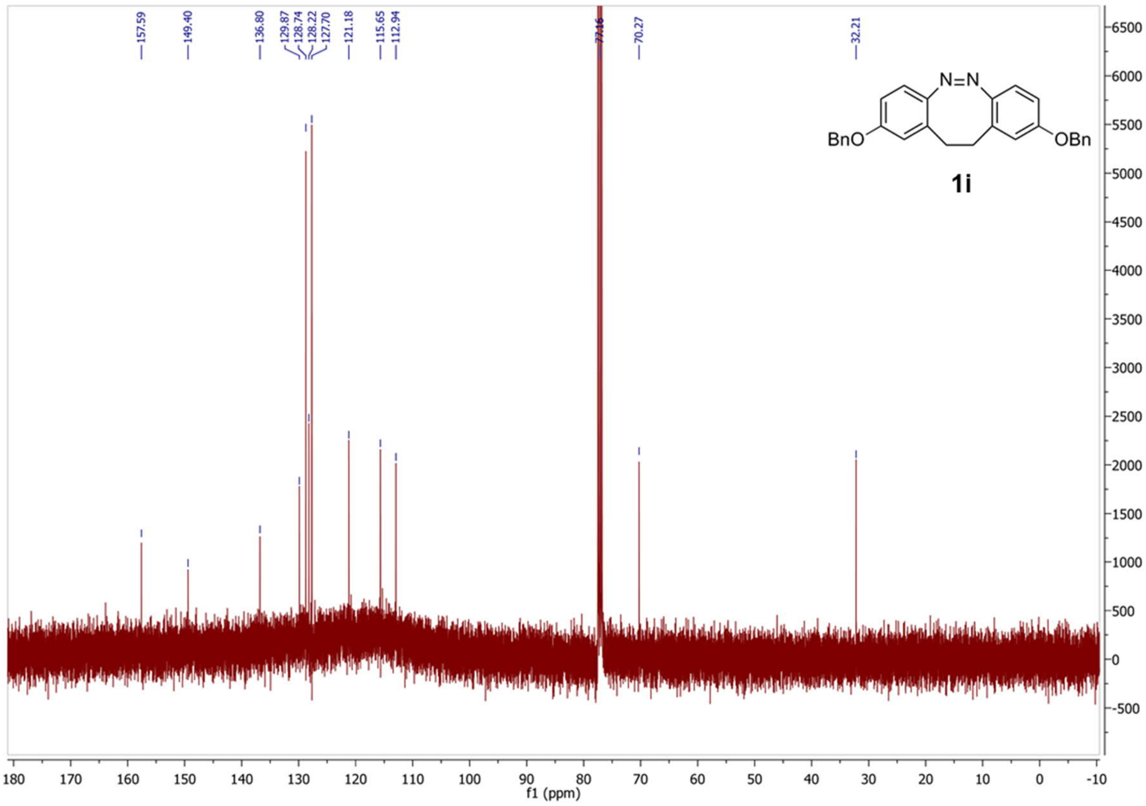

Figure S32. ^{1}H NMR (400 MHz) and ^{13}C NMR (101 MHz) spectra of 2,9-dibenzyloxy-11,12-dihydrodibenzo[*c*,*g*]-1,2-diazocine **1i** in $CDCl_3$.

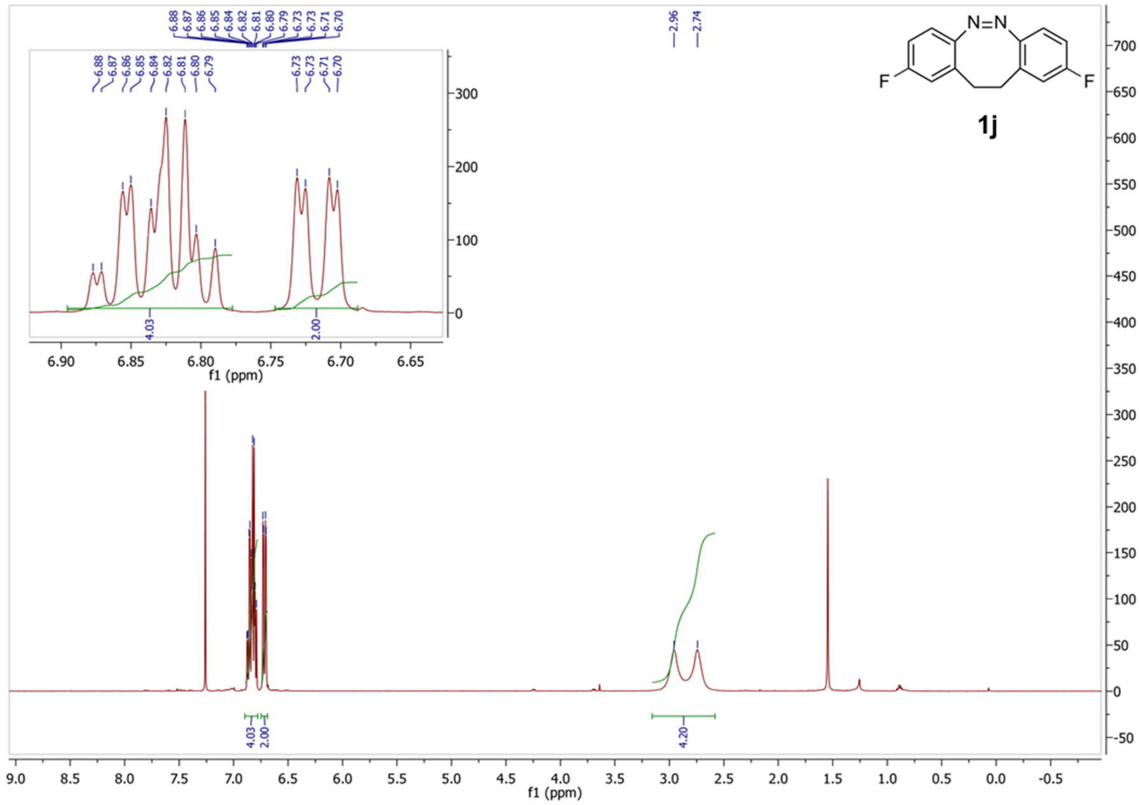

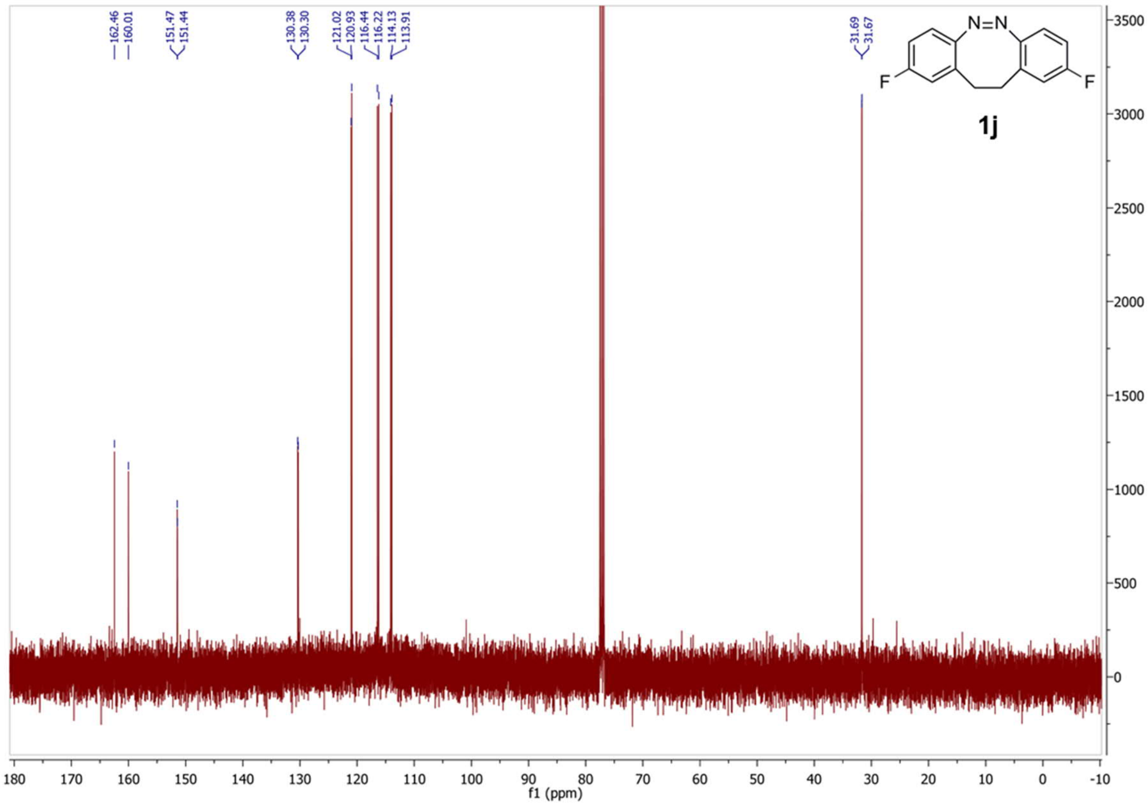

Figure S33. ^{1}H NMR (400 MHz) and ^{13}C NMR (101 MHz) spectra of 2,9-difluoro-11,12-dihydrodibenzo[*c*,*g*]-1,2-diazocine **1j** in $CDCl_3$.

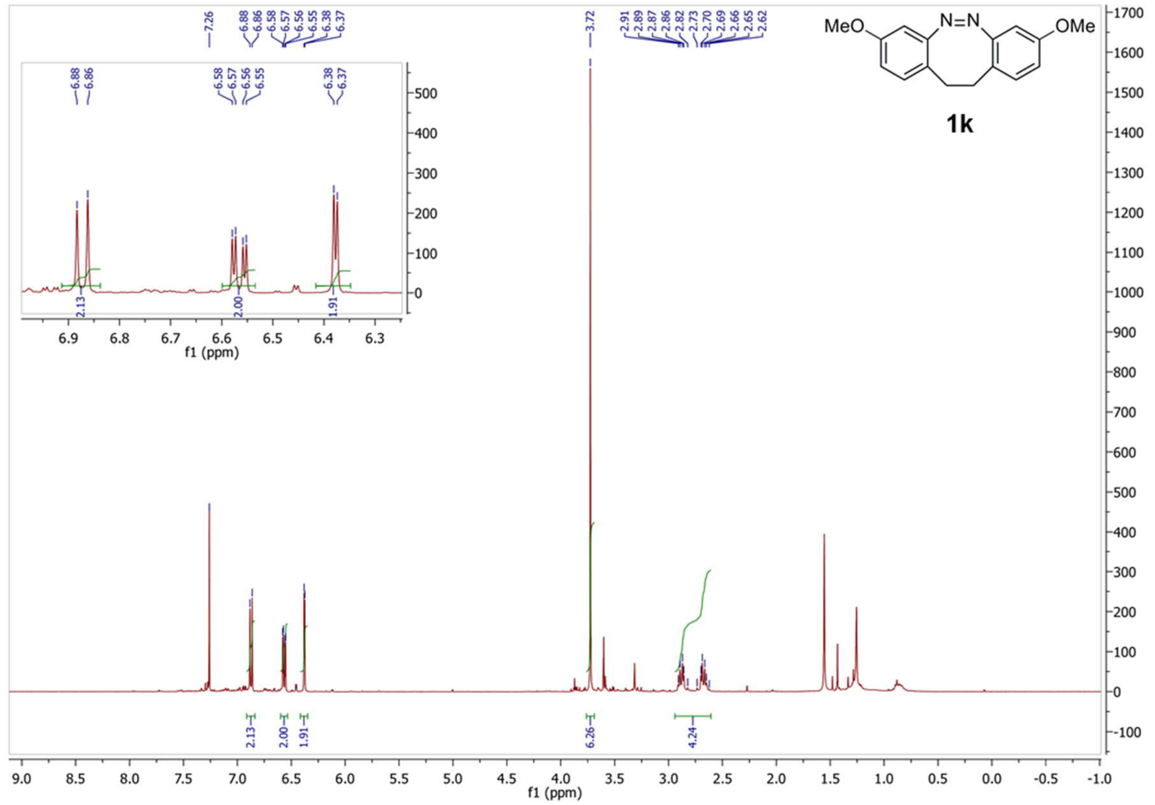

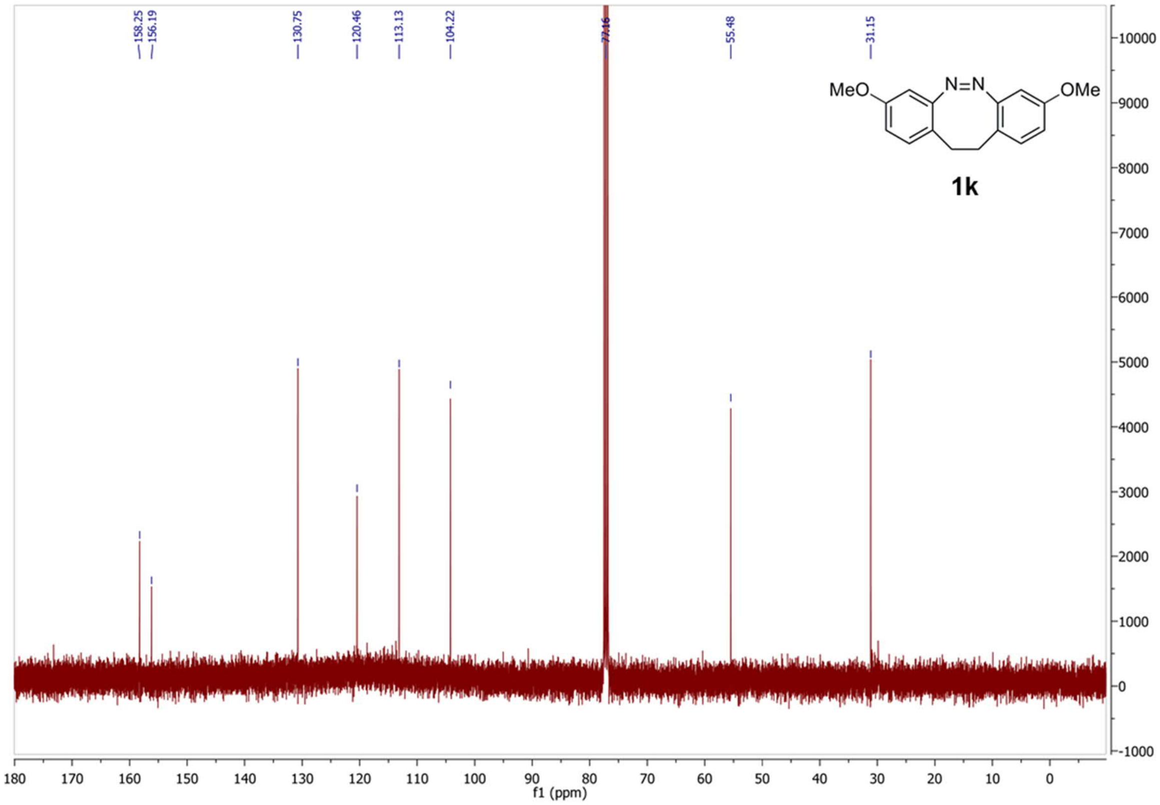

Figure S34. ^{1}H NMR (400 MHz) and ^{13}C NMR (101 MHz) spectra of 3,8-dimethoxy-11,12-dihydrodibenzo[*c*,*g*]-1,2-diazocine **1k** in $CDCl_3$.

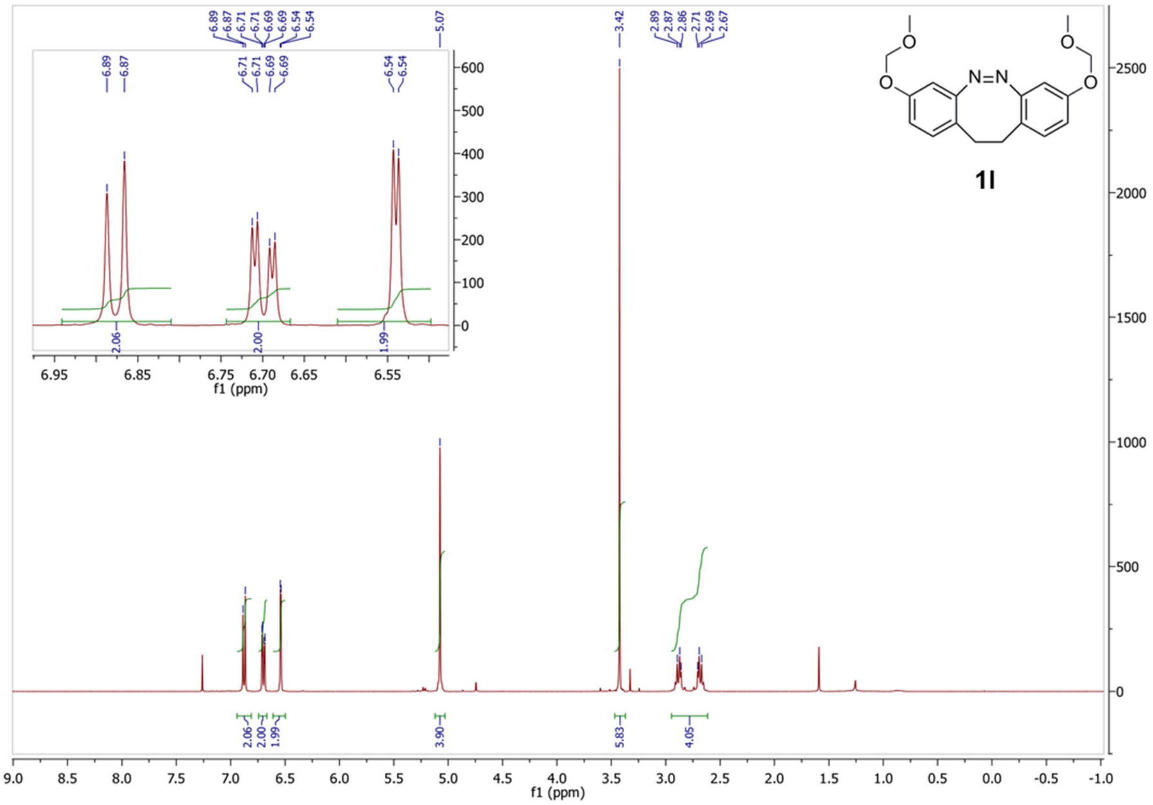

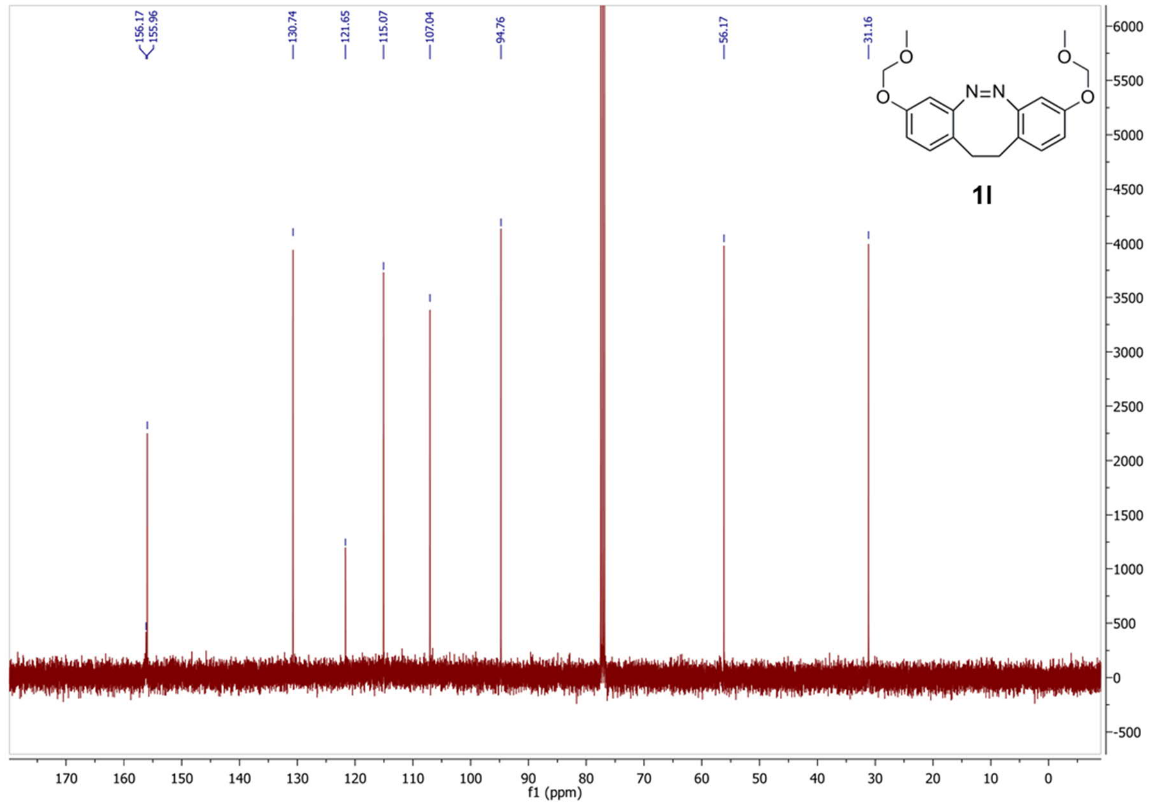

Figure S35. ^{1}H NMR (400 MHz) and ^{13}C NMR (101 MHz) spectra of 3,8-bis(methoxymethoxy)-11,12-dihydrodibenzo[*c*,*g*]-1,2-diazocine **1l** in $CDCl_3$.

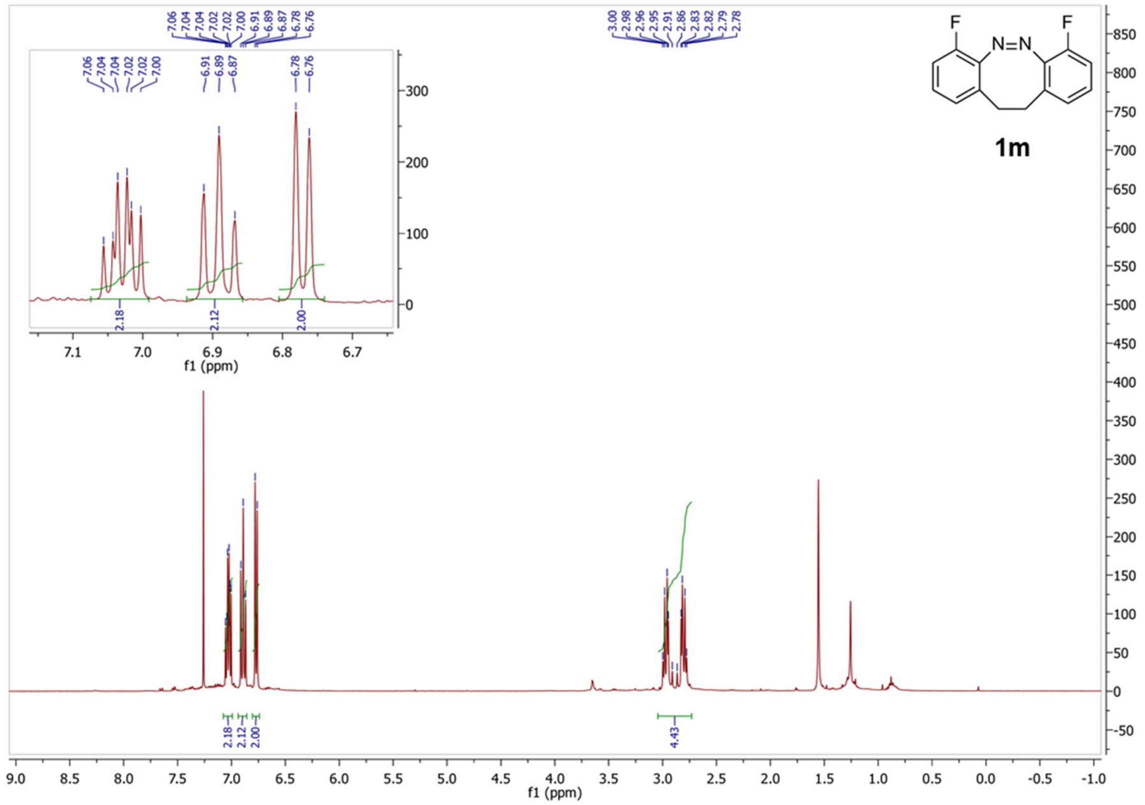

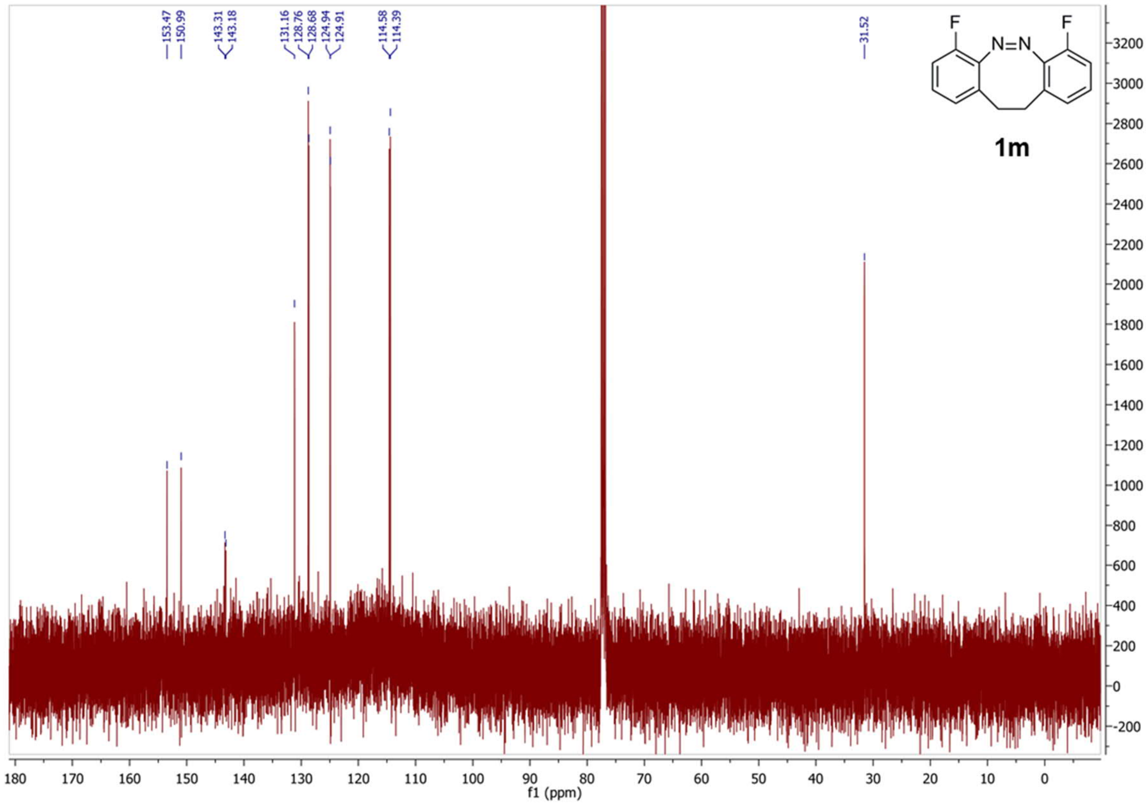

Figure S36. ^{1}H NMR (400 MHz) and ^{13}C NMR (101 MHz) spectra of 4,7-difluoro-11,12-dihydrodibenzo[*c*,*g*]-1,2-diazocine **1m** in $CDCl_3$.

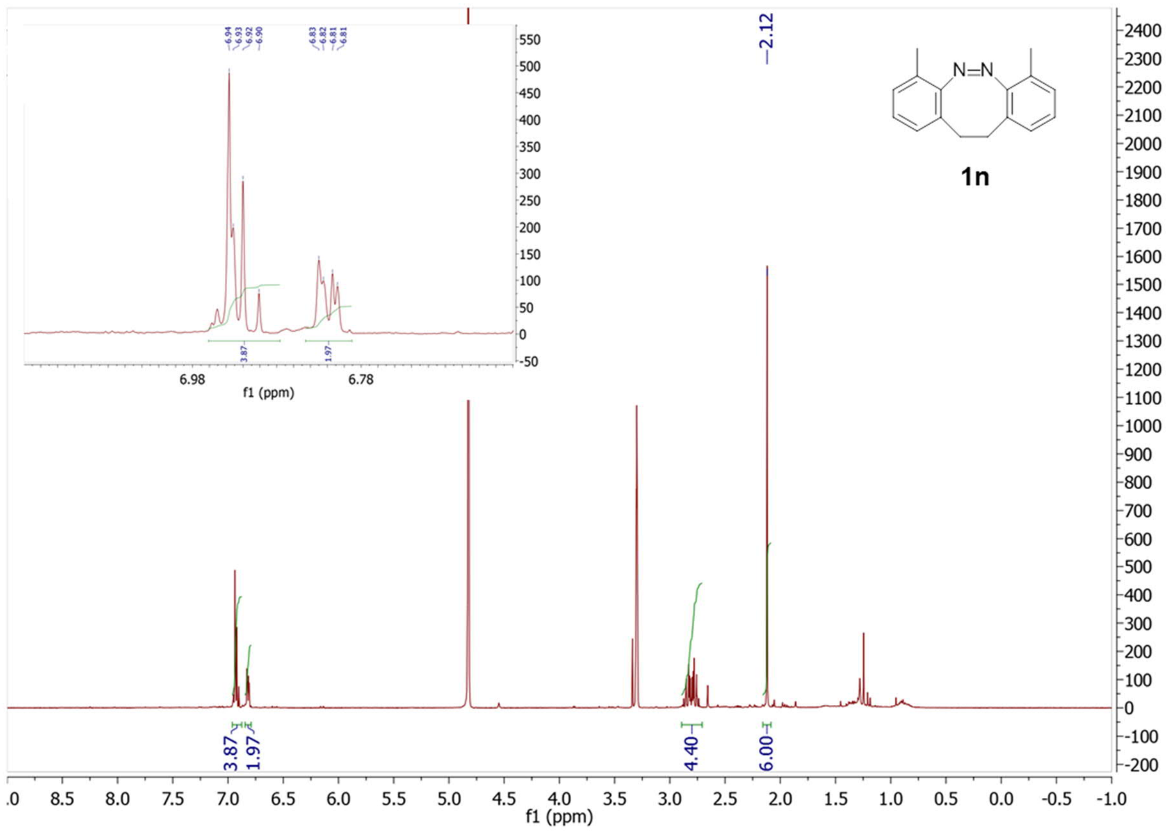

Figure S37. ^{1}H NMR (400 MHz) spectrum of 4,7-dimethyl-11,12-dihydrodibenzo[*c*,*g*]-1,2-diazocine **1n** in $CDCl_3$.

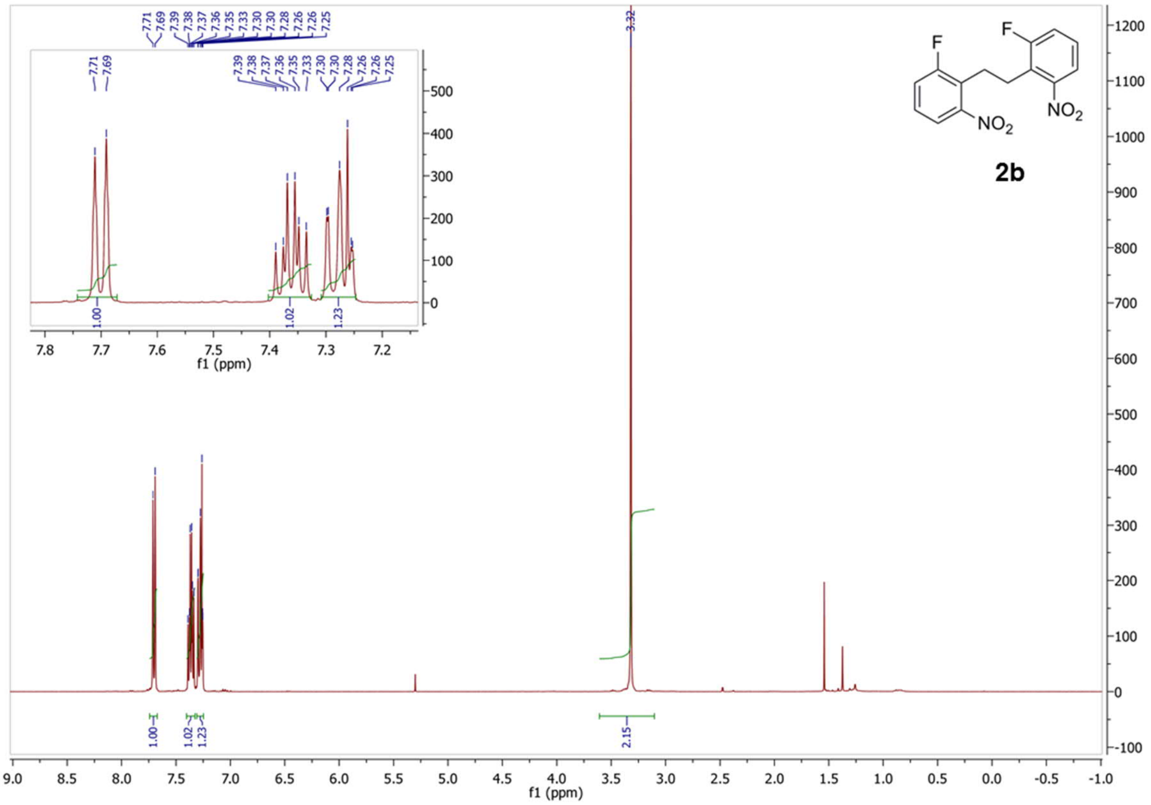

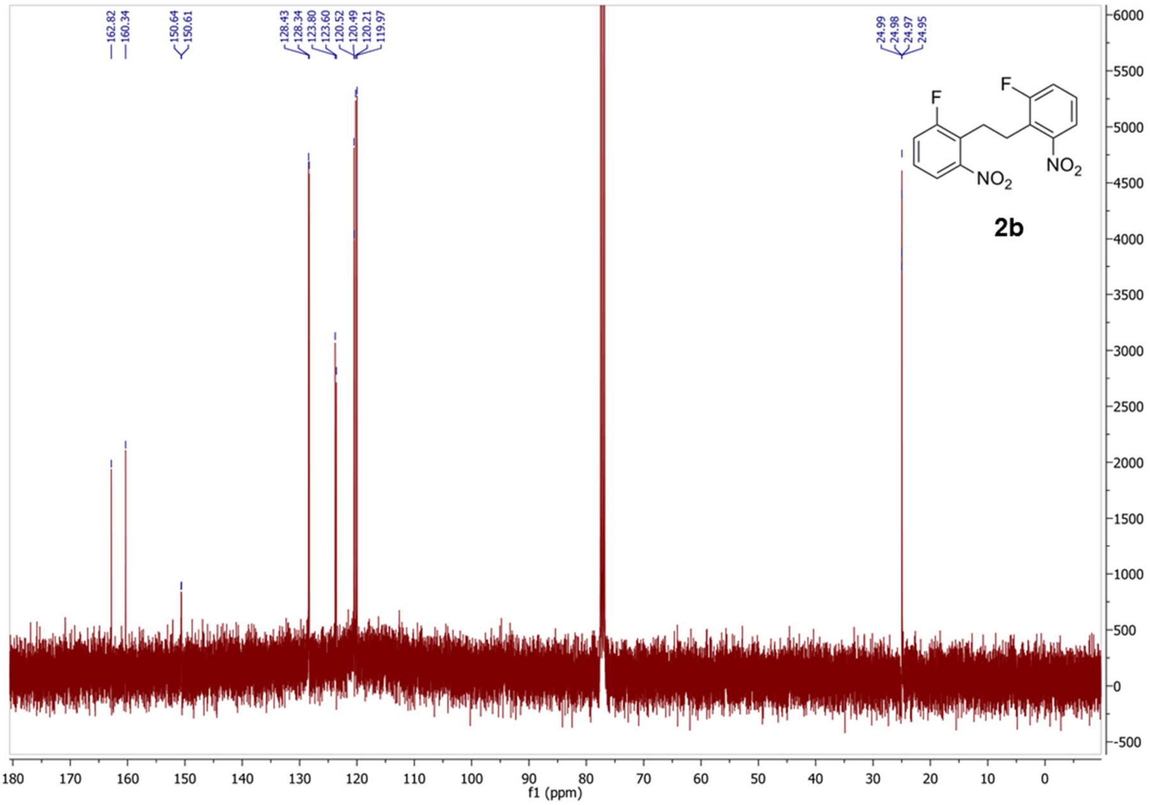

Figure S38. ^{1}H NMR (400 MHz) and ^{13}C NMR (101 MHz) spectra of 1,2-bis(2-fluoro-6-nitrophenyl)ethane **2b** in $CDCl_3$.

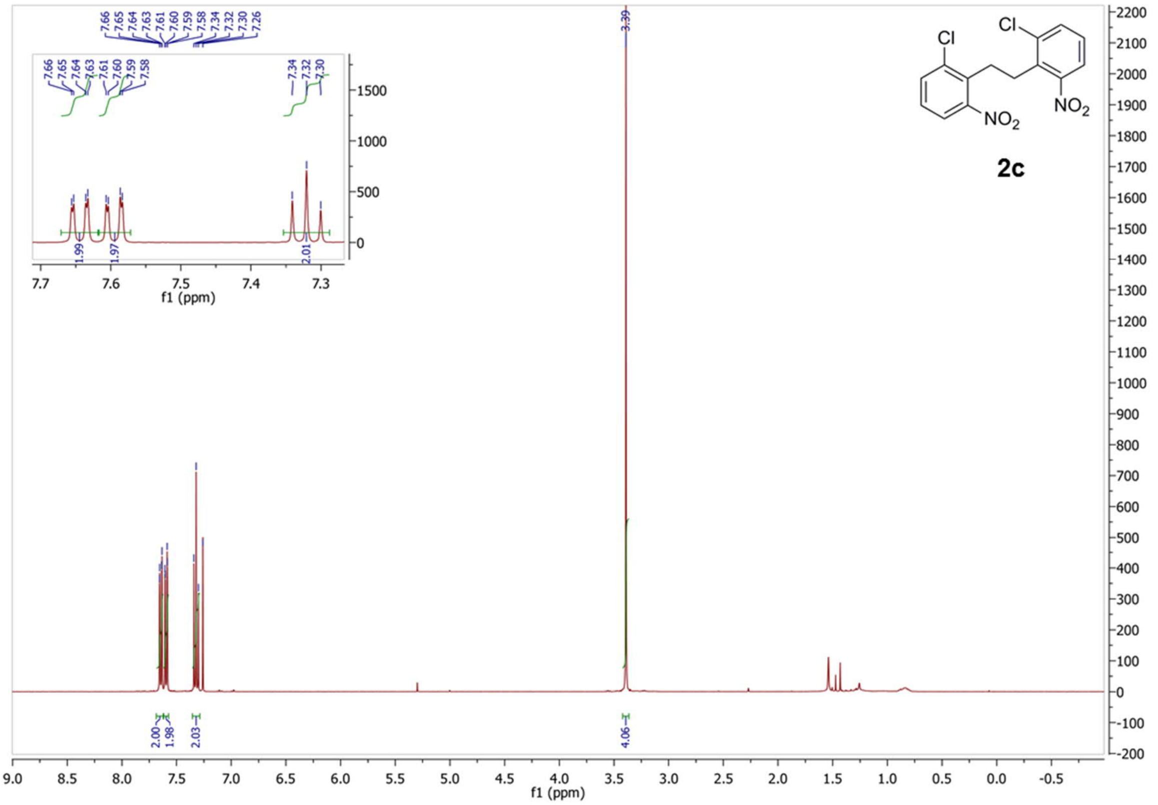

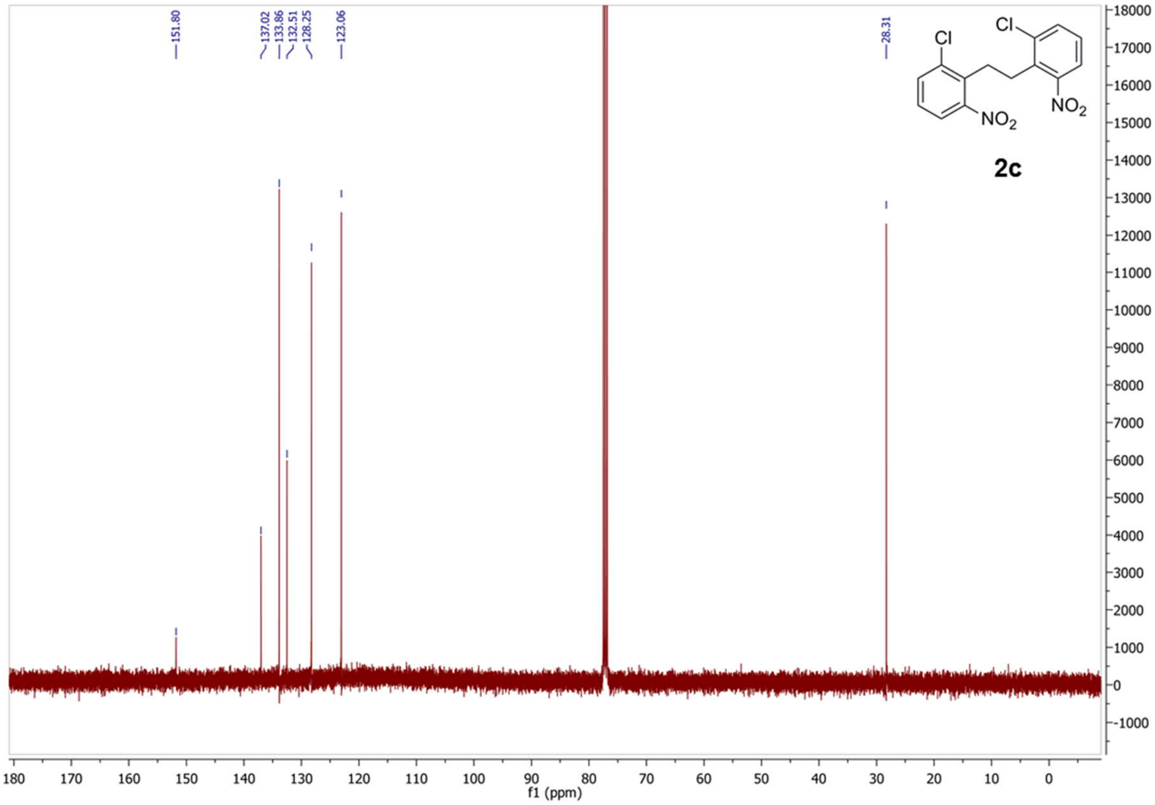

Figure S39. ^{1}H NMR (400 MHz) and ^{13}C NMR (101 MHz) spectra of 1,2-bis(2-chloro-6-nitrophenyl)ethane **2c** in $CDCl_3$.

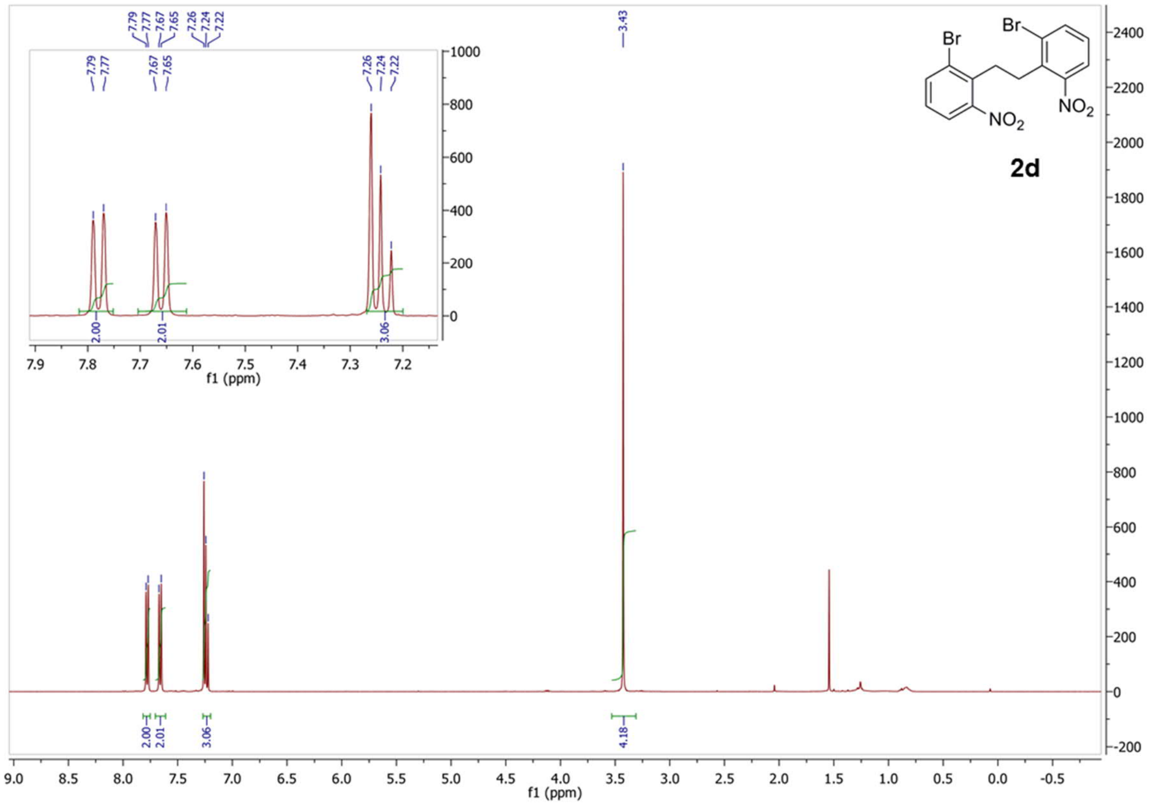

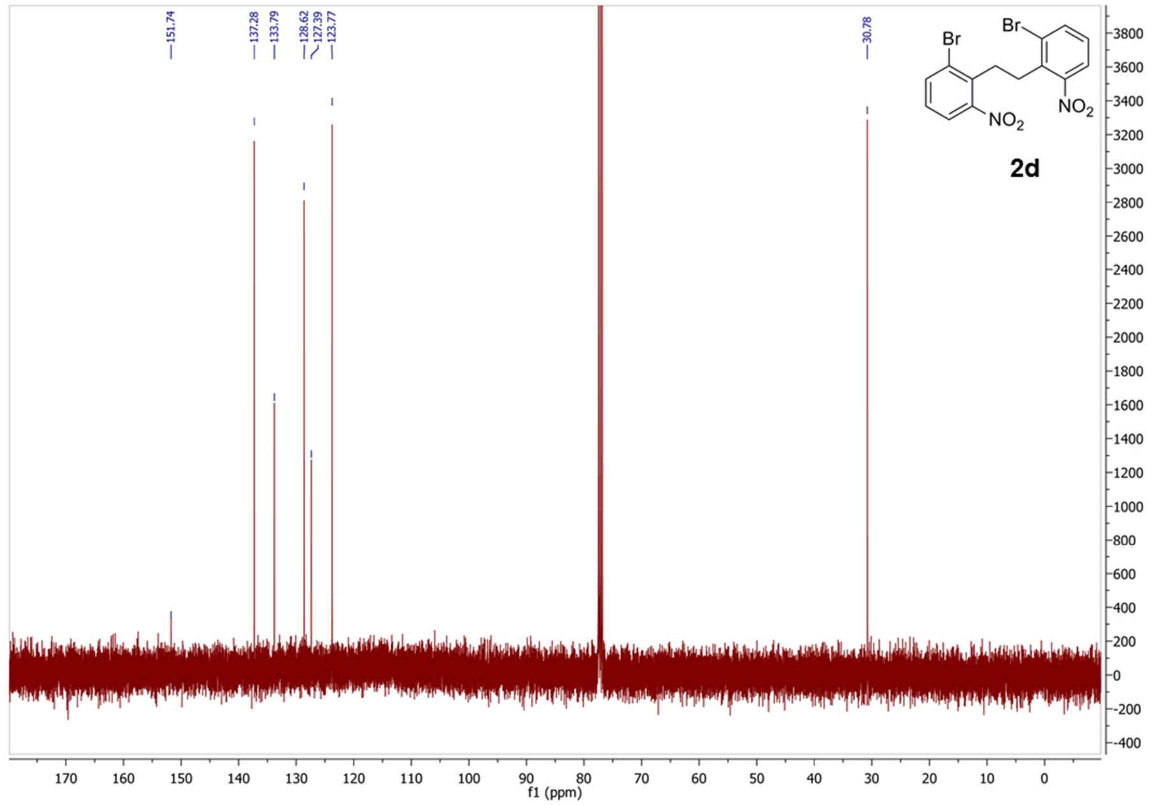

Figure S40. ^{1}H NMR (400 MHz) and ^{13}C NMR (101 MHz) spectra of 1,2-bis(2-bromo-6-nitrophenyl)ethane **2d** in $CDCl_3$.

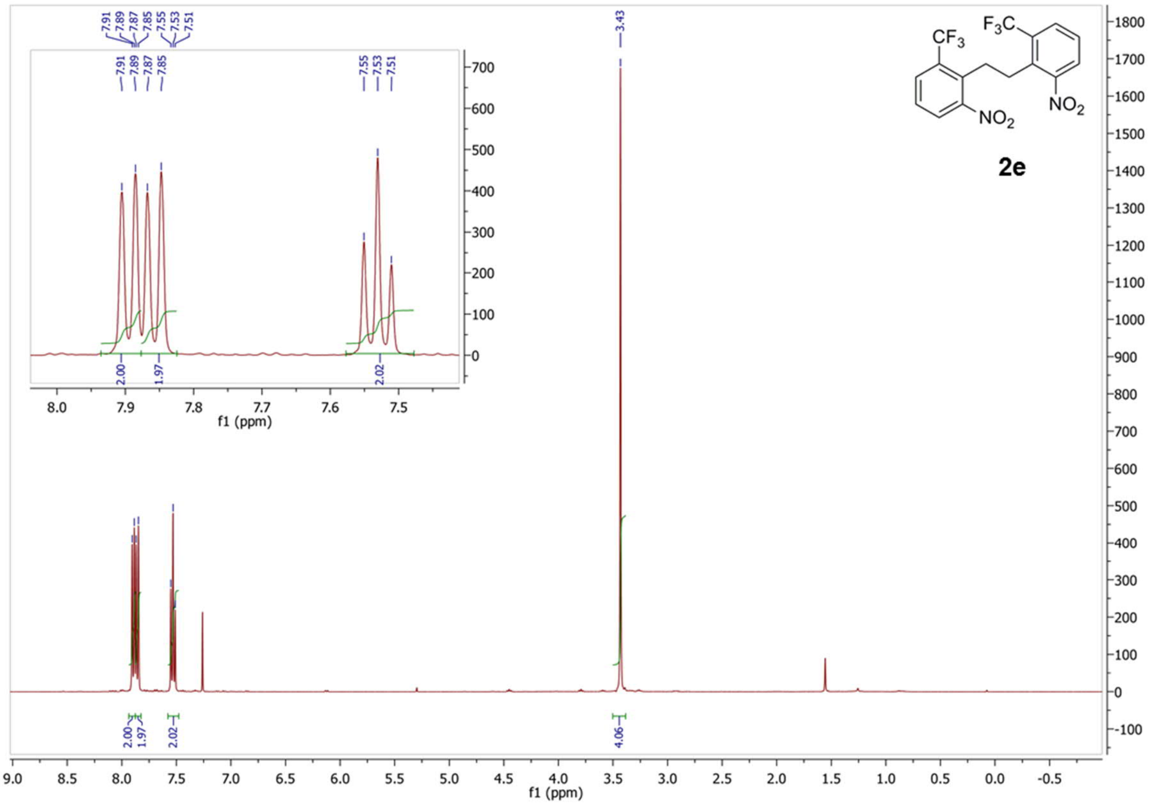

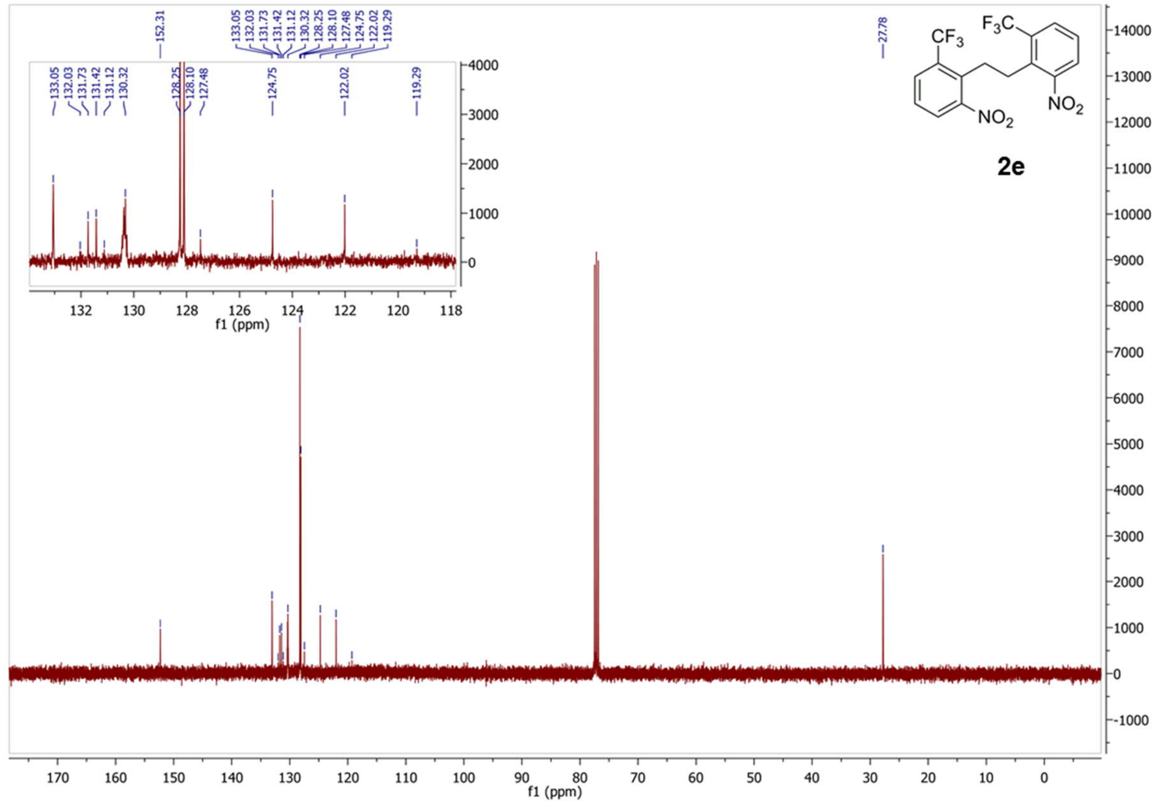

Figure S41. ^{1}H NMR (400 MHz) and ^{13}C NMR (101 MHz) spectra of 1,2-bis(6-trifluoromethyl-2-nitrophenyl)ethane **2e** in $CDCl_3$.

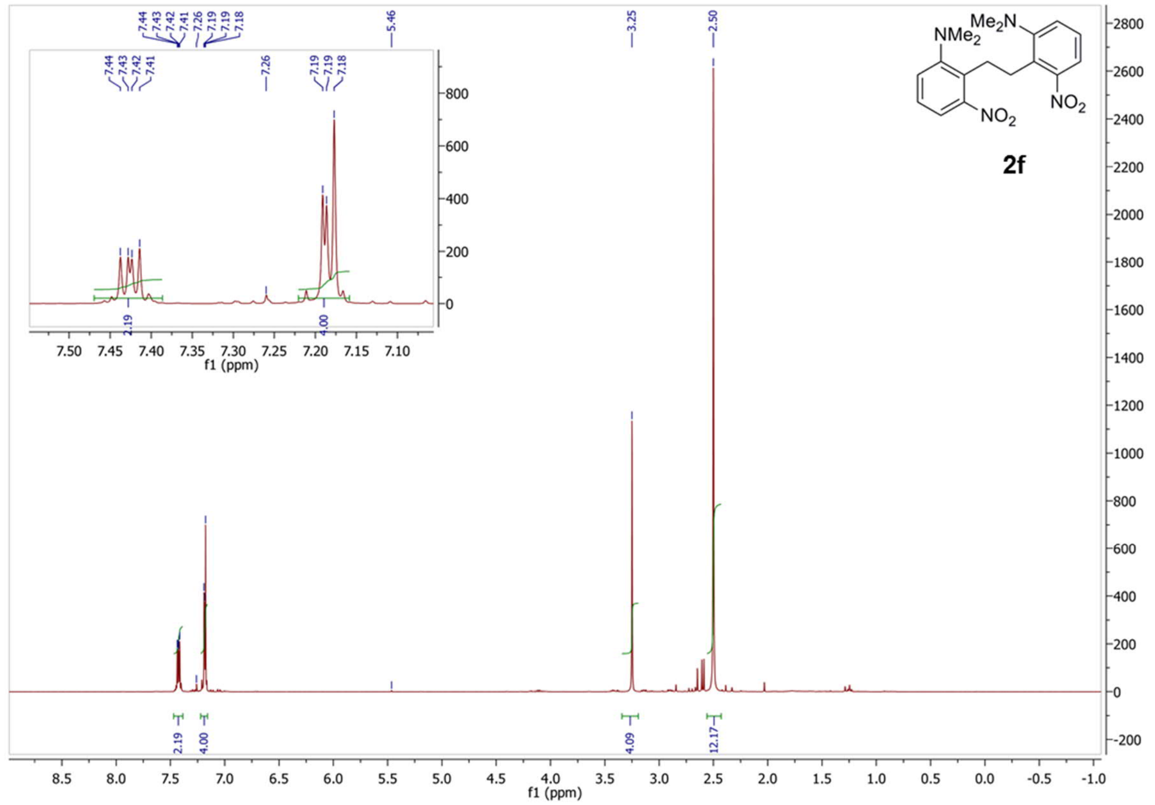

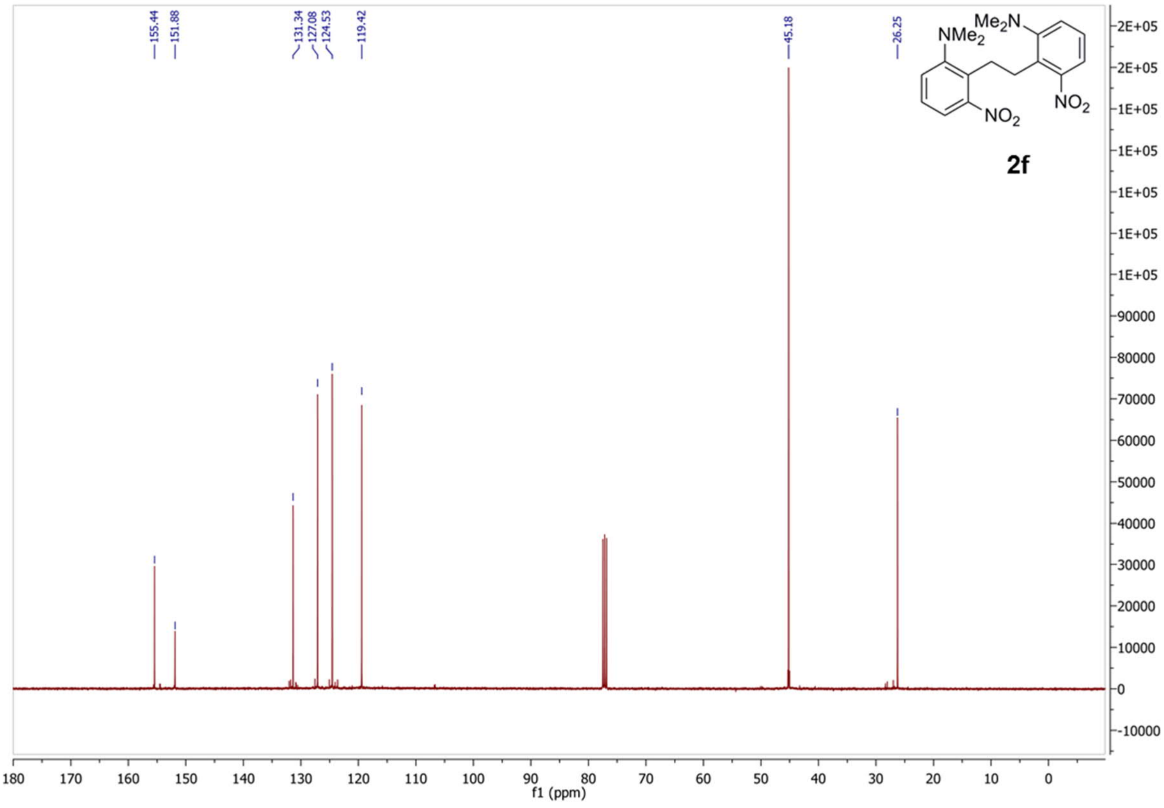

Figure S42. ^{1}H NMR (400 MHz) and ^{13}C NMR (101 MHz) spectra of 1,2-bis(2-(dimethylamino)-6-nitrophenyl)ethane **2f** in $CDCl_3$.

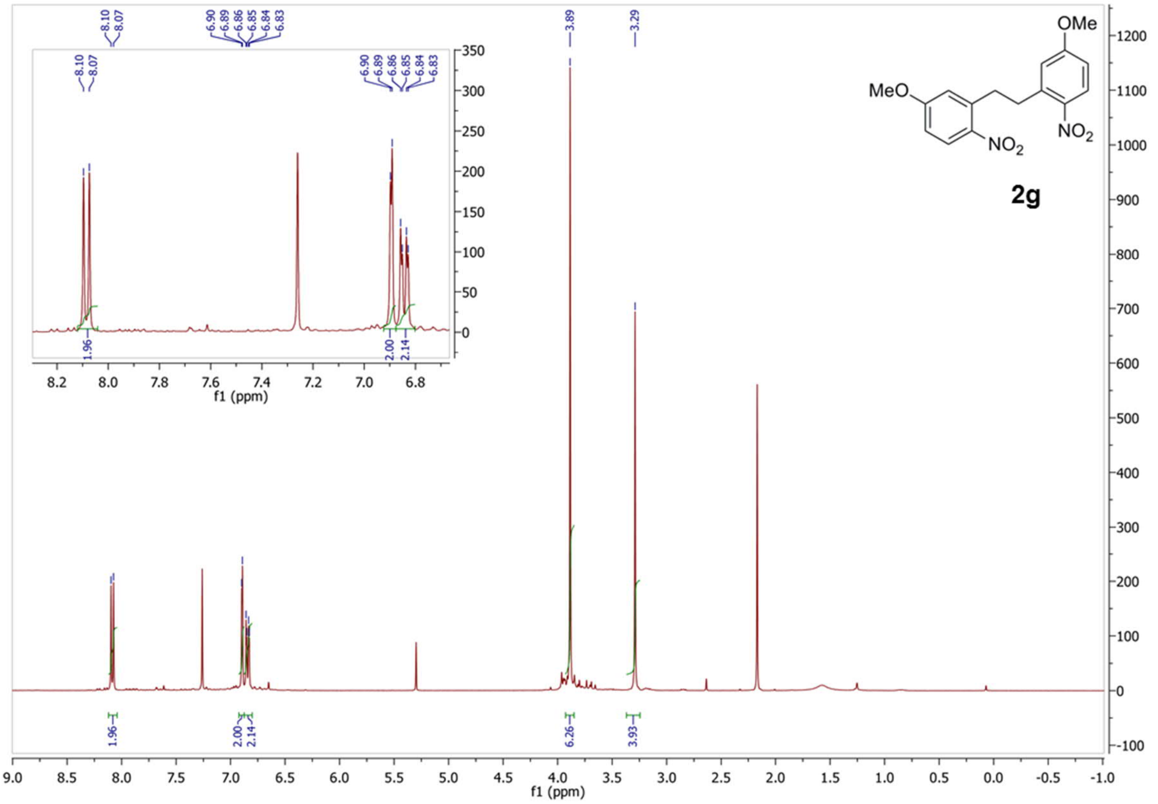

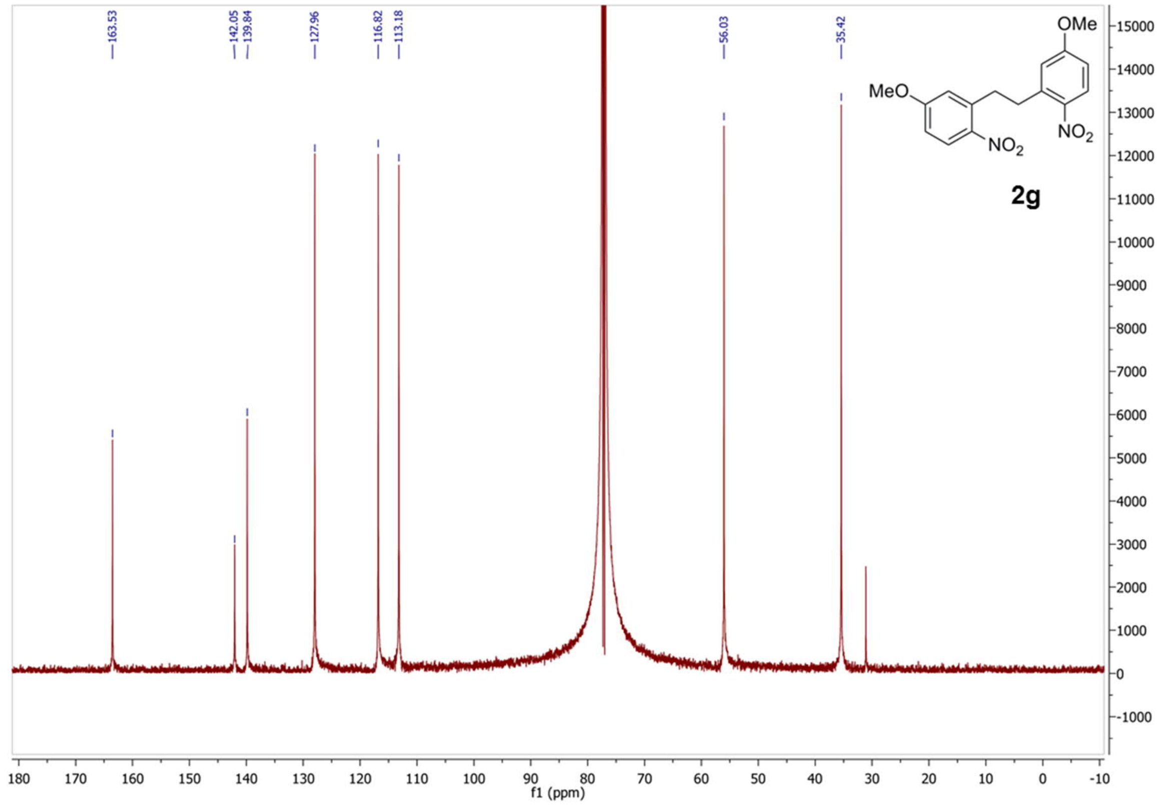

Figure S43. ^{1}H NMR (400 MHz) and ^{13}C NMR (126 MHz) spectra of 1,2-bis(5-methoxy-2-nitrophenyl)ethane **2g** in $CDCl_3$.

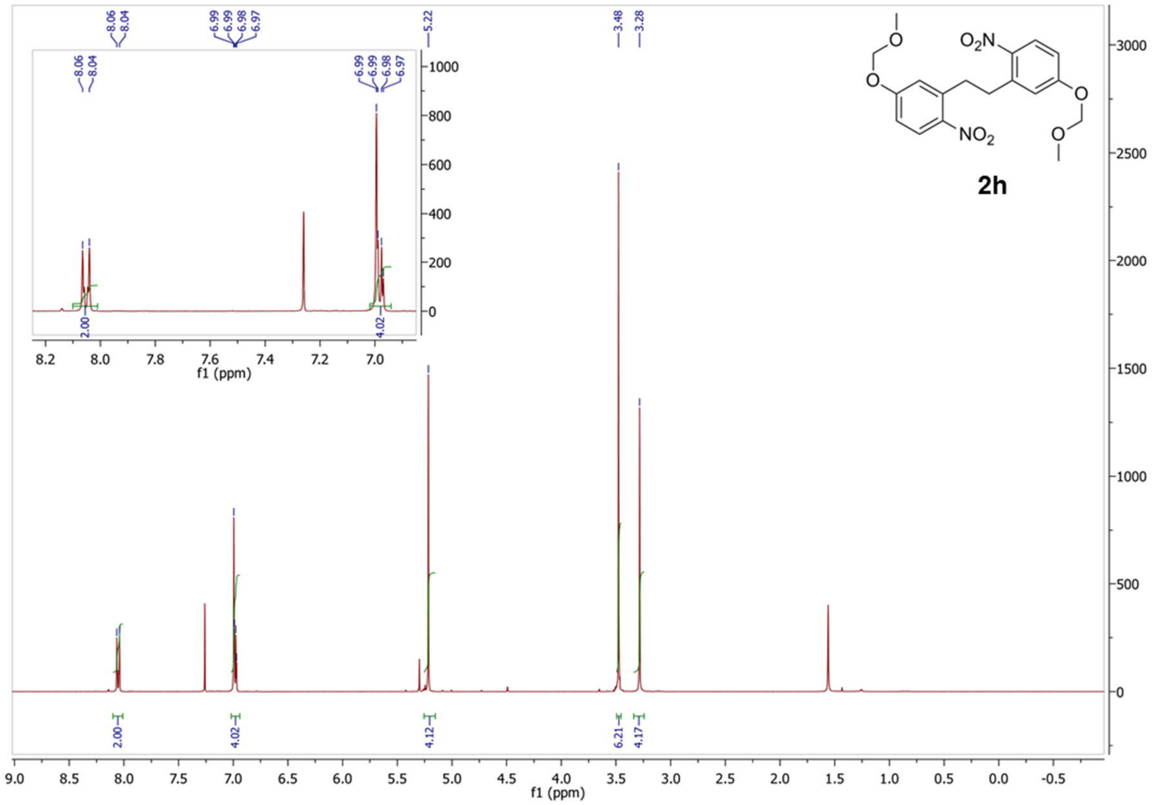

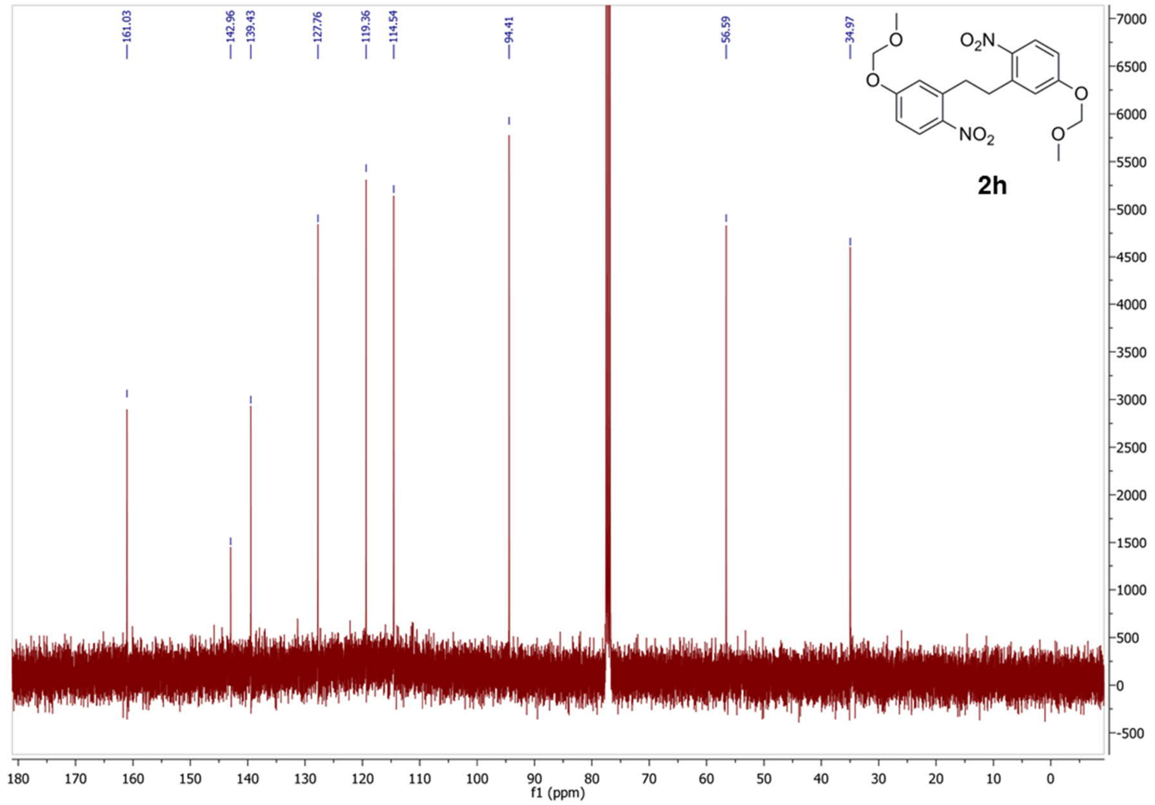

Figure S44. ^{1}H NMR (400 MHz) and ^{13}C NMR (101 MHz) spectra of 1,2-bis(5-methoxymethoxy-2-nitrophenyl)ethane **2h** in $CDCl_3$.

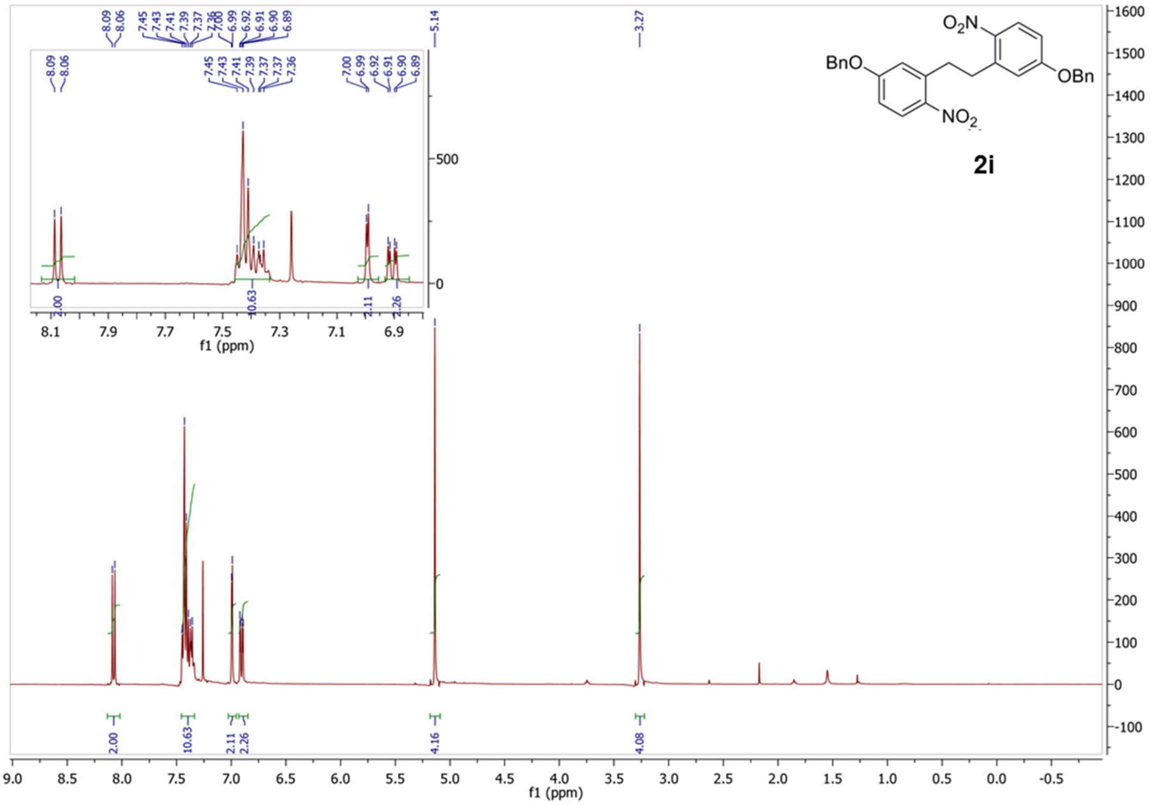

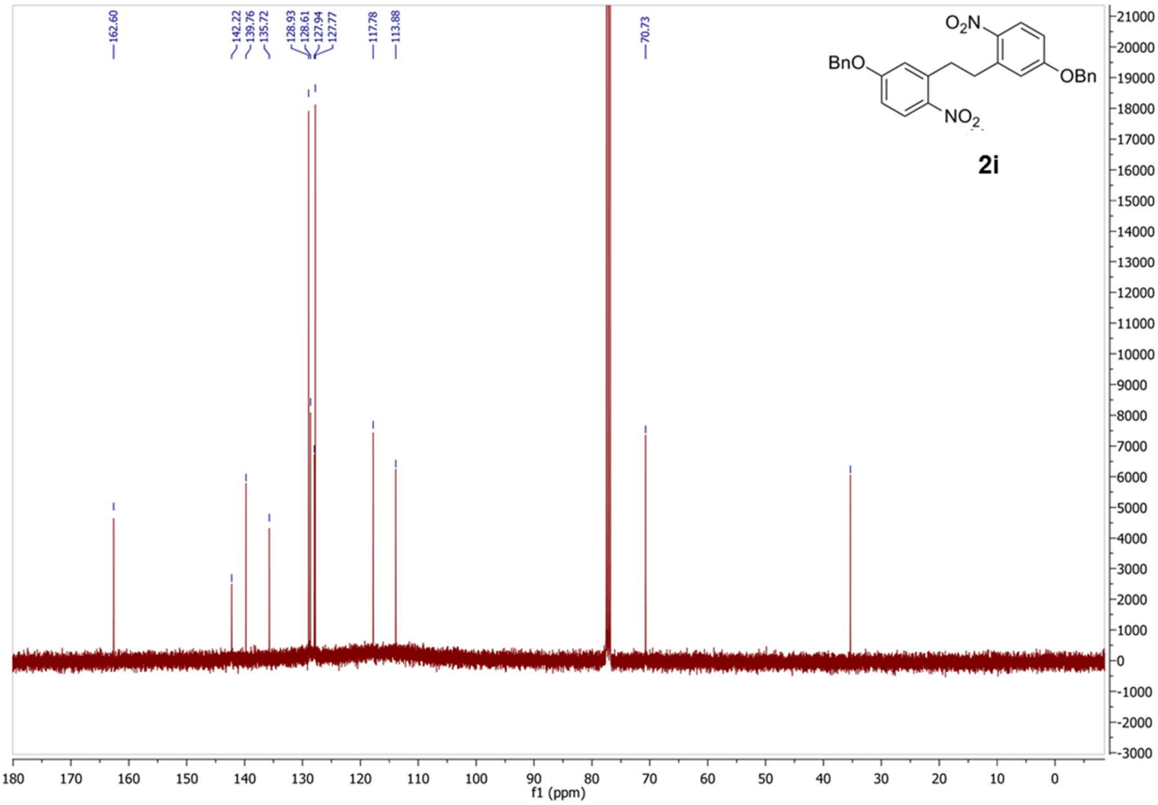

Figure S45. ^{1}H NMR (400 MHz) and ^{13}C NMR (101 MHz) spectra of 1,2-bis(5-benzyloxy-2-nitrophenyl)ethane **2i** in $CDCl_3$.

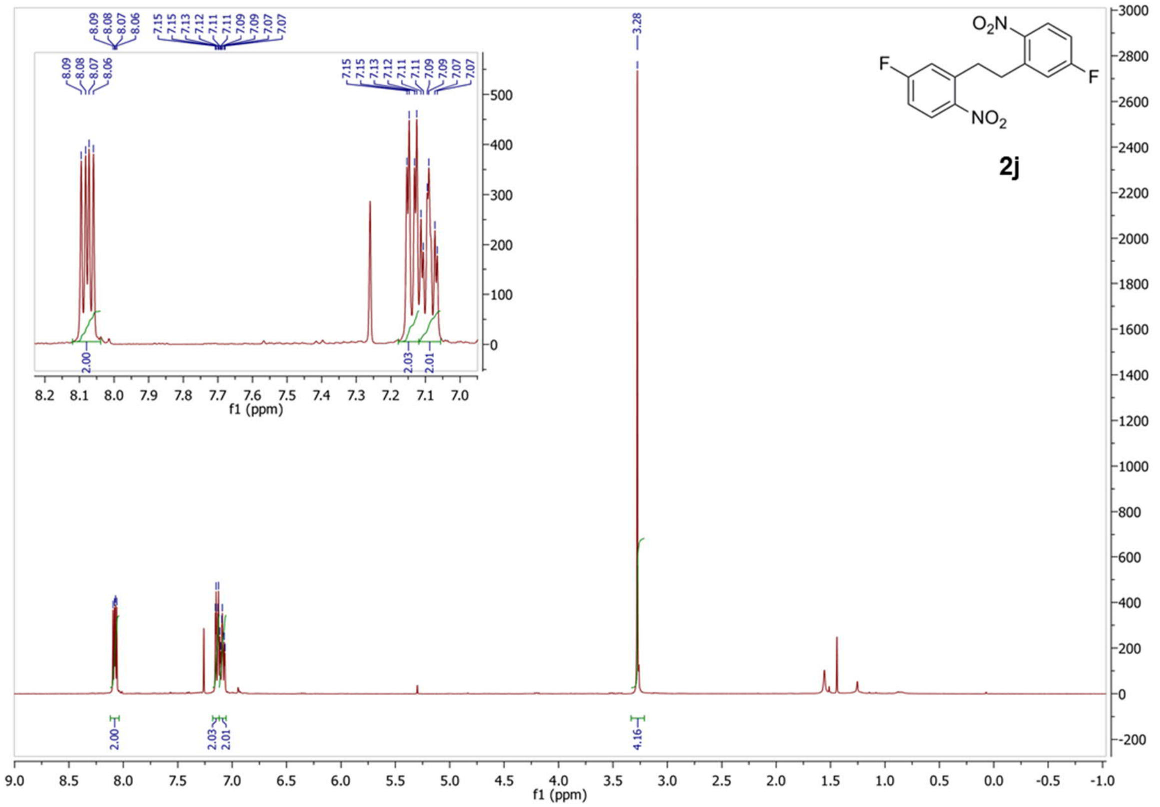

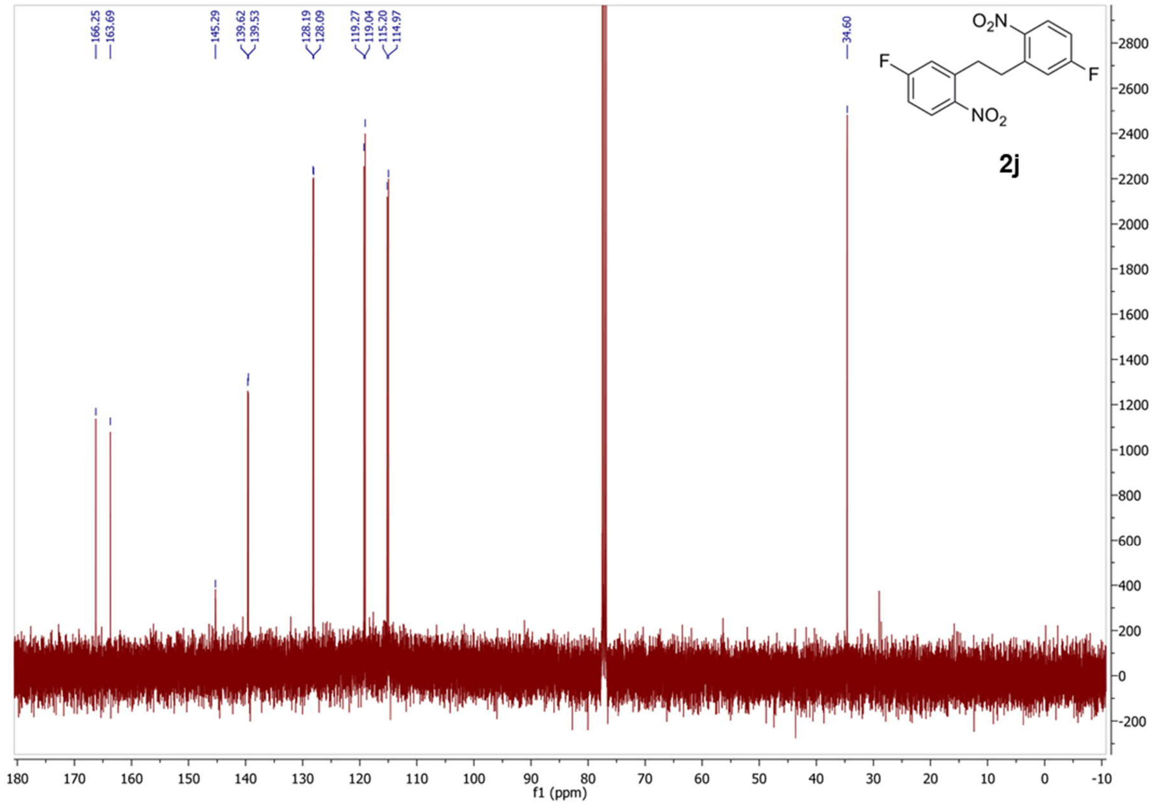

Figure S46. ^{1}H NMR (400 MHz) and ^{13}C NMR (101 MHz) spectra of 1,2-bis(3-fluoro-6-nitrophenyl)ethane **2j** in $CDCl_3$.

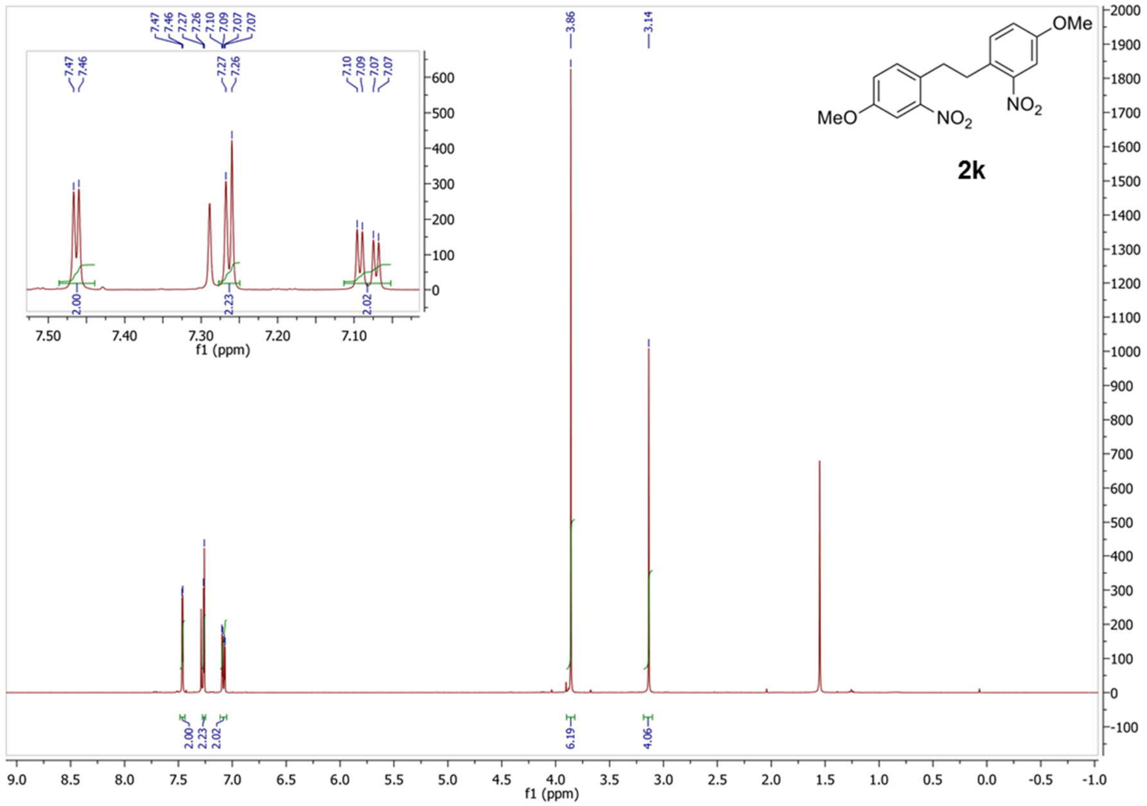

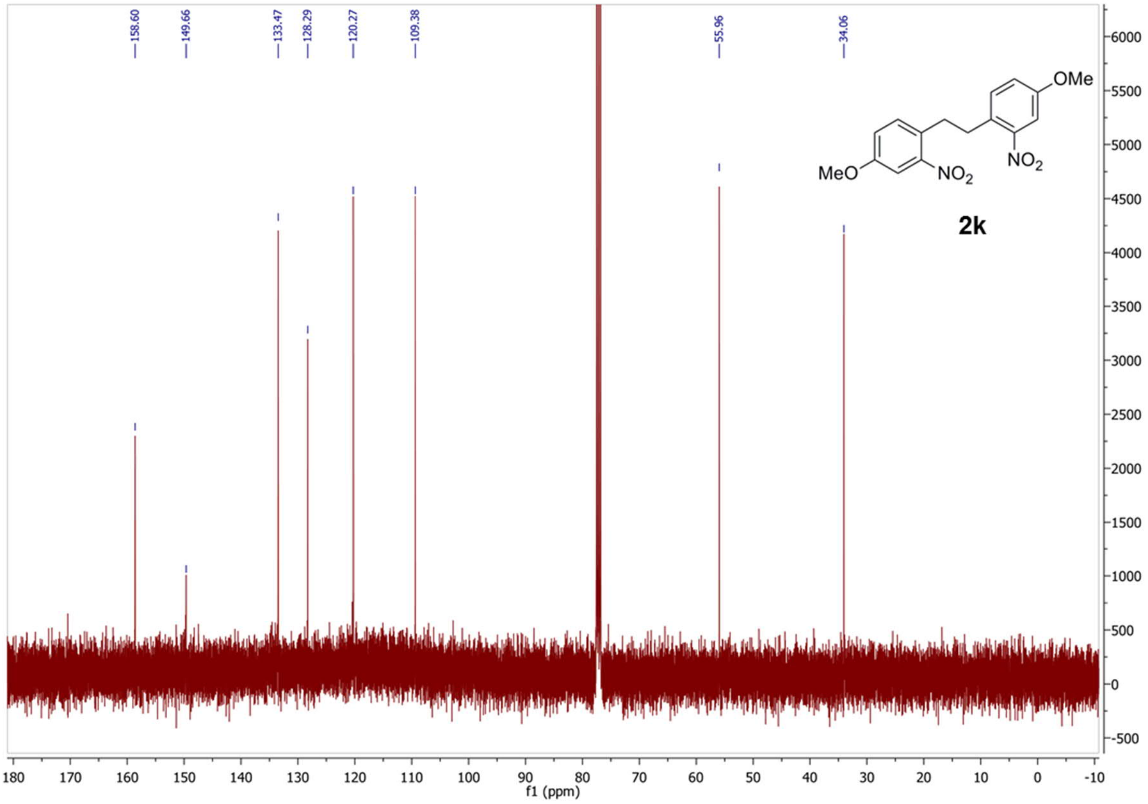

Figure S47. ^{1}H NMR (400 MHz) and ^{13}C NMR (101 MHz) spectra of 1,2-bis(4-methoxy-2-nitrophenyl)ethane **2k** in $CDCl_3$.

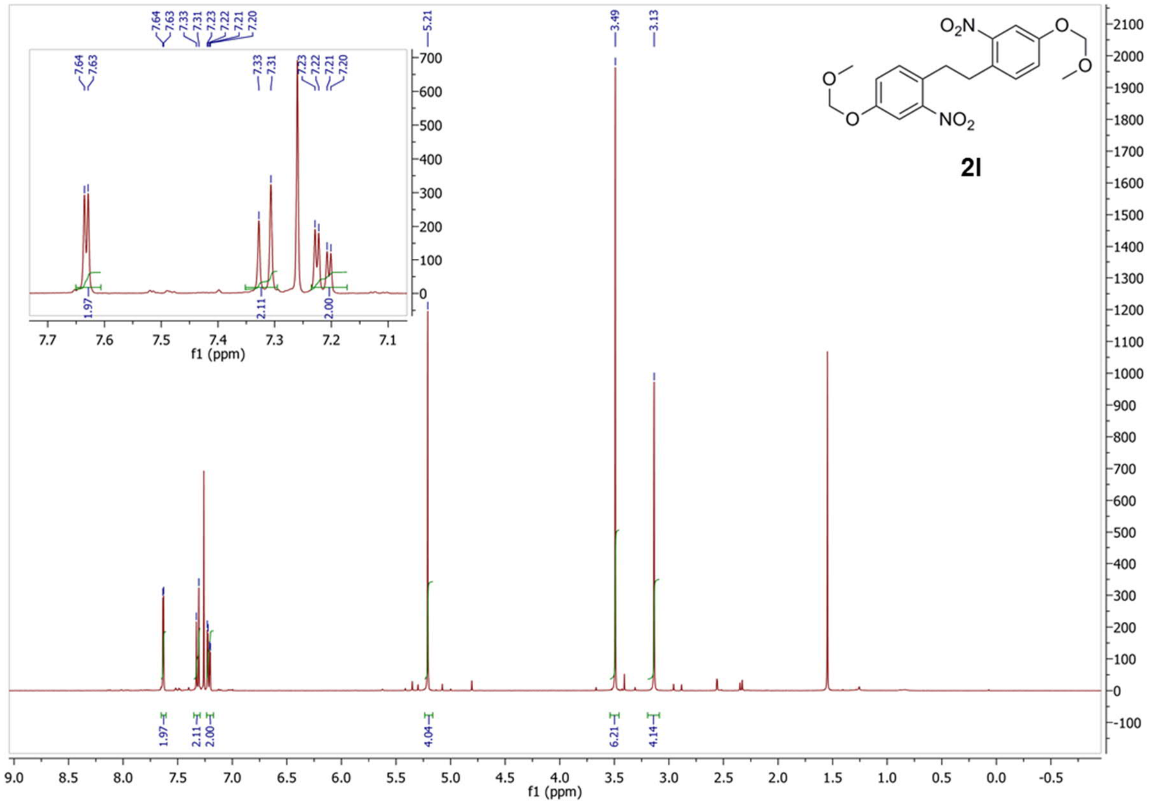

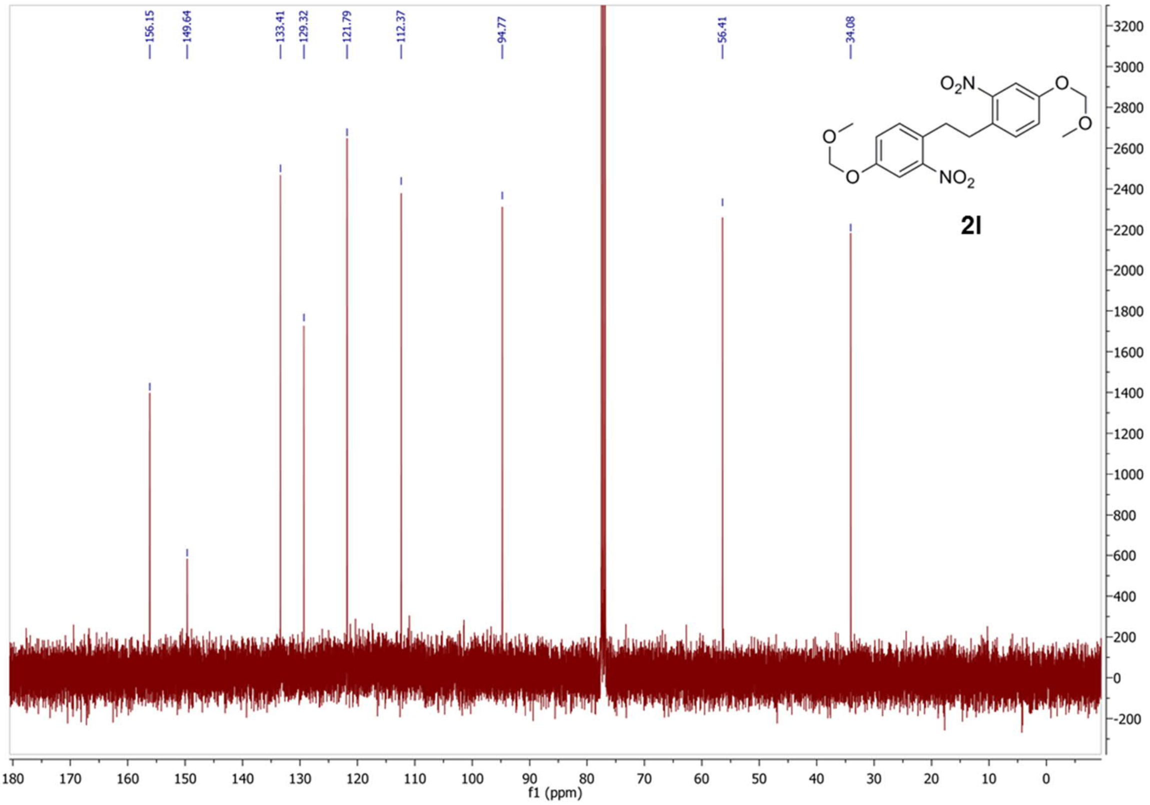

Figure S48. ^{1}H NMR (400 MHz) and ^{13}C NMR (101 MHz) spectra of 1,2-bis(4-(methoxymethoxy)-2-nitrophenyl)ethane **2l** in $CDCl_3$.

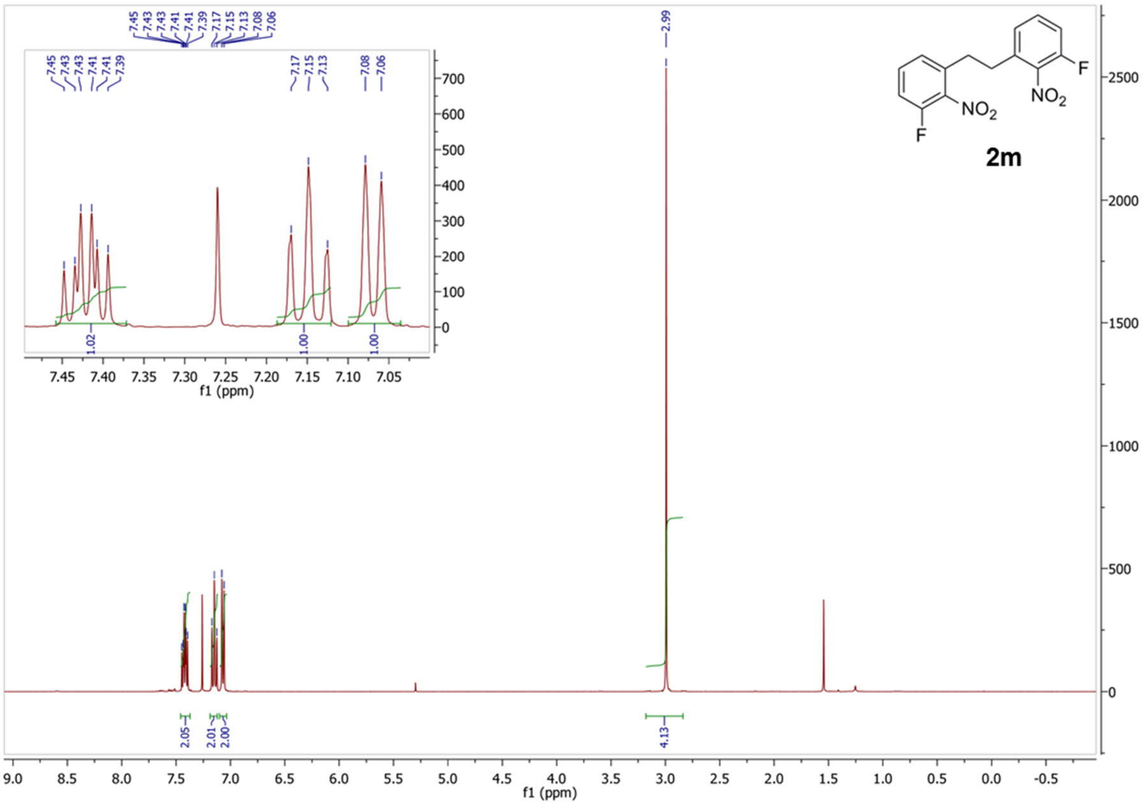

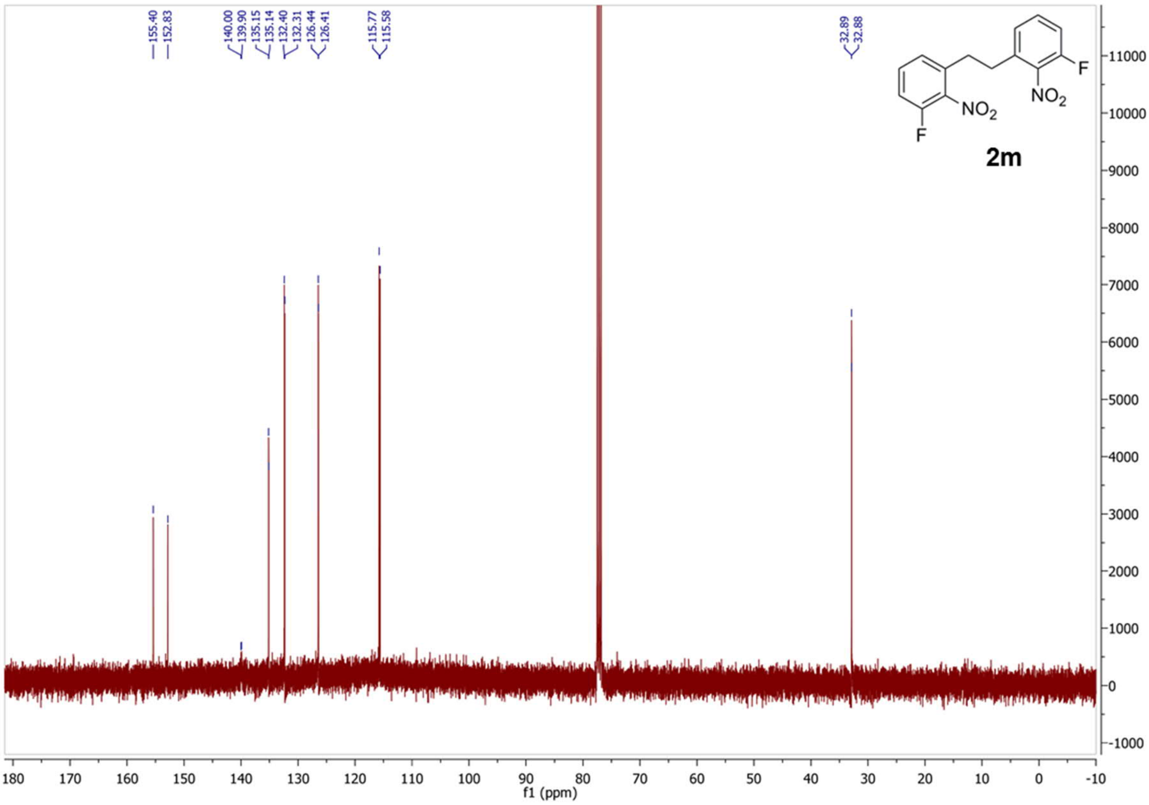

Figure S49. ^{1}H NMR (400 MHz) and ^{13}C NMR (101 MHz) spectra of 1,2-bis(3-fluoro-2-nitrophenyl)ethane **2m** in $CDCl_3$.

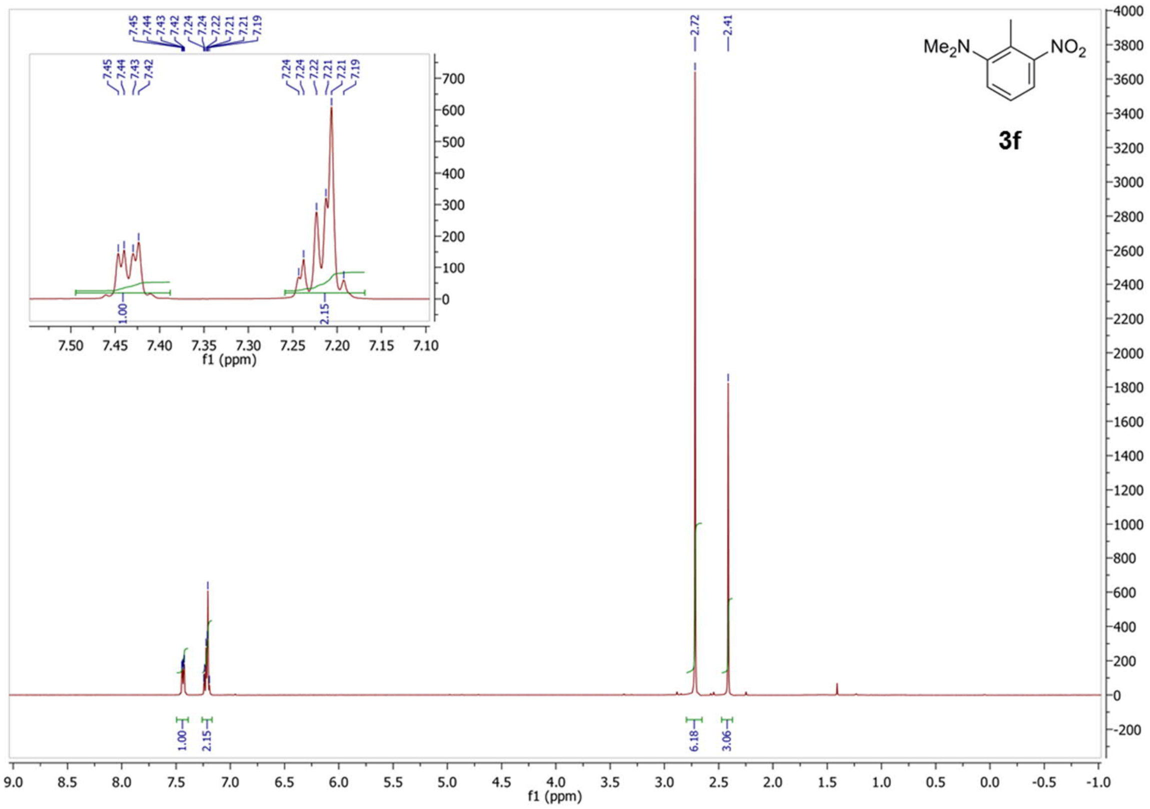

Figure S50. ^{1}H NMR (400 MHz) spectrum of 6-dimethylamino-2-nitrotoluene **3f** in $CDCl_3$.

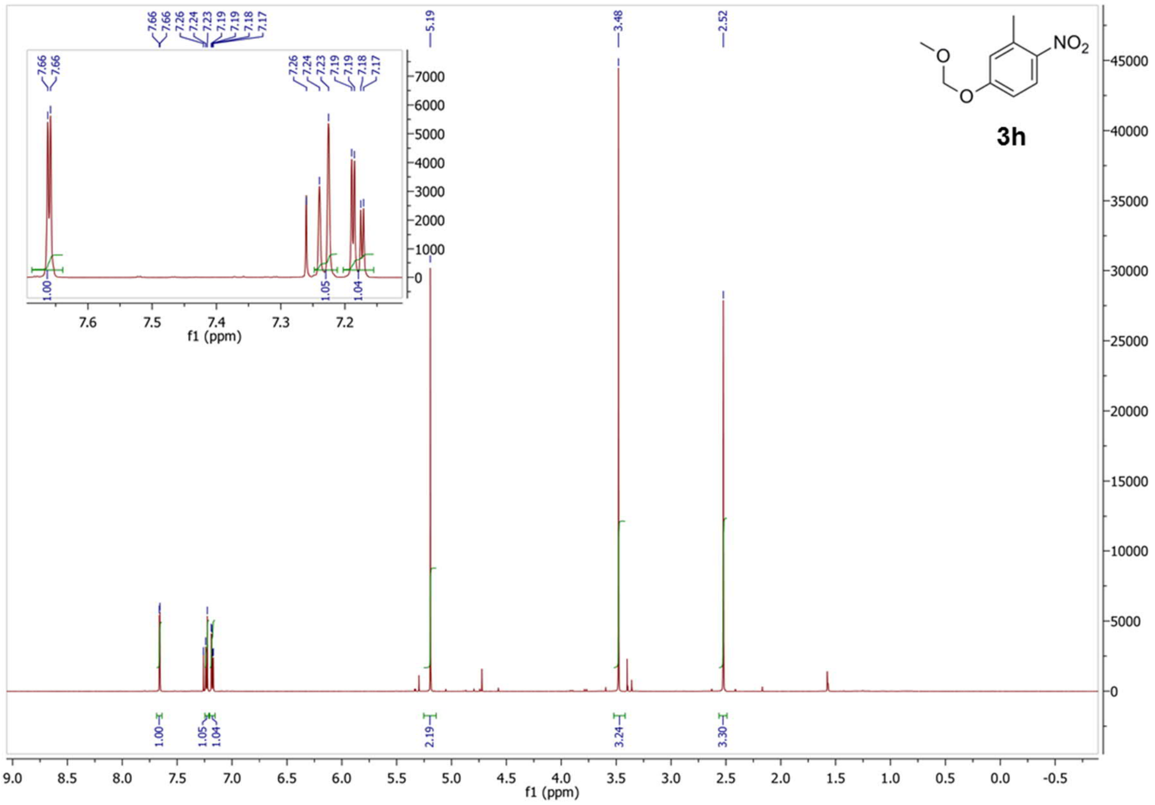

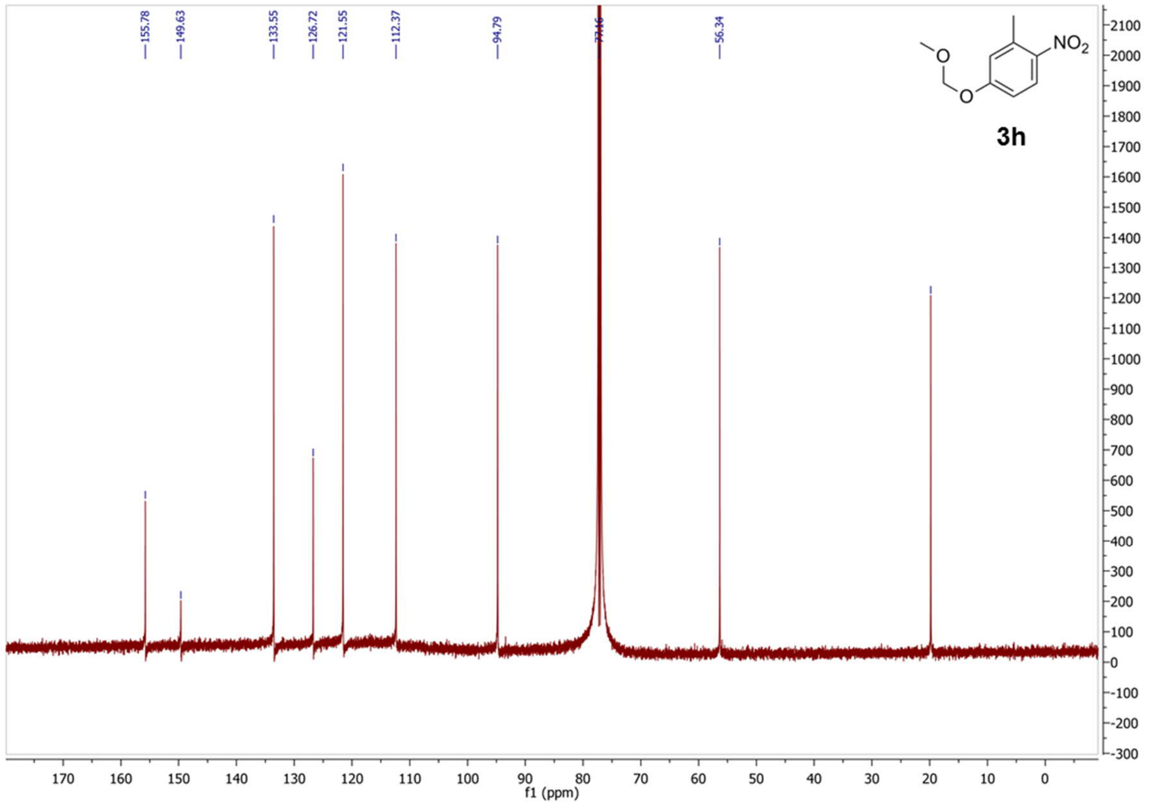

Figure S51. ^{1}H NMR (400 MHz) and ^{13}C NMR (101 MHz) spectra of 5-(methoxymethoxy)-2-nitrotoluene **3h** in $CDCl_3$.

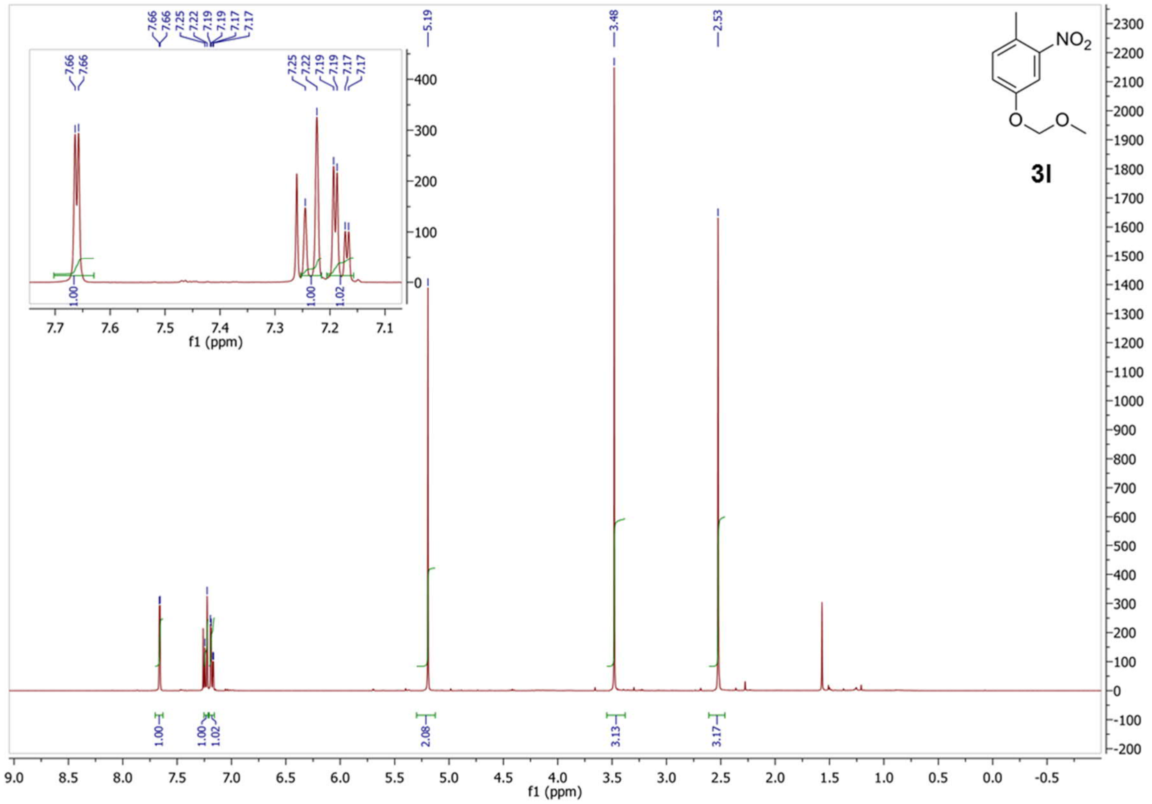

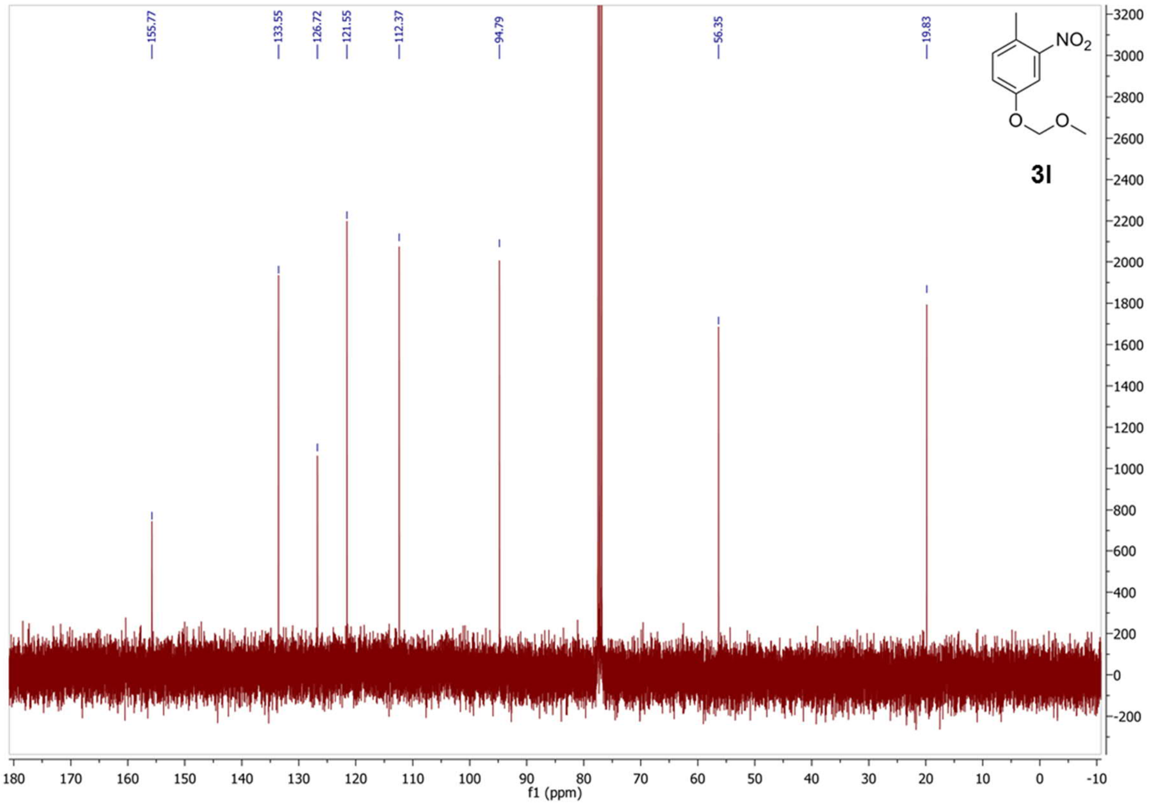

Figure S52. ^{1}H NMR (400 MHz) and ^{13}C NMR (101 MHz) spectra of 4-(methoxymethoxy)-2-nitrotoluene **3l** in $CDCl_3$.
